# Clinical practice guidelines: Oral health care for children and adults living with epidermolysis bullosa

**DOI:** 10.1111/scd.12511

**Published:** 2020-11-17

**Authors:** Susanne Krämer, James Lucas, Francisca Gamboa, Miguel Peñarrocha Diago, David Peñarrocha Oltra, Marcelo Guzmán‐Letelier, Sanchit Paul, Gustavo Molina, Lorena Sepúlveda, Ignacio Araya, Rubén Soto, Carolina Arriagada, Anne W Lucky, Jemima E Mellerio, Roger Cornwall, Fatimah Alsayer, Reinhard Schilke, Mark Adam Antal, Fernanda Castrillón, Camila Paredes, Maria Concepción Serrano, Victoria Clark

**Affiliations:** ^1^ Facultad de Odontología Universidad de Chile Santiago Chile; ^2^ Dental Department Royal Children's Hospital Melbourne Australia; ^3^ Stomatology Department Faculty of Medicine and Dentistry University of Valencia Spain; ^4^ Hospital Base Valdivia Valdivia Chile; ^5^ Facultad de Odontologia Universidad San Sebastián Valdivia Chile; ^6^ Nurturing Healthy Smiles Greater Noida India; ^7^ Universidad Nacional de Córdoba Argentina; ^8^ Universidad Católica de Córdoba Argentina; ^9^ Hospital Santiago Oriente Maxillofacial Surgery Unit Chile; ^10^ Cincinnati Children's Epidermolysis Bullosa Center Cincinnati Children's Hospital Cincinnati Ohio USA; ^11^ The University of Cincinnati College of Medicine Cincinnati Ohio USA; ^12^ St John's Institute of Dermatology Guy's and St Thomas’ NHS Foundation Trust London UK; ^13^ Royal National ENT and Eastman Dental Hospitals University College London Hospitals London UK; ^14^ Hannover Medical School Department of Conservative Dentistry Periodontology and Preventive Dentistry Hannover Germany; ^15^ University of Szeged Faculty of Dentistry Szeged Hungary; ^16^ Private practice Valencia Spain; ^17^ Birmingham Children's Hospital UK

**Keywords:** clinical practice guideline, dental implants, dental treatment, dystrophic epidermolysis bullosa, epidermolysis bullosa, epidermolysis bullosa simplex, general anesthesia, junctional epidermolysis bullosa, kindler epidermolysis bullosa, oral care, oral rehabilitation, recessive dystrophic epidermolysis bullosa, sedation

## Abstract

**Background:**

Inherited epidermolysis bullosa (EB) is a genetic disorder characterized by skin fragility and unique oral features.

**Aims:**

To provide (a) a complete review of the oral manifestations in those living with each type of inherited EB, (b) the current best practices for managing oral health care of people living with EB, (c) the current best practices on dental implant‐based oral rehabilitation for patients with recessive dystrophic EB (RDEB), and (d) the current best practice for managing local anesthesia, principles of sedation, and general anesthesia for children and adults with EB undergoing dental treatment.

**Methods:**

Systematic literature search, panel discussion including clinical experts and patient representatives from different centers around the world, external review, and guideline piloting.

**Results:**

This article has been divided into five chapters: (i) general information on EB for the oral health care professional, (ii) systematic literature review on the oral manifestations of EB, (iii) oral health care and dental treatment for children and adults living with EB—clinical practice guidelines, (iv) dental implants in patients with RDEB—clinical practice guidelines, and (v) sedation and anesthesia for adults and children with EB undergoing dental treatment—clinical practice guidelines.

Each chapter provides recommendations on the management of the different clinical procedures within dental practice, highlighting the importance of patient‐clinician partnership, impact on quality of life, and the importance of follow‐up appointments. Guidance on the use on nonadhesive wound care products and emollients to reduce friction during patient care is provided.

**Conclusions:**

Oral soft and hard tissue manifestations of inherited EB have unique patterns of involvement associated with each subtype of the condition. Understanding each subtype individually will help the professionals plan long‐term treatment approaches.

## CHAPTER ORDER

1. General information on epidermolysis bullosa for the oral health care professional
2. Systematic literature review: oral manifestations of epidermolysis bullosa
3. Oral health care and dental treatment for children and adults living with epidermolysis bullosa—clinical practice guidelines
4. Dental implants in patients with recessive dystrophic epidermolysis bullosa—clinical practice guidelines
5. Sedation and anesthesia for adults and children with epidermolysis bullosa undergoing dental treatment—clinical practice guidelines

### Guideline development group and responsibilities

**TABLE 1:** Guideline development group: clinical leads

Prof. Dr. Susanne Krämer Associate Professor in Special Care Dentistry, Universidad de Chile. Dentist, DEBRA Chile.	Clinical lead
Prof. Dr. James Lucas Deputy Director, Dental Department, Royal Children's Hospital, Melbourne, Australia. Clinical Associate Professor, University of Melbourne, Australia.	Clinical lead

**TABLE 2:** Guideline development group: clinical experts

Prof. Dr. Miguel Pañarrocha Chairman of Oral Surgery, Stomatology Department, Faculty of Medicine and Dentistry, University of Valencia, Spain.	Clinical expert chapters 3 and 4 External review chapter 5
Mrs. Victoria Clark Birmingham Children's Hospital, Birmingham, United Kingdom.	Chapter 4
Prof. Dr. David Peñarrocha Assistant Professor of Oral Surgery, Stomatology Department, Faculty of Medicine and Dentistry, University of Valencia, Spain.	Clinical expert chapters 3 and 4 External review chapter 5
Dr. Francisca Gamboa Assistant Professor in Special Care Dentistry, Universidad de Chile.	Chapters 3‐5
Prof. Dr. Marcelo Guzmán‐Letelier MSc, DDS, Department of Oral and Maxillofacial Surgery, Hospital Base Valdivia. Valdivia, Chile. Assistant Professor Department of Dentistry, San Sebastian University Dental School. Valdivia, Chile.	Chapters 3 and 4
Dr. Sanchit Paul Founder & Chief Pediatric Dentist‐ Tooth Tales: Nurturing Healthy Smiles, Greater Noida, India.	Chapters 3 and 4
Prof. Dr. Gustavo Molina Facultad de Odontología, Universidad Nacional de Córdoba. Carrera de Odontología, Facultad de Ciencias de la Salud, Universidad Católica de Córdoba. Argentina.	Clinical expert chapters 3 and 4 External review chapter 5
Ms. Lorena Sepúlveda Speech and Language therapist, Special Care Clinic. Facultad de Odontología, Universidad de Chile.	Clinical expert chapters 3 and 4 External review chapter 5
Dra. María Concepción Serrano Médico Estomatólogo, Universidad de Murcia. Doctora en Medicina y Cirugía Universidad de Valencia. Postgrado en Pacientes Especiales, Universidad de Valencia, Spain. Private practice in Valencia, Spain.	Chapter 4
Prof. Dr. Anne W Lucky, MD Acting Director, Division of Pediatric Dermatology Medical Director, Cincinnati Children's Epidermolysis Bullosa Center, Cincinnati Children's Hospital, USA. Professor of Dermatology and Pediatrics, The University of Cincinnati College of Medicine, Cincinnati, Ohio, USA	Chapter 5
Dr. Jemima E Mellerio, MD FRCP Consultant Dermatologist and Honorary Professor of Pediatric Dermatology, St John's Institute of Dermatology, Guy's and St Thomas’ NHS Foundation Trust, London, UK.	Chapter 5
Dr. Roger Cornwall Professor of Orthopedic Surgery and Developmental Biology Cincinnati Children's Hospital Medical Centre, Cincinnati, OH USA.	Chapter 5

**TABLE 3:** Guideline development group: patient representatives

Scott O'Sullivan, DEBRA United Kingdom.	Panel member, Chapters 3 and 4
Toni Roberts, South African EB Interest Group.	Panel member, Chapters 3 and 4
Lisa Brains, Australia.	External review chapters 3‐5
Anna Carolina Rocha, Brazil.	External review chapters 3‐5
Kerry Thompson, Australia.	External review chapters 3‐5
Rebecca Bodan, United States.	External review chapter 3
May Dijkgraaf, DEBRA Malaysia.	External review chapters 3‐5
Carol Somoza, Spain.	External review chapter 3 and 4

**TABLE 4:** Guideline development group: methodologists

Dr. Ignacio Araya Department of Oral and Maxillofacial Surgery, Evidence based Dentistry Unit, Universidad de Chile	Methodologists Chapters 2‐5
Dr. Ruben Soto Lecturer in Special Care Dentistry, Universidad de Chile.	Methodologists Chapters 3‐5
Dr. Carolina Arriagada Lecturer in Special Care Dentistry, Universidad de Chile.	Methodologists Chapters 3‐5
Fernanda Castrilón and Camila Paredes Researcher, Universidad de Chile	Methodologists Chapters 2 and 3

**TABLE 5:** Guideline development group: external reviewers

Prof. Dr. Timothy Wright Bawden Distinguished Professor of Pediatric Dentistry University of North Carolina at Chapel Hill School of Dentistry, USA.	Chapters 3 and 5
Dr. Urshla Devalia Consultant Paediatric Dentist, Great Ormond Street Hospital for Children & Eastman Dental Hospital and Institute, UK.	Chapter 3
Giulio Fortuna, DMD, PhD Specialist in Oral Medicine, Specialist in General Practice Dentistry, Diplomate, American Board of Oral Medicine, Editor‐in‐Chief, American Journal of Oral Medicine. USA.	Chapters 3‐5
Dr. Pallavi Urs Associate Professor, Department of Pedodontics and preventive dentistry, Krishnadevaraya College of Dental Sciences, Bangalore, India.	Chapter 3
Dr. Reinhard Schilke Senior Physician at Hannover Medical School, Hannover, Germany.	Chapters 3‐5
Prof. Stephen Porter, MD PhD Institute Director and Professor of Oral Medicine, UCL Eastman Dental Institute, London, UK.	Chapter 5
Dr. Chris Dickinson, BDS, LDS, MSc, MFDS Consultant Guy's & St. Thomas NHS Foundation Trust, London, United Kingdom.	Chapter 5
Dr. Marcelo Valle Assistant Professor in Special Care Dentistry, Universidad de Chile.	Chapters 3 and 4
Dr. Paulina Ledezma Endodontist, Special Care Dentistry Clinic, Universidad de Chile.	Chapter 3
Dr. Sebastián Veliz Orthodontist, Special Care Dentistry Clinic, Assistant Professor, Universidad de Chile.	Chapters 3‐5
Natalie Yerlett Advanced Practitioner EB and rare dermatology Dietitian, Great Ormond Street Hospital, London, United Kingdom.	Chapters 3 and 5
Dr. Arturo Kantor, M.D. Cornea Fellow, University of Iowa Hospitals and Clinics. Medical Director, Centro de la Visión, Clínica las Condes, Santiago, Chile	Chapter 5
Dr. Maria Joao Yuber, MD Pediatrician and Infectious Disease Specialist, Debra Chile, Chile.	Chapter 5

**TABLE 6:** Guideline development group: Pilot Centers

Mrs. Victoria Clark Birmingham Children's Hospital, Birmingham, United Kingdom.	Chapters 3 and 5
Dr Erin Mahoney Hutt Valley DHB Wellington, New Zealand.	Chapter 3
Dr. PhD Mark Adam Antal University of Szeged, Faculty of Dentistry, Szeged, Hungary.	Chapters 3‐5
Prof. Dr. Hao‐Hung Chang National Taiwan University Hospital, Taipei, Taiwan.	Chapters 3‐5

### Funding for the Guideline

The Guideline was funded by a grant from DEBRA UK (panel meetings and operational costs). The views or interests of the funding body have not influenced the final recommendations.

### Conflicts of Interest

None of the authors declared conflict of interest. None of the authors has any connection to manufacturers.

### Disclaimer

The recommendations contained in these guidelines do not indicate an exclusive course of action or serve as a standard medical care. Variations, taking individual circumstances into account, may be appropriate. The authors of these guidelines have made considerable efforts to ensure that the information upon which they are based is accurate and up to date. Users of these guidelines are strongly recommended to confirm the information contained within them. The authors, DEBRA UK or DEBRA International accept no responsibility for any inaccuracies, information perceived as misleading, or the success of any treatment regimen detailed in the guidelines.

## Introduction

Inherited epidermolysis bullosa (EB) is a genetic disorder characterized by skin fragility. Affected individuals present unique oral features, requiring a special approach from the dental team.

The International Dystrophic Epidermolysis Bullosa Research Association (DEBRA International) is the worldwide network of national groups working on behalf of those affected by EB. As part of their vision for working to ensure access to the best quality support and medical care for people living with EB, DEBRA International entrusts the development of clinical practice guideline (CPG) to health care professionals with significant experience in EB around the world. In 2012, the first CPG on oral health care for patients with EB was published.^1^ New literature reviews, case series, and case reports have been published. It has become necessary to update the guideline including the new evidence, as well as including more experts from different centers around the world.

Considering the new information and wider scope of treatment alternatives, the present update has been divided into five chapters: (i) general information on EB for the oral health care professional (update), (ii) systematic literature review of oral manifestations of EB (update), (iii) CPG on oral health care for children and adults living with EB (update), (iv) dental Implants in patients with recessive dystrophic EB (new guideline), and (v) sedation and anesthesia for patients with EB undergoing dental care (update).

REFERENCE1

Krämer
SM
, 
Serrano
MC
, 
Zillmann
G
, et al. Oral health care for patients with epidermolysis bullosa ‐ best clinical practice guidelines. Int J Paediatr Dent.
2012;22:1‐35.2293790810.1111/j.1365-263X.2012.01247.x

## CHAPTER 1: General information on epidermolysis bullosa for the oral health care professional

Susanne Krämer

Inherited epidermolysis bullosa (EB) is a group of genetic disorders with skin fragility and blistering. Clinically, it is highly heterogeneous, presenting blisters and erosions not only on skin, but also on mucous membranes as well as affecting other tissues. It is caused by variants in the genes encoding proteins of the dermal‐epidermal adhesion zone.^1^ Acquired forms of EB, caused by autoantibodies to type VII collagen, are known as Epidermolysis Bullosa Acquisita (EBA). This guideline will only discuss the inherited types of EB.

### 1.1 Diagnosis and classification

EB presents a wide range of clinical phenotypes with thousands of sequence variants identified in at least 16 structural genes.^1^, ^2^ Classification schemes were first introduced by Pearson in 1962.^3^ Since then various consensus classifications have been published.^1^, ^4^, ^5^, ^6^, ^7^ The current classification system has an “onion skin” approach. First, the major type is diagnosed based on the level of blister formation into: (a) EB simplex (EBS), (b) junctional EB (JEB), (c) dystrophic EB (DEB), and (d) Kindler EB (KEB, previously known as Kindler syndrome). Then the subclassification considers the clinical phenotypic features such as distribution (localized vs generalized), relative severity of cutaneous and extracutaneous involvement, mode of transmission, and specific gene involved. The 2020 classification system recognizes four major types, 35 subtypes, and five other disorders with skin fragility.^1^ The latest consensus reclassification published in February 2020 introduces the concept of genetic disorders with skin fragility and separates a category of “EB‐related” disorders.^1^ The main laboratory test to reach a diagnosis is immunofluorescence mapping (IFM) and genetic testing,^8^ helping to identify the protein that is altered or missing and the gene affected.

Dentists, as part of the multidisciplinary team, need to know and understand the complexity of the patient's diagnosis, in order to understand the prognosis and plan the treatment. When reporting a case in the literature, diagnostic information including EB type, subtype, and method used to obtain the diagnosis should be reported whenever available.

**TABLE 1.1:** List of abbreviations

EB	Epidermolysis Bullosa
EBS	EB Simplex
JEB	Junctional EB
DEB	Dystrophic EB
RDEB	Recessive DEB
DDEB	Dominant DEB
Sev RDEB	Severe RDEB
KEB	Kindler EB
DEBRA	Dystrophic Epidermolysis Bullosa Research Association
DI	DEBRA International
CPG	Clinical practice guideline
SCC	Squamous cell carcinoma
OSSC	Oral squamous cell carcinoma
IFM	Immunofluorescence mapping

### 1.2 Epidemiology

The estimated incidence of inherited EB is 19.6 per 1 million live births (about 1:50.000) and the prevalence is 11 cases per million inhabitants.^9^ Each type and subtype of EB has a different prognosis. Fine and coworkers analyzed the cumulative risk of death of children with EB.^10^ Important difference can be observed among types and subtypes. While no deaths occurred during the first 15 years of life in patients with localized EBS and dominant dystrophic EB (DDEB), the cumulative risk of death at the age of 1 was 2.8% in severe EBS and 40% in JEB. By the age of 15, the cumulative risk of death was 62% in severe JEB and 8% in severe recessive dystrophic EB (RDEB). The main causes are sepsis, failure to thrive, and respiratory failure.^10^ In adults, the main causes of death are cardiomyopathy, renal failure, and squamous cell carcinoma (SCC).^11^ SCC is the leading cause of death in patients with RDEB,^12^ particularly the severe form of RDEB with a cumulative risks of death from SCC of 38.7%, 70.0%, and 78.7% by ages 35, 45, and 55, respectively.^13^

### 1.3 General clinical manifestations

**IMAGE 1.1:**
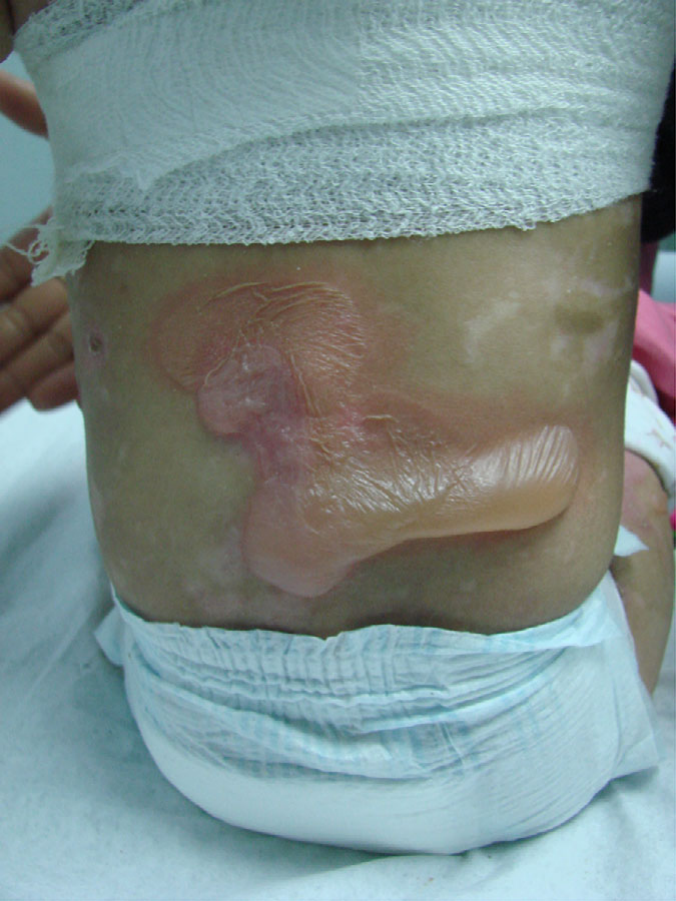
Extensive bullae covering the back of a patient with RDEB

The hallmark feature of inherited EB is mechanical fragility of the skin and the appearance of blisters and bullae (Image 1.1). In most forms of EB, tense blisters form with clear, colorless exudate or occasionally hemorrhagic fluid, eventually giving rise to eroded areas.^14^ The blisters and erosions can occur as a result of trauma but may also arise spontaneously and can be exacerbated by sweating and warmer climates.^15^ Other findings include milia, dystrophy or absence of nails, alopecia, exuberant granulation tissue, congenital absence of skin, palmoplantar keratoderma, mottled pigmentation, and pigmented naevi. Secondary skin lesions are cutaneous atrophy, scarring, pigmentary abnormalities, webbing, and contractures that can each arise secondary to the vesiculobullous and erosive lesions.^14^

**IMAGE 1.2:**
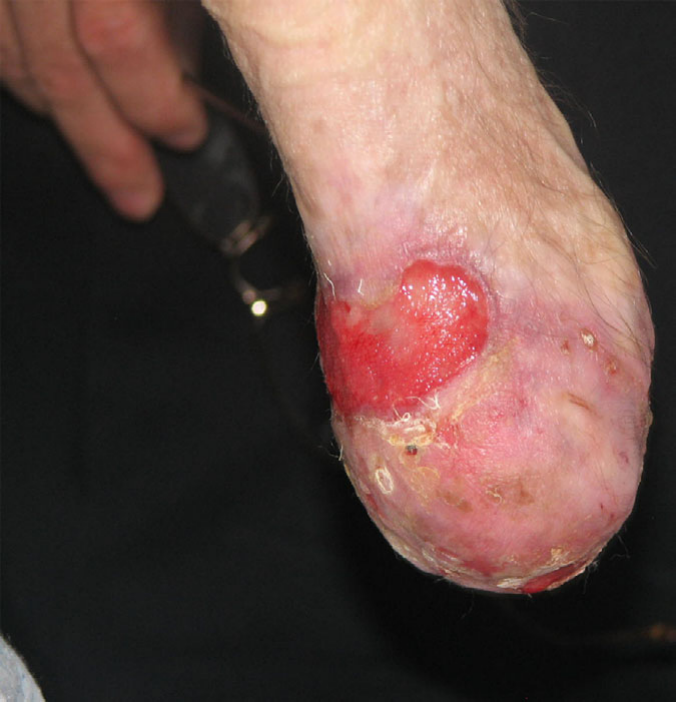
Squamous cell carcinoma in RDEB

SCC of the skin is one of the most severe complications of EB, starting to arise in early adulthood in patients with the severe forms of EB, particularly severe generalized recessive dystrophic EB (RDEB sev‐gen) (Image 1.2). SCC can present as (a) a nonhealing wound; (b) a rapidly growing wound, especially one that is heaped up, resembling exuberant granulation tissue; (c) a deep, punched‐out ulcer, especially if it has a raised or rolled edge; (d) an area of hyperkeratosis, especially if surrounded by a shoulder of raised skin; and (e) a wound with altered sensation relative to normal EB wounds (eg, tingling or increased pain).^12^

#### 1.3.1 Eyes, ears, nose, and throat

Ocular findings include corneal blisters and erosions, corneal scarring, pannus formation, limbal broadening, conjunctival blisters, erosions, symblepharon, eyelid blisters and scars, ectropion, and lacrimal duct obstruction. Marked visual impairment can result from repeated injury to the cornea, especially if scarring develops. Signs and symptoms in the upper respiratory tract can include weak or hoarse cry, dysphonia, inspiratory stridor, soft tissue edema, vesiculation or blistering of all tracheolaryngeal structures and ulceration, thickening, and scarring of the true and false vocal cords.^14^

#### 1.3.2 Gastrointestinal complications

**IMAGE 1.3:**
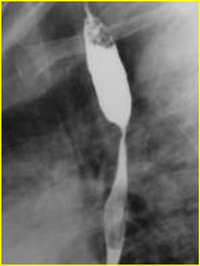
Severe esophageal stenosis in a patient with RDEB

EB‐associated esophageal strictures in the proximal area may arise, resulting in progressive dysphagia and requiring esophageal balloon dilatations (Image 1.3).^16^ This has an impact on dental care. Prescriptions need to consider the patient's ability to swallow. A prescription in liquid form should be considered.^17^ The most common lower gastrointestinal complaint in severe EB types is chronic constipation.^14^

#### 1.3.3 Acral deformities

**IMAGE 1.4:**
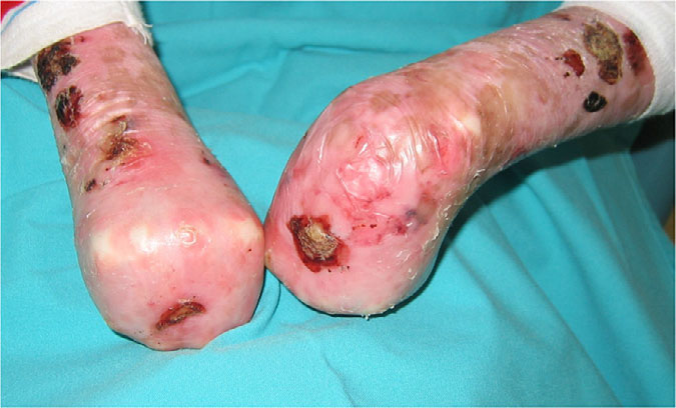
Mitten deformities in RDEB

Pseudosyndactyly is the most visible extracutaneous complication of inherited EB and is primarily seen in RDEB (Image 1.4). These progressive deformities can cause marked functional disability.^14^ These also have an impact on dental care, as ability to brush the teeth independently may be affected.^17^ Guidance on occupational therapy in EB can be found in the recently published CPG.^18^

#### 1.3.4 Other complications

Nutritional compromise is proportional to the severity of EB and occurs mainly in generalized form of RDEB and JEB.^19^, ^20^ Patients can also present anemia,^14^ dilated cardiomyopathy,^21^ osteoporosis, and osteopenia.^22^

### 1.4 Clinical care

DEBRA International has supported and funded the development of CPG in skin and wound care,^23^, ^24^ pain management,^25^ psychosocial care,^26^ foot care,^27^ as well as the guidelines that have already been cited in this article.^8^, ^12^, ^17^, ^18^ Research is also supported to explore gene, protein, and cell therapies. Updated information is continuously provided through the Charities web page.^28^

### 1.5 Quality of life in EB

In complex conditions, such as EB, understanding the burden in patient's quality of life is important. The main areas where individual with EB have described concerns include (a) having an itchy skin, (b) being in pain, (c) having difficulties with participation/joining others, (d) the visibility of the disease, and (e) the feeling of being different.^29^ A quality of life questionnaire specific for patients with EB (QOLEB) was developed by Frew, Murrell, and coworkers. The questionnaire contains 17 items and has proven to be a valid and reliable measurement tool. It can be used to monitor quality of life and to identify dimensions of QOL as targets for interventions and research.^30^

REFERENCES1

Has
C
, 
Bauer
JW
, 
Bodemer
C
, et al. Consensus reclassification of inherited epidermolysis bullosa and other disorders with skin fragility. Br J Dermatol. 2020;bjd.18921
10.1111/bjd.18921.320170152

Uitto
J
. Toward treatment and cure of epidermolysis bullosa. Proc Natl Acad Sci U S A.2019;116:26147‐26149. 10.1073/pnas.1919347117.PMC6936717318269523

Pearson
RW
. Studies on the pathogenesis of epidermolysis bullosa. J Invest Dermatol. 1962;39(6):551‐575.1394226510.1038/jid.1962.1564

Fine
JD
, 
Eady
RAJ
, 
Bauer
EA
, et al. The classification of inherited epidermolysis bullosa (EB): Report of the Third International Consensus Meeting on Diagnosis and Classification of EB. J Am Acad Dermatol. 2008;58(6):931‐950.1837445010.1016/j.jaad.2008.02.0045

Fine
JD
, 
Bauer
EA
, 
Briggaman
RA
, et al. Revised clinical and laboratory criteria for subtypes of inherited epidermolysis bullosa. A consensus report by the Subcommittee on Diagnosis and Classification of the National Epidermolysis Bullosa Registry. J Am Acad Dermatol. 1991;24(1):119‐135.199950910.1016/0190-9622(91)70021-s6

Fine
JD
, 
Eady
RA
, 
Bauer
EA
, et al. Revised classification system for inherited epidermolysis bullosa: Report of the Second International Consensus Meeting on diagnosis and classification of epidermolysis bullosa. J Am Acad Dermatol. 2000;42(6):1051‐1066.108274127

Fine
JD
, 
Bruckner‐Tuderman
L
, 
Eady
RAJ
, et al. Inherited epidermolysis bullosa: Updated recommendations on diagnosis and classification. J Am Acad Dermatol. 2014;70(6):1103‐1126.2469043910.1016/j.jaad.2014.01.9038

Has Id
C
, 
Liu
L
, 
Bolling
MC
, et al. Clinical practice guidelines for laboratory diagnosis of epidermolysis bullosa Funding sources. Br J Dermatol. 2020;182(3):574‐592.3109006110.1111/bjd.18128PMC70649259

Fine
J‐D
. Epidemiology of inherited epidermolysis bullosa based on incidence and prevalence estimates from the National Epidermolysis Bullosa Registry. JAMA Dermatol. 2016;152(11):1231.2746309810.1001/jamadermatol.2016.247310

Fine
JD
, 
Johnson
LB
, 
Weiner
M
, 
Suchindran
C
. Cause‐specific risks of childhood death in inherited epidermolysis bullosa. J Pediatr. 2008;152(2):276‐280.1820670210.1016/j.jpeds.2007.06.03911

Hon
KLE
, 
Li
JJ
, 
Cheng
BL
, et al. Age and etiology of childhood epidermolysis bullosa mortality. J Dermatolog Treat. 2015;26(2):178‐182.2472459610.3109/09546634.2014.91500212

Mellerio
JE
, 
Robertson
SJ
, 
Bernardis
C
, et al. Management of cutaneous squamous cell carcinoma in patients with epidermolysis bullosa: best clinical practice guidelines. Br J Dermatol. 2016;174(1):56‐67.2630213710.1111/bjd.1410413

Fine
J‐D
, 
Johnson
LB
, 
Weiner
M
, 
Li
K‐P
, 
Suchindran
C
. Epidermolysis bullosa and the risk of life‐threatening cancers: the National EB Registry experience, 1986–2006. J Am Acad Dermatol. 2009;60(2):203‐211.1902646510.1016/j.jaad.2008.09.03514

Lanschuetzer
CM
, 
Fine
J‐D
, 
Laimer
M
, et al. General aspects In: FineJ‐D, HintnerH, eds. Life with Epidermolysis Bullosa (EB). Vienna: Springer Vienna; 2009:1‐95. 10.1007/978-3-211-79271-1_1
15

Schaffer
SR
. Head and neck manifestations of epidermolysis bullosa. Clin Pediatr (Phila). 1992;31(2):81‐88.154428010.1177/00099228920310020416

Anderson
BT
, 
Feinstein
JA
, 
Kramer
RE
, et al. The approach and safety of esophageal dilation for treatment of strictures in children with epidermolysis bullosa HHS public access. J Pediatr Gastroenterol Nutr. 2018;67(6):701‐705.3005256710.1097/MPG.0000000000002106PMC624908617

Krämer
SM
, 
Serrano
MC
, 
Zillmann
G
, et al. Oral health care for patients with epidermolysis bullosa ‐ best clinical practice guidelines. Int J Paediatr Dent. 2012;22(Suppl. 1):1‐35.2293790810.1111/j.1365-263X.2012.01247.x18

Chan
JM
, 
Weisman
A
, 
King
A
, et al. Occupational therapy for epidermolysis bullosa: clinical practice guidelines. Orphanet J Rare Dis. 2019;14(1):129.3117455910.1186/s13023-019-1059-8PMC655602119

Fine
J‐D
, 
Johnson
LB
, 
Weiner
M
, 
Suchindran
C
. Gastrointestinal complications of inherited epidermolysis bullosa: cumulative experience of the national epidermolysis bullosa registry. J Pediatr Gastroenterol Nutr. 2008;46(2):147‐58.1822337310.1097/MPG.0b013e31812f566720

Haynes
L
. Nutritional support for children with epidermolysis bullosa In: FineH‐D, HintnerH, eds. Life with Epidermolysis Bullosa (EB): Etiology, Diagnosis, Multidisciplinary Care and Therapy. Vienna: Springer Vienna; 2008:258‐277.21

Sidwell
RU
, 
Yates
R
, 
Atherton
D
. Dilated cardiomyopathy in dystrophic epidermolysis bullosa. Arch Dis Child. 2000;83(1):59‐63.1086900110.1136/adc.83.1.59PMC171838622

Fewtrell
MS
, 
Allgrove
J
, 
Gordon
I
, et al. Bone mineralization in children with epidermolysis bullosa. Br J Dermatol. 2006;154(5):959‐962.1663490110.1111/j.1365-2133.2005.07123.x23

Pope
E
, 
Lara‐Corrales
I
, 
Mellerio
J
, et al. A consensus approach to wound care in epidermolysis bullosa. J Am Acad Dermatol. 2012;67(5):904‐917.2238703510.1016/j.jaad.2012.01.016PMC365540324

Denyer
J
, 
Pillay
E
, 
Clapham
J
. Best Practice Guidelines Skin and wound care in Epidermolysis Bullosa [Internet]. London; 2017
www.woundsinternational.com.25

Goldschneider
KR
, 
Good
J
, 
Harrop
E
, et al. Pain care for patients with epidermolysis bullosa: best care practice guidelines. BMC Med. 2014;12(1):1‐23.10.1186/s12916-014-0178-2PMC41905762560387526

Martin
K
, 
Geuens
S
, 
Asche
JK
, et al. Psychosocial recommendations for the care of children and adults with epidermolysis bullosa and their family: evidence based guidelines. Orphanet J Rare Dis. 2019;14(1):133.3118606610.1186/s13023-019-1086-5PMC656072227

Khan
MT
, 
Faitli
B
, 
Mellerio
JE
, et al. Foot care in epidermolysis bullosa: evidence‐based guideline. Br J Dermatol. 2020;182(3):593‐604.3139788210.1111/bjd.18381PMC706508928
Debra International Homepage [Internet]
. Retrieved 2020 March 20, from http://www.debra-international.org/homepage.html.29

van Scheppingen
C
, 
Lettinga
A
, 
Duipmans
J
, 
Maathuis
C
, 
Jonkman
MF
. Main problems experienced by children with epidermolysis bullosa: a qualitative study with semi‐structured interviews. Acta Derm Venereol. 2008;88(2):143‐150.1831144210.2340/00015555-037630

Frew
JW
, 
Martin
LK
, 
Nijsten
T
, 
Murrell
DF
. Quality of life evaluation in epidermolysis bullosa (EB) through the development of the QOLEB questionnaire: an EB‐specific quality of life instrument. Br J Dermatol. 2009;161(6):1323‐1330.1968187510.1111/j.1365-2133.2009.09347.x

## CHAPTER 2: Oral manifestations of epidermolysis bullosa: Systematic literature review

Susanne Krämer | Francisca Gamboa | Ignacio Araya | Fernanda Castrillón | Camila Paredes | Fatimah Alsayer | Victoria Clark

### Introduction

Children and adults living with inherited epidermolysis bullosa (EB) present unique oral features related to their specific EB type and subtype. These arise as a consequence of the functional abnormality of the proteins in their basement membrane. The Oral Health Care for Patients with Epidermolysis Bullosa ‐ Best Clinical Practice Guidelines^1^ published in 2012 included a review of the literature on the oral characteristics of the condition. A new systematic literature review became necessary, as new reviews, case series, and case reports have been published.

### Aim

The aim of this chapter is to provide a complete revision of the wide spectrum of oral manifestations present in people diagnosed with inherited EB. As such, this article considers information for all four major types of EB: EB Simplex, Junctional EB, Dystrophic EB, and Kindler EB.

### Methods

#### Eligibility criteria

Articles in which the main topics are oral care and precautions during dental treatment (diagnosis, and⁄or treatment and⁄or prognosis) of patients with EB, published from 1947 to March 2020 in any language.

#### Information sources

The literature search ranged from 1947 to March 2020. Consulted sources included the electronic databases PUBMED (1966 to March 31, 2020), EMBASE (1947 to March 31, 2020), Cochrane Database of Systematic Reviews (1992 to March 31, 2020), and the Cochrane‐controlled trials register (CENTRAL) (1992 to March 31, 2020). Dissertations, conference proceedings, technical reports, and other unpublished documents that meet the selection criteria were also included. The reference lists of all papers for relevant citations were reviewed. When all the relevant studies were identified, they were sent to the experts to review for completeness.

#### Search strategy

To identify studies for this review, detailed search strategies were developed for each database. These were based on the search strategy developed for PUBMED and revised appropriately for each database.

The search strategy used a combination of controlled vocabulary and free text terms based on:

#1 "Epidermolysis Bullosa"[Mesh]
#2 ((Epidermolysis[tiab] OR Acantholysis[tiab])) AND Bullosa[tiab]
#3 "Dentistry"[Mesh]
#4 "Oral Health"[Mesh]
#5 "Mouth Diseases"[Mesh]
#6 "Dentistry"[tiab]
#7 #1 OR #2
#8 #3 OR #4 OR #5 OR #6
#9 #7 AND #8

#### Study selection

Articles that included detailed information on the patient's EB diagnosis and description of oral features were considered, including case reports and case series. It was desirable for the reports to have the EB diagnoses confirmed by IFM or genetic testing; however, this was largely unavailable and could not be used as a selection criterion. The criteria used to reject articles at first‐stage screening (based on title and abstract) and second‐stage screening (based on a review of the full text) were: (a) The article does not relate to inherited EB. (b) The article describes inherited EB, but does not consider oral aspects. (c) The article describes inherited EB and oral aspects, but only dental treatment is detailed, without describing oral manifestations. (d) The article describes oral manifestations of inherited EB; however, the diagnosis of EB is not well justified or incomplete. (e) The article describes oral manifestations of inherited EB; however, the method to diagnose the oral manifestation is not standardized, well described, or incomplete. (f) Cohort already published in previous articles. (g) Literature review does not provide new clinical information.

#### Data collection process

Data were extracted in duplicate by two independent reviewers. The findings were discussed at a researchers’ consensus meeting.

#### Data items

The main variables were the types of EB types: (a) EB Simplex, (b) Junctional EB, (c) Dystrophic EB, and (d) Kindler EB. Whenever possible, the most detailed information on the subtype of EB was collected.

Within each patient description, the clinical features registered were:

1. Perioral tissue involvement
  - Microstomia (mouth opening)
2. Intraoral soft tissue involvement
  - Oral ulcers
  - Denuded tongue
  - Ankyloglossia
  - Vestibule obliteration
  - Oral cancer
  - Periodontal disease
  - Saliva
3. Hard tissue involvement
  - Caries
  - Enamel Hypoplasia (localized or generalized)
  - Failure of eruption
  - Occlusal abnormalities
  - Dental maturity
  - Facial growth
  - Bone health

Less frequent findings were also collected.

#### Risk of bias

The risk of bias is high, as most of the reports do not present detailed patient diagnosis information (ie, mutation description) and do not use standardized assessment forms (eg, methods for assessing ankyloglossia).

### Results

**FIGURE 2.1:**
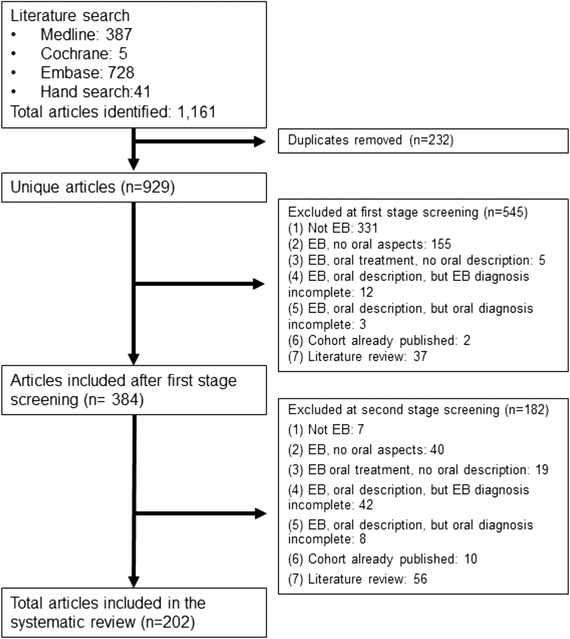
Flow chart of selected articles

The search strategy identified 1151 studies: 222 duplicates were removed, 545 articles excluded in first‐stage screening, 182 articles removed in second‐stage screening, and 202 articles were included in the systematic review (Figure 2.1).

#### Oral manifestations of EB

The frequency and severity of the oral features of EB vary according to the subtype of the disease. Most patients will present some type of vesiculobullous oral lesions, varying from small, discrete vesicles to large bullae and areas of granulation tissue. These lesions can be distributed on all the mucosal surfaces. Patients with the generalized RDEB are the most severely affected.^2^, ^3^

The involvement of dental hard tissues depends on the form of EB. Patients with JEB present with generalized enamel hypoplasia, individuals with RDEB have significantly more caries when compared with other EB types or unaffected controls,^2^ and those affected with Kindler EB have more periodontal disease.^4^

An early study of 101 individuals with EB demonstrated that oral blisters were present in 97% of patients with RDEB, 45% in dominant disease EB (DDEB), 37% in JEB, and 38% in EBS, while other features such as microstomia were present in 54% of the cases with RDEB, 7% of JEB and none of the patients with DDEB and EBS.^5^ Therefore, studying each type of EB is important to assess each patient's prognosis.

#### 2.1 EB simplex (EBS)

The most recent classification (2020) considers seven autosomal dominant and seven autosomal recessive subtypes of EB Simplex.^6^ Most of the literature on the oral aspects of EBS, however, precedes this classification. Therefore, the text will embrace EBS as a group and only describe specific subtype information when available. Anecdotally, one case of cleft lip and cleft palate of an infant with an EBS has been reported.^7^

##### Oral ulcers

Oral mucosal ulceration was described in 20% of patients with EBS in an early report.^8^ A more recent case series reported greater involvement, although oral mucosal involvement was not always determined by direct clinical examination but by a history of oral ulceration. A total of 40.3% of the group of 124 patients with EBS had oral ulcers with 58.6% of those with generalized and 34.7% with localized EB.^3^ Oral mucosal involvement was reported to be more common during the perinatal period, while in some patients, it persisted during early childhood or even later.^3^

##### Cancer risk

This literature review identified only one report of a 41‐year‐old patient with a recessive EBS who developed squamous cell carcinoma (SCC) on the tongue, at a site of frequent blistering^9^ and a single report of a 66‐year old man with a Merkel cell carcinoma on the right parotid.^10^

##### Localized EBS (EBS‐loc)

There is no agreement as to the frequency of oral mucosal lesions in EBS‐loc. While Sedano^11^ in 1989 reported that this subtype does not give rise to oral mucosal lesions, Wright in 1991 reported that 34.7% (33/95) of patients with localized EBS had a history of or presence of oral mucosal blisters at examination.^3^ Nine years later, in 2000, Horn studied a series on 54 patients and described that four individuals (7%) experienced intraoral blistering.^12^ Patients can present ulcers and erosions on their face.^13^

##### Intermediate EBS (EBS‐intermed)

It has been recognized that patients with this diagnosis may have occasional intraoral blisters, being less severe than those of other EB types.^11^ In a series of 69 individuals, 17 subjects (24%) experienced oral blisters.^12^ Anecdotally, a case report of a 3‐year‐old child described several white lesions and ulcers of various sized on the buccal mucosa and gingiva, as well as several decayed teeth.^14^

##### EBS intermediate with cardiomyopathy

In 2016, mutations in the gen KLHL24 were first identified in patients with EBS.^15^ The latest EB consensus reclassification published in 2020 classifies patients with mutations in the KLHL24 gen as EBS intermediate with cardiomyopathy. In those patients who were reported in the first article, the oral mucosa was mildly affected.^15^ A recent study including seven patients reported that 43% of them had common oral ulceration.^16^

##### EBS Intermediate with muscular dystrophy

The oral description of individuals with this subtype of EBS caused by mutations in the gene *PLEC* encoding plectin includes hemorrhagic blistering of oral mucosa since birth^17^ and a case report of micrognathia, high‐arched palate, and poor dentition with erosions.^18^

##### Severe EBS (EBS‐sev)

Patients in this group present with more mucosal lesions than the localized and intermediate subtypes of EBS. A case series reported history of intraoral lesions in 58.6% (17of 29) individuals with severe EBS.^3^ The series reported by Horn in 2000 included seven patients with severe EBS: the four infants in the study had intraoral blistering and hoarse cry; there is no intraoral description of the other three patients. It is reported, however, that severity of blistering lessened during childhood and adolescence in all patients.^12^ Lalor in 2018 described that three of five patients had severe oral blisters as neonate, one only had oral blistering during infancy, and the fifth patients had no mucosal blistering.^19^ Single case reports have also described frequent oral blistering and lesions affecting all areas of the oral mucosa, even within a few hours after birth.^20^, ^21^, ^22^ Occasionally, these ulcers are so painful that the patients are not able to tolerate toothbrushing due to trauma to the mucosa.^21^ On the other hand, there are reports of patients who only report occasional oral blisters.^21^ Anecdotally, one patient with multiple natal teeth and extensive blistering on her body and around her mouth has been described.^23^

### 2.2 Junctional EB (JEB)

The latest EB classification scheme recognizes two major subtypes: severe JEB (previously known as JEB generalized severe, Herlitz JEB) and intermediate JEB (previously known as JEB generalized intermediate, non‐Herlitz JEB). The classification also recognizes other less common subtypes and syndromic disorders: localized JEB, inversa JEB, late onset JEB, laryngo‐onycho‐cutaneous syndrome (LOC Syndrome), JEB with pyloric atresia, and JEB with interstitial lung disease and nephrotic syndrome.^6^ Same as for EBS, most of the literature on the oral aspects of JEB precedes this classification. Precise description will be provided as available.

##### Peri‐oral tissue involvement

**IMAGE 2.1 and 2.2:**
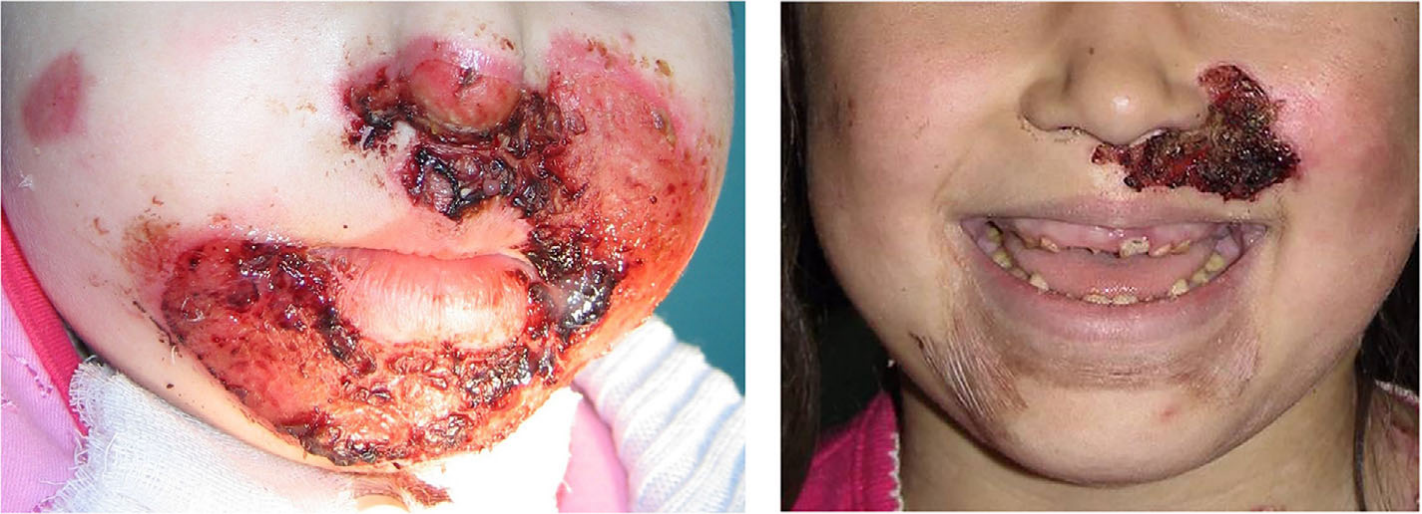
Areas of perioral granulation tissue in a 2‐ and 10‐year‐old patients with severe JEB

Peri‐oral and peri‐nasal granulation tissue lesions tend to develop between the 6th and 12th month of life in patients with severe JEB (Image 2.1). The lesions have been noted in all patients with severe JEB and tended to resolve during or after adolescence in patients who survived (Image 2.2).^3^, ^11^ They are believed to be pathognomonic for severe JEB.^11^

##### Microstomia

A case series studied the commissure‐to‐commissure distance obtaining: 39.2 mm in severe JEB, 46.7 mm in all the other JEB patients, and 44.7 mm in the healthy controls. Statistically, these differences were not significant.^3^ Other studies on oral functions in EB have revealed that 50% (3/6)^24^ and 67% (2/3)^13^ of the patients had limited mouth opening.

##### Intraoral soft tissue involvement

Patients with JEB seem to present with fewer mucosal lesions on examination.^25^ However, most patients will have a positive history of major oral mucosal bullae or intraoral areas of granulation tissue (83.3% in severe JEB, 91.6% in intermediate JEB).^3^, ^26^ These lesions might take several weeks or months to heal. Some will even take years to heal. Intraoral scarring is uncommon.^2^, ^3^, ^27^

##### Hard tissue involvement

###### Generalized enamel hypoplasia

**IMAGE 2.3 and 2.4:**
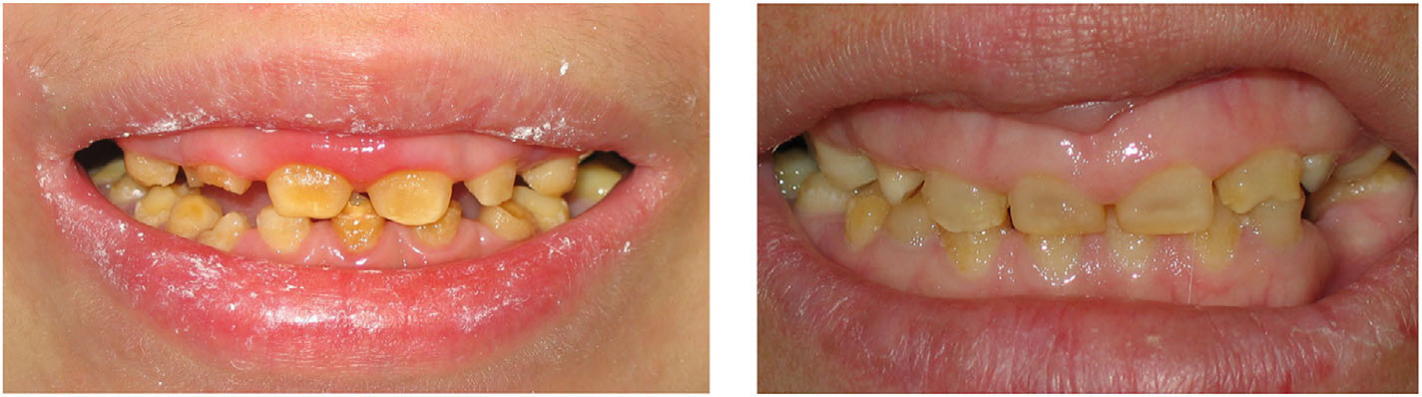
Generalized enamel hypoplasia in patients with JEB

**IMAGE 2.5:**
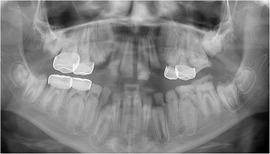
Panoramic radiograph showing thin, abnormal, severely hypoplastic enamel on both dentitions of a 10‐year‐old patient with JEB and generalized enamel hypoplasia

Generalized enamel hypoplasia has been reported in 59 case reports of individuals with JEB,^2^, ^25^, ^27^, ^28^, ^29^, ^30^, ^31^, ^32^, ^33^, ^34^, ^35^, ^36^, ^37^, ^38^, ^39^, ^40^, ^41^, ^42^, ^43^, ^44^, ^45^ as well as 100% of the patients with JEB in a series of cases (*n* = 6 severe JEB‐H, *n* = 19 other types of JEB) (Images 2.3 and 2.4).^46^ Enamel hypoplasia can be observed in panoramic radiographs as teeth with thin, abnormal, severely dystrophic enamel formation (Image 2.5).^25^ Some authors suggested that generalized enamel hypoplasia in EB is pathognomonic for JEB, and therefore, the teeth phenotype can be used as a guide to the EB type diagnosis when more precise laboratory tests are not available.^47^

The type or severity of enamel defects varies between individuals. In the series reported by Wright, 66.7% of the patients demonstrated generalized, rough, pitted enamel hypoplasia, while the remaining cases showed generalized thinning and/or furrowing of the enamel.^46^

Severe forms of JEB have shown a tendency to have thin (≈ 40 μm), prismless enamel.^46^, ^48^ While patients with other types of JEB, on the other hand, present a rather thicker but porous enamel with pits. The prismatic structure has been described as normal but interrupted by marked surface pitting.^46^, ^48^

Kirkham carried out a chemical characterization of the enamel of teeth from JEB patients and compared it to that of unaffected controls. The results showed that JEB enamel contained a significantly reduced mineral per volume content, resulting in enamel hypoplasia.^49^

Enamel hypoplasia have been described in patients with JEB caused by mutations in the genes encoding laminin‐332 (*LAMA3, LAMB3, and LAMC2*), α6β4‐integrin (*TGB4, ITGA6*), and type XVII collagen (*COL17A1*).^32^, ^36^, ^48^, ^50^, ^51^, ^52^, ^53^, ^54^ This has been explained due to the role of these proteins in cell adhesion in the odontogenic epithelium, which gives rise to ameloblasts, the cells that produce dental enamel. Laminin‐332 plays a vital role in all the stages of enamel formation. In the presecretory and maturation stages, it is part of the basal lamina and mediates adhesion of ameloblasts to the enamel matrix in the secretory stage. Abnormal ameloblast adhesion results in leakage of serum into the developing enamel leading to the retention of albumin that inhibits mineralization.^33^, ^55^, ^56^ The chemical characterization carried out by Kirkham revealed the presence of serum albumin in JEB enamel, in contrast to control enamel and enamel from patients with the dystrophic form of EB, where this was not detected.^49^, ^57^ Studies by Asaka showed that disruption of the *COL17A1* gene leads to abnormal interaction between enamel epithelium and the underlying mesenchyme, resulting in a defective ameloblast with a malformed Tomes’ processes with decreased secretion of enamel matrix at the secretary stage. At the maturation stage, this disruption in Col17 leads to a delayed calcification and reduced iron deposition in the enamel. These mechanisms contribute to an immature and irregular enamel formation.^58^

###### Failure of eruption

Failure of teeth eruption has been noted in three reports.^27^, ^39^, ^45^ Wright specifies that selected anterior and/or posterior teeth can be affected.^45^ This might be related to the gingiva hyperplasia that has been reported in 50% of the patients.^13^

##### Severe JEB

Oral lesions, including a history and/or presence of blisters, were reported in 83.3% of one group of patients with severe JEB.^3^ The reports of newborns suggest that blisters can develop during the first week of life.^59^ White plaques, ulcers and erosions on the gingivae, soft palate, hard palate, and lips have been reported in individual cases.^60^, ^61^

##### Less frequent findings

A rare case of pyogenic granuloma on the tongue was reported in young child with severe JEB that had undergone allogenic hematopoietic cell transplant. Both granulomas were successfully excised with no recurrence.^62^

##### Intermediate JEB

Oral lesions, including a history and/or presence of blisters, were reported in 91.6% of a group of 12 patients.^3^ Bullae might not be present at examination, but the patient can have a positive history of affected mucous membranes in the mouth.^63^

Hintner, in a report of the previously named *generalized atrophic benign epidermolysis bullosa*, GABEB reported blisters and ulcers on the oral mucosa during infancy, which caused difficulties eating and performing oral hygiene; but after puberty, the oral mucosal condition improved. Few patients had continuous blister formation on the oral mucous membranes. These blisters healed without scarring.^64^

A series of 12 patients with Intermediate JEB caused by mutations in the gene *COL17A1* coding for type XVII collagen described that all 12 patients had amelogenesis imperfecta (enamel pitting).^65^ In the same cohort of patients, it was reported that two patients occasionally had oral blisters, while a third patient had no mucous membranes involvement. There was no reported information on oral blisters from the other nine patients.^65^ The presence of enamel defects in carriers of mutations in *COL17A1* has been reported in two families.^66^, ^67^

Interestingly, in a family of a patient with Intermediate JEB due to a mutation in *LAMA3*, where the affected individual presented with occasional oral erosions and enamel hypoplasia, two healthy carriers of the *LAMA3* null mutations also had enamel defects, consisting of roughness and pits.^33^

##### JEB with pyloric atresia (JEB‐PA)

All the reports of patients with JEB‐PA describe generalized enamel hypoplasia^34^, ^35^ due to mutations in *ITGB4*, the gene encoding the ß4 integrin protein.^36^

##### Late onset JEB

The systematic literature search performed in this study only identified one paper describing oral features of late onset JEB. Although the report does not comply with the inclusion criteria of a well‐documented EB diagnosis (ideally an immunofluorescence or mutation analysis), it was decided to include the case as it represents the only evidence available for oral manifestations of this rare subtype of JEB. Two siblings with an electron microscopic study supporting JEB (blister formed between the dermis and epidermis above the dermal membrane) presented yellowish enamel defect of the entire dentition.^68^

##### LOC syndrome

Several reports have described generalized enamel hypoplasia, with small, yellow hypoplastic teeth.^69^, ^70^, ^71^, ^72^

### 2.3 Dystrophic EB (DEB)

DEB may be inherited as a dominant (DDEB) or recessive (RDEB) trait. Generally, RDEB is more severe than dominant disease (DDEB); however, there is considerable phenotypic overlap between types. It is caused by mutations in *COL7A1*, the gene coding collagen VII, the major component of the anchoring fibrils at the cutaneous basement membrane zone. The hallmark of DEB is scarring following blistering, both in the skin and in a variety of mucosae.^6^

Patients with DEB present more oral manifestations as a consequence of mucosal fragility and scaring than patients with the previously described types. A comparative study published in 1992 (based on clinical diagnosis only) compared microstomia (limited mouth opening) and lingual adhesions (ankyloglossia) in a cohort of 197 patients with EB, identifying both features only in individuals affected with DEB.^73^

#### Dominant DEB (DDEB)

##### Soft tissue involvement

**IMAGE 2.6 and 2.7:**
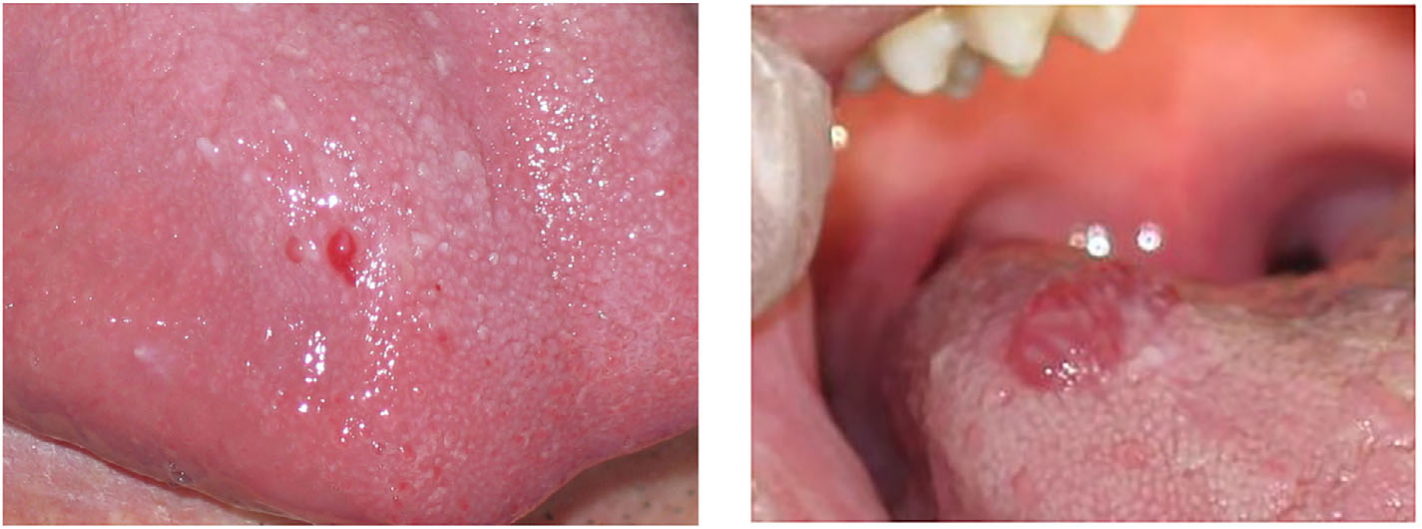
Blood filled bullae on the tongue in patients with DDEB

There is no agreement about the extent of oral mucosal involvement in DDEB. One review stated that 20% of patients have oral mucosal bullae,^11^ while another case series indicated that 71.1% to 89.6% of patients may have a history of or oral clinical features of oral mucosal blistering (Images 2.6 and 2.7).^3^, ^26^ Single case reports vary from no mucosal involvement at all^74^ to frequent intraoral bullae as a result of minor trauma,^75^ painful oral ulcers, severe gingival inflammation, erosive lesions in vestibular region, and restricted mouth opening (microstomia, no measurement provided).^76^ Of note, significant scarring, vestibular obliteration, and ankyloglossia do not seem to be long‐term complications of oral mucosal ulceration/blisters.^3^, ^76^ However, the reduction and absence of keratinized gingiva has been described.^13^, ^75^

##### Hard tissue involvement

Patients with DDEB do not seem to be at increased risk of caries.^2^, ^26^

#### Recessive DEB (RDEB)

The current classification scheme (2020) recognizes six subtypes of RDEB: severe, intermediate, inversa, localized, pruriginosa, and self‐improving RDEB.^6^ The severe subtype presents the more extensive oral manifestations. As the classification scheme of EB has been updated four times in the last 20 years, and the literature describing oral features precedes the current scheme, there is some overlap of clinical descriptions.

##### Generalized forms of RDEB

The following text includes patients with severe RDEB (previously RDEB generalized severe, Hallopeau‐Siemens RDEB) and intermediate RDEB (previously known as RDEB generalized intermediate, non‐Hallopeau‐Siemens RDEB).

##### Perioral tissue involvement

###### Microstomia (mouth opening)

**IMAGE 2.8:**
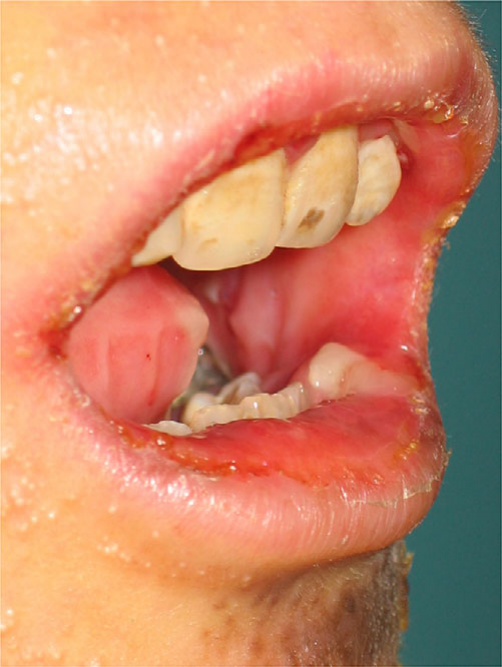
Limited mouth opening in RDEB

Progressive^26^, ^77^ microstomia affects almost all patients with generalized RDEB (Image 2.8).^2^, ^3^, ^24^, ^26^, ^27^, ^78^, ^79^, ^80^, ^81^, ^82^, ^83^, ^84^, ^85^, ^86^, ^87^, ^88^, ^89^, ^90^, ^91^, ^92^, ^93^, ^94^, ^95^, ^96^, ^97^, ^98^, ^99^, ^100^, ^101^, ^102^, ^103^, ^104^, ^105^, ^106^, ^107^, ^108^, ^109^, ^110^, ^111^, ^112^, ^113^, ^114^, ^115^, ^116^, ^117^ Microstomia is not unique to generalized RDEB, and it might also be present in inversa RDEB and severe JEB.^2^, ^26^ The degree of microstomia of patients with severe RDEB has been reported to be severe in over 80% of affected individuals.^13^, ^82^, ^85^, ^87^, ^92^, ^93^, ^94^, ^115^, ^117^ Different techniques to measure microstomia have been used,^3^, ^24^, ^82^, ^91^, ^118^ therefore comparing the results is not feasible.

The precise cause of microstomia in severe RDEB is not clear, although it seems to reflect scarring of the buccal and labial mucosa and commissures.^3^, ^26^, ^111^, ^115^, ^119^ In several patients, fibrous scar bands can be palpated bilaterally at the commissures^113^ and the buccal mucosa.^95^ Microstomia can give rise to a wide variety of functional problems, including difficulties in eating, speech, and oral hygiene maintenance. Furthermore, dental treatment and general anesthesia can be complicated, and the aesthetics of the lower face is compromised.^2^, ^82^, ^84^, ^120^, ^121^

##### Intraoral soft tissue involvement

###### Oral ulcers and blister

**IMAGE 2.9:**
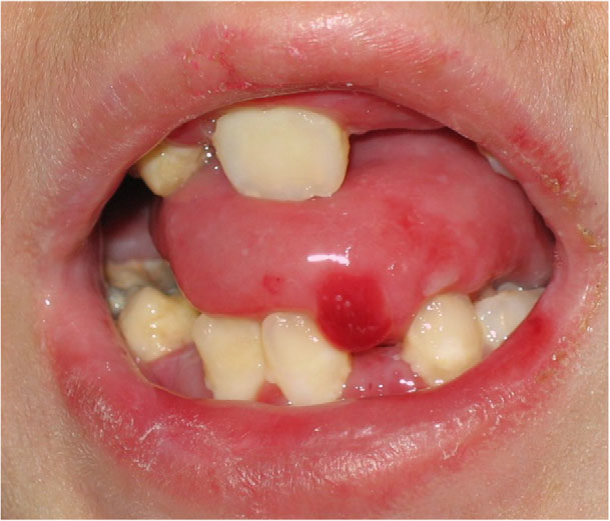
Blood filled bullae on the tongue of a patient with RDEB

The oral mucosa of patients with generalized RDEB is extremely friable and may slough off easily when touched.^86^, ^116^ Recurrent oral mucosal blistering is common, affecting almost all patients^78^, ^80^, ^82^, ^83^, ^84^, ^87^, ^89^, ^92^, ^95^, ^96^, ^97^, ^98^, ^99^, ^101^, ^102^, ^103^, ^106^, ^107^, ^110^, ^111^, ^113^, ^114^, ^119^, ^122^, ^123^, ^124^, ^125^, ^126^ The blisters may be fluid‐ or blood‐filled and arise at any oral mucosal surface, especially the tongue (Images 2.9–2.12).^82^, ^91^, ^102^, ^106^ Some lesions can be caused by sharp edges of broken teeth or restorations.^92^, ^105^ Patients may not allow clinicians to touch their oral mucosa afraid of producing new wounds and causing pain.^100^, ^107^ Others may be afraid of brushing their teeth due to painful blisters in their mouth.^126^ In newborns, these erosions can make oral feeding very challenging, requiring special feeding bottles.^127^ Older patients may be able to tolerate a normal diet but frequent occurrence of oral ulcers and dysphagia can limit their oral intake making them resort to liquid diet.^110^

**IMAGE 2.10:**
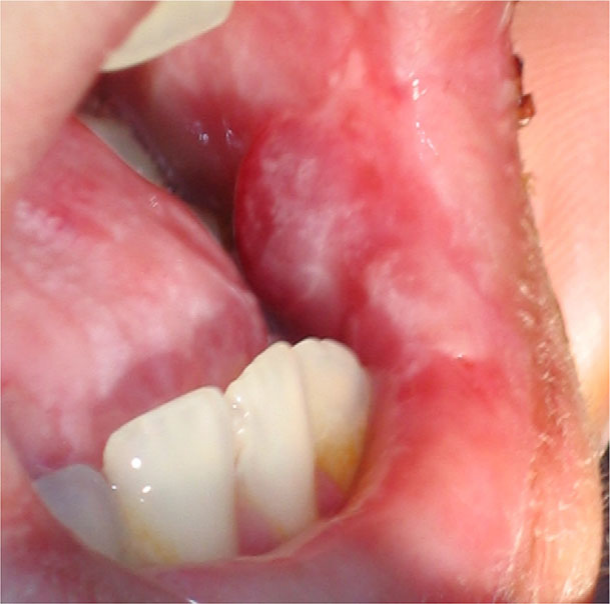
Bullae on the buccal mucosa of a patient with RDEB

**IMAGE 2.11:**
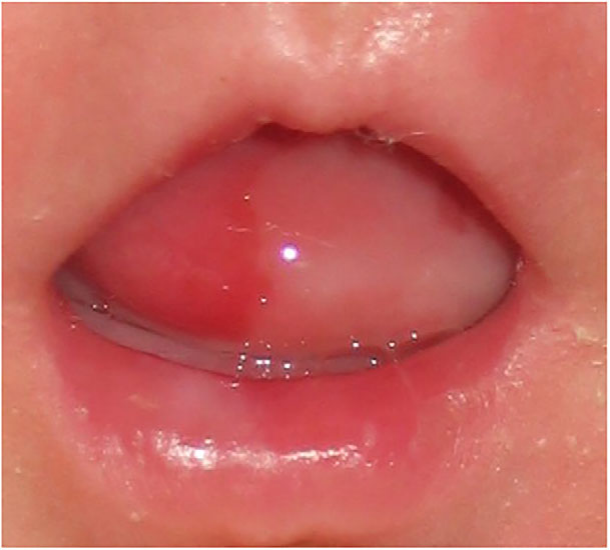
Serous bullae covering 3/5 of the tongue of a newborn with RDEB

**IMAGE 2.12:**
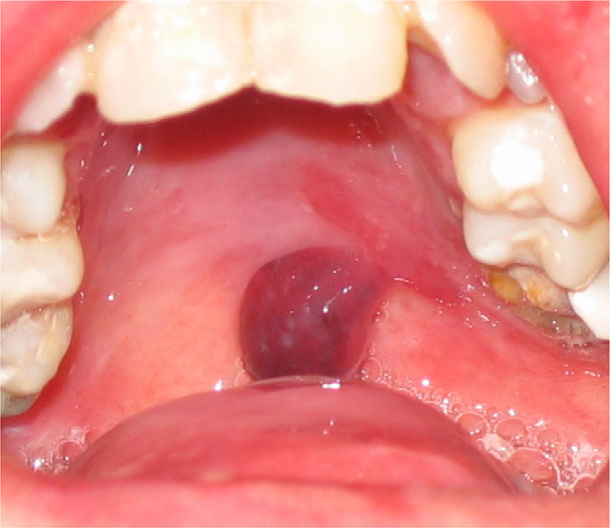
Blood filled bullae on the palate of a patient with RDEB

###### Absence of tongue papillae (depapillated tongue, denuded tongue)

**IMAGE 2.13:**
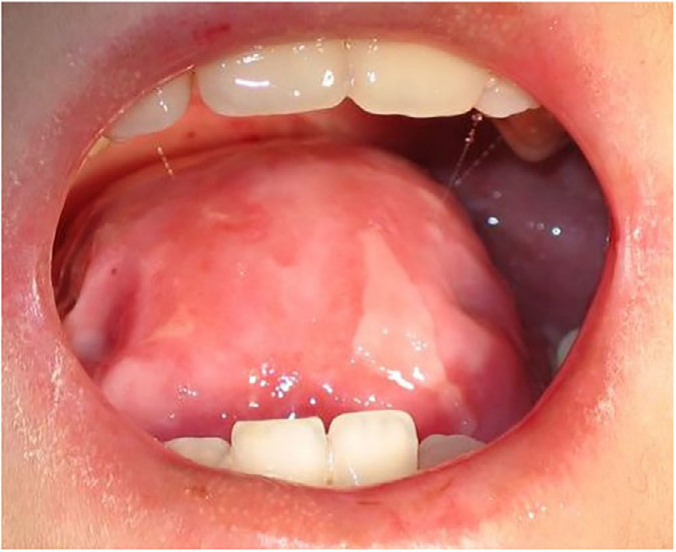
Absence of tongue papillae in RDEB

Tongue papillae are absent. This is often referred to as complete depapillation (Image 2.13).^3^, ^26^, ^27^, ^81^, ^82^, ^84^, ^85^, ^91^, ^95^, ^103^, ^106^, ^108^, ^110^, ^111^, ^113^, ^114^, ^117^, ^119^, ^122^, ^125^

###### Absence of palatal rugae

The absence of palatal rugae has also been described in patients with generalized forms of RDEB.^95^, ^103^

###### Ankyloglossia

**IMAGE 2.14:**
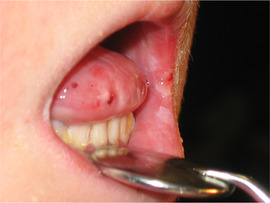
Ankyloglossia in RDEB

Ankyloglossia presumably secondary to ulceration and scarring is common, and indeed may affect all patients (Image 2.14).^2^, ^3^, ^26^, ^27^, ^79^, ^80^, ^81^, ^82^, ^88^, ^91^, ^92^, ^93^, ^94^, ^95^, ^99^, ^101^, ^105^, ^106^, ^108^, ^110^, ^114^, ^115^, ^117^, ^124^, ^128^ A study on oral functions revealed that only 7 of 10 patients with RDEB could stick their tongue forward, with an average of extending the tongue only 6 mm beyond the teeth. In the same study, only 2 of 9 patients could put the tip of the tongue on the left cheek and 1 of 8 on the right side.^24^ This severe ankyloglossia contributes to the feeding difficulties of newborns, requiring a special bottle to feed adequately.^128^

###### Oral vestibule obliteration

**IMAGE 2.15:**
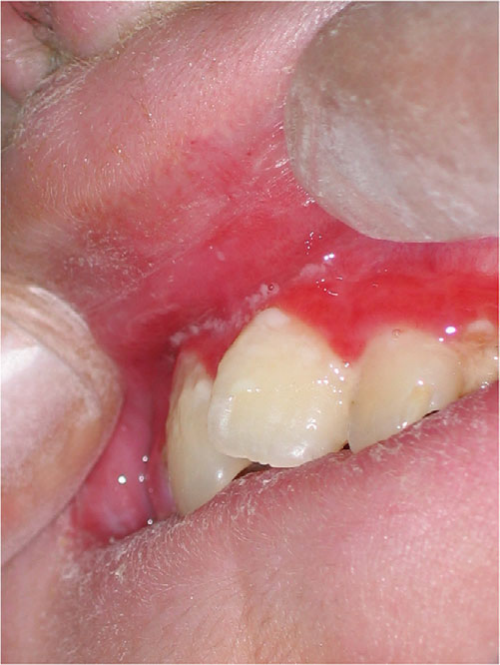
Obliteration of the labial vestibule in RDEB

The scarring in generalized forms of RDEB can give rise to obliteration of the labial and buccal vestibules,^2^, ^3^, ^27^, ^78^, ^79^, ^81^, ^82^, ^83^, ^84^, ^85^, ^89^, ^91^, ^92^, ^93^, ^94^, ^95^, ^101^, ^103^, ^105^, ^108^, ^112^, ^117^, ^119^, ^124^ and hence, has the potential to compromise oral hygiene, dental treatment, and the wearing of removable prosthetic appliances (Image 2.15).

###### Cancer risk

SCC has been described as the leading cause of death in patients with severe RDEB.^129^ Few cases affecting the oral cavity have been reported. The tongue is the most affected site, although tumors on the lip and the hard palate have also been reported. The age of diagnosis ranged from 25 to 54 years of age. At least three cases have been fatal.^3^, ^26^, ^88^, ^130^, ^131^ Of note, Oral SCC has also been described in recessive EBS^9^ and Kindler EB.^132^, ^133^, ^134^, ^135^, ^136^, ^137^, ^138^

###### Periodontal disease

Extensive plaque deposits have been reported on the teeth of most patients.^27^, ^78^, ^80^, ^83^, ^85^, ^86^, ^98^, ^102^, ^106^, ^110^, ^117^, ^126^, ^139^

**IMAGE 2.16:**
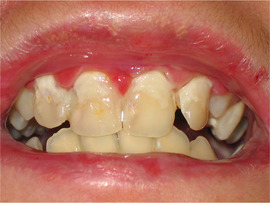
Extensive plaque deposits and gingival inflammation in RDEB

Mean plaque score measured using a modification of the index of O'Leary^140^ revealed higher values for patients with DEB (*n* = 23; 18 RDEB, 5 DDBE) in the primary (33.7 ± 31.3) and secondary dentitions (28.6 ± 31.6) when compared to a control group (1.8 ± 3.3/4.6 ± 5.6, respectively) (Image 2.16).^141^

Gingivitis or gingival inflammation is often reported.^98^, ^105^, ^106^, ^110^, ^125^ Mean gingivitis scores (using the simplified gingival index) have been found to be significantly greater in patients with DEB (*n* = 23; 18 RDEB, 5 DDEB) in both primary (21.5 ± 29) and permanent dentitions (27.5 ± 34.9) when compared to a control group (0.00/2 ± 4.6, respectively).^141^ Fortuna, in 2015, found that erythema was the most prevalent gingival lesion (66.2%) in severe generalized RDEB.^142^ There does not appear to be an increased risk of periodontal membrane and bone involvement in RDEB.^83^, ^84^ Puliyel, in 2014, found deep pockets on periodontal charting. It was explained, however, that they corresponded to pseudopockets, primarily on posterior teeth. Gingival inflammation and bleeding were found on all teeth. Plaque and calculus accumulation were heavy, especially in the lingual and buccal surface of mandibular posterior teeth. Gingival recessions were absent.^95^ Only Al‐Abadi has reported increased mobility and alveolar bone loss around lower anterior teeth.^108^ A retrospective study on dental implants published by Peñarrocha in 2020 demonstrated a success rate of 97.5%. Even though 50% of the implants showed mucositis and bleeding upon probing was observed in 67.5% of the implants, probing depth was maintained at 1‐3 mm in 96.2% of the implants and 52.5% of the implants showed 0 mm retraction of the peri‐implant mucosa after a mean follow‐up of 7.5 years. Keratinized mucosa in the buccal zone of the implants was noted in 62% of the cases, while 38% showed no keratinized, mobile peri‐implant soft tissue. Peri‐implant bone loss after 7.7 years of follow‐up was only 1.65 ± 0.54 mm.^139^

###### Saliva

A study conducted by Leal and coworkers in 2016 compared mucosa hydration, salivary flow, pH, and buffer capacity of individuals with EB to a control group, finding no significant difference between the groups.^118^ Research conducted by Wright found no changes in salivary flow rate. In that cohort of patients with RDEB, significantly elevated salivary IgA, albumin, and total protein levels were noted; most likely related to the high prevalence of oral blistering. They found no evidence to support an association between salivary function and dental caries.^143^

##### Hard tissue involvement

###### Caries

**IMAGE 2.17 and 2.18:**
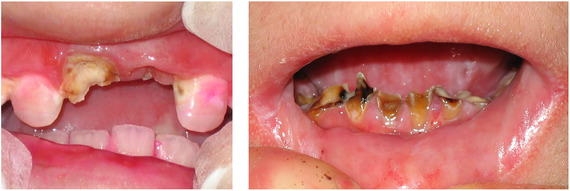
Severe caries in a 12‐year‐old and a 20 years old patient with RDEB

Patients with RDEB have significantly higher caries scores (decay‐missing‐filled [DMF]) index than control patients (Images 2.17 and 2.18).^2^, ^26^, ^79^, ^92^, ^118^, ^141^ Single‐case reports also often highlight increased presence of decayed teeth.^96^, ^97^, ^98^, ^99^, ^102^, ^103^, ^105^, ^106^, ^107^, ^108^, ^110^, ^111^, ^116^, ^125^, ^126^ Some patients have been reported to have pain,^96^, ^105^, ^108^ abscess,^91^, ^96^, ^108^, ^126^ and/or cellulitis secondary to periapical infection,^96^, ^122^ while other patients have lost their entire dentition due to caries,^100^, ^104^, ^111^ presenting very small edentulous ridges.^100^

Risk factors associated with this elevated caries index include: soft diet, limited mouth opening, and contracture of the fingers causing difficulty in maintaining oral hygiene.^118^ It has been noted that extensive caries can be found specifically on the lingual surface.^95^ A study on the mineral, carbonate, protein content, and amino acid composition of the enamel of teeth from patients with RDEB showed normal chemistry.^57^

###### Occlusal abnormalities

A variety of occlusal anomalies have been described in RDEB including increased overjet, overbite,^82^ severe crowding,^3^, ^79^, ^82^, ^91^, ^92^, ^107^ cross‐bite molar relationship,^79^, ^102^ and class II skeletal malocclusion.^91^, ^102^ Some of the anomalies may be due to reduced alveolar arches (secondary to growth retardation) and collapse of the dental arches (secondary to soft tissue constriction).^144^ A cephalometric study of 42 patients with RDEB found significantly smaller jaws in this patient cohort,^145^ thus adding weight to the suggestion that significant dentoalveolar disproportion and dental crowding are features of RDEB.

###### Dental maturity and agenesis

Two studies have been published on dental maturity and dental development in patients with RDEB finding no significant delay.^146^, ^147^ Single‐tooth agenesis has been reported in three cases.^85^, ^113^, ^148^ It is not possible to establish if the incidence is different to the general population.

###### Facial growth

A cephalometric analysis of 42 patients with severe RDEB indicated that this subtype of EB gives rise to a significantly reduced maxillary length, mandibular length, middle facial height, and lower facial height when compared to the published normal values. Saddle and nasolabial angles are significantly greater in RDEB.^145^ The changes in facial skeleton may reflect reduced nutritional intake (feeding problems) and subsequently reduced bone growth.^145^ Additionally, or alternatively, peri‐oral soft tissue scarring during early childhood may result in reduced size of the jaws.^149^

###### Bone health/osteoporosis

Osteoporosis has been increasingly identified in patients with this form of RDEB.^150^ In one report, radiographic records and computerized tomography scans of the jaw revealed extensive bone atrophy of the jaws in six out of six patients.^94^ During surgery, the alveolar ridges of these patients were found to be atrophic in all cases.^93^, ^94^

###### Less frequent findings

A sialolith measuring 8 mm × 7 mm was reported in the submandibular gland of a 17‐year‐old female with RDEB. The removal was challenging due to her microstomia and ankyloglossia.^151^

##### Inversa RDEB

RDEB inversa subtype is an uncommon form of EB. Patients present with mucosal blistering (especially sublingually), ankyloglossia, absence of tongue papillae, absence of palatal rugae, partial obliteration of the vestibule, microstomia secondary to scarring, and mucosal milia.^26^, ^97^, ^152^, ^153^, ^154^ Of note esophageal involvement and dysphagia affected 90% of one group of 10 patients.^152^

##### Hard tissue involvement

A significantly higher prevalence of caries (decayed, missing, and filled surfaces index: DMFS 50.9) than the control group (DMFS: 12.8) was reported in a study of 10 patients. Enamel abnormalities have only been reported in 1 of 14 patients having a localized enamel defect of one tooth.^152^

### 2.4 Kindler EB (KEB) (previously Kindler syndrome)

##### Peri‐oral tissue involvement

Perioral areas can present with erosions, crusts, and chronic cheilitis.^132^, ^133^, ^135^, ^138^, ^155^, ^156^, ^157^, ^158^, ^159^, ^160^, ^161^, ^162^, ^163^, ^164^ Glandular cheilitis of the lower lip have been reported in a 7‐year‐old patient.^159^

##### Microstomia

**IMAGE 2.19:**
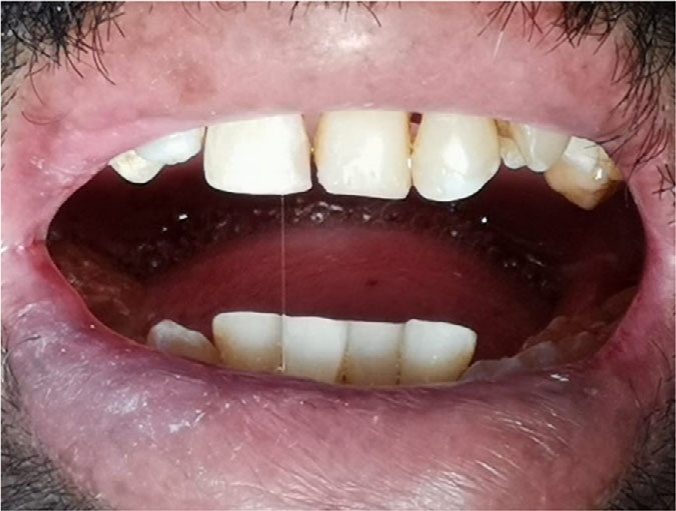
Severe microstomia in a 33‐year‐old patient with Kindler EB

Microstomia, or restricted mouth opening, probably due to fibrosis of the commissures, has been reported in several patients with KEB (Image 2.19).^4^, ^132^, ^135^, ^138^, ^155^, ^156^, ^165^, ^166^, ^167^, ^168^, ^169^, ^170^, ^171^, ^172^ However, many patients do not complain of this functional problem. No studies have been identified on the severity or prevalence of this feature.

##### Intraoral soft tissue involvement

While some patients, particularly younger cases, do not report involvement of the oral mucosa,^173^, ^174^, ^175^, ^176^ others report few ulcers^164^, ^177^, ^178^ and a third group presents fragile mucosa and painful ulcers scattered throughout the oral mucosa, affecting the alveolar ridge, soft palate, lip, and floor of the mouth.^133^, ^162^, ^164^, ^165^, ^179^, ^180^

##### Oral vestibule obliteration

**IMAGE 2.20:**
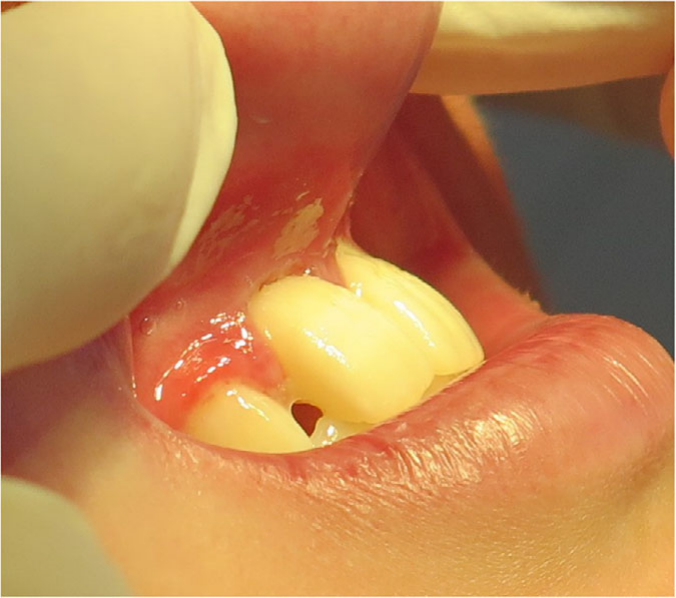
Obliteration of the labial vestibule in a 15‐year‐old patient with Kindler EB

Partial obliteration of the oral vestibule, also described as: “synechiae between the lips and the gums,” “adhesions between the lips and gingiva,” or “atrophy of the buccal mucosa” has been described in several patients with KEB (Image 2.20).^4^, ^132^, ^133^, ^138^, ^155^, ^161^, ^166^, ^167^, ^169^, ^170^, ^171^, ^172^, ^181^, ^182^, ^183^

Reticular pigmentation on the cheek,^160^, ^163^ white hyperkeratotic papules on the buccal mucosa,^165^, ^184^ and xerostomia^160^ are least frequent findings. Penagos in 2004 only found leukokeratosis of the oral mucosa in 3 of 26 patients, supporting the statement that this finding is rather uncommon.^185^

##### Periodontal disease

**IMAGE 2.21:**
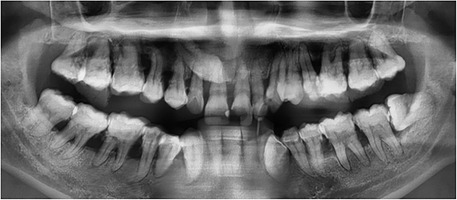
Severe periodontitis in a 33‐year‐old patient with Kindler EB

**IMAGE 2.22:**
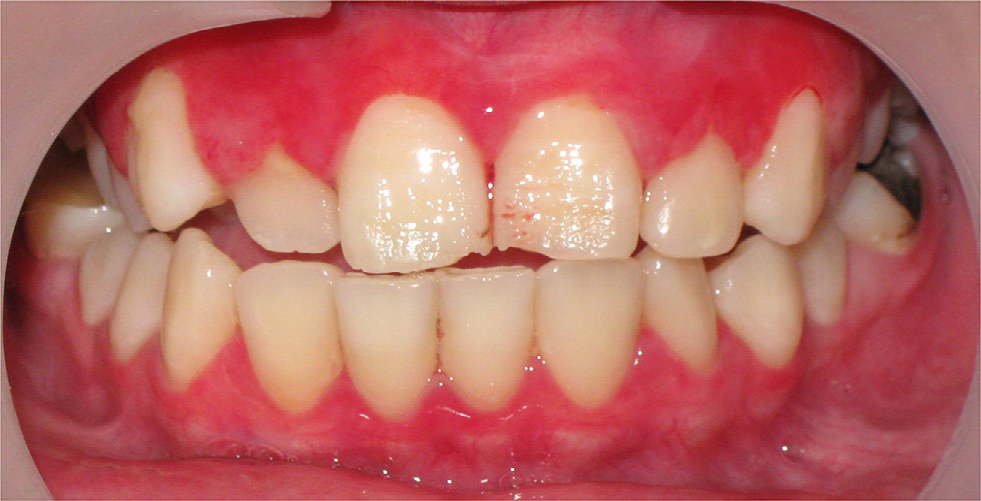
Generalized gingival inflammation in a 13‐year‐old patient with Kindler EB

Special attention has been given to periodontal disease, which was initially reported in two patients.^155^, ^169^ Thereafter a series of 18 patients was compared to healthy controls, and revealed that patients with KEB have a higher prevalence (72% vs. 46%), earlier onset and faster progression of periodontitis (Image 2.21).^4^ The same cohort was followed up and published with 26 individuals, of those 81% developed severe periodontitis with premature loss of teeth.^185^ Periodontitis, tooth loss, mucosal involvement such as microstomia, and caries have been reported in all other case series as well.^186^, ^187^, ^188^ Multiple single‐patient reports also highlight gingival health as a major concern,^132^, ^133^ including a 14‐year‐old patient who lost all of her teeth due to severe periodontitis.^161^ Most of the descriptions of periodontal disease in patients with KEB have been published by medical teams; therefore, the authors use more general terms such as: halitosis,^164^, ^177^ gingivitis,^156^, ^160^, ^163^, ^164^, ^180^, ^181^, ^182^, ^183^, ^189^, ^190^, ^191^ severe gingivitis,^164^, ^168^, ^192^, ^193^ erosive stomatitis,^194^ desquamative gingivitis,^155^, ^160^, ^165^ easy bleeding,^155^, ^157^, ^164^, ^167^, ^180^, ^182^, ^195^, ^196^ gingival hypertrophy,^162^, ^178^, ^197^ periodontitis,^135^, ^157^, ^158^, ^159^, ^162^, ^164^, ^178^, ^180^, ^184^, ^190^, ^198^ ^199^, ^200^, ^201^ severe periodontitis,^161^, ^165^, ^173^, ^192^, ^195^, ^196^ severe periodontal bone loss,^155^ “missing teeth,” “loss of teeth,” or “poor preservation of teeth,”^135^, ^158^, ^160^, ^161^, ^167^, ^168^, ^173^, ^184^, ^190^, ^195^, ^196^, ^200^, ^202^ and gingiva with pseudomembranous necrotic and bleeding areas.^173^ Interestingly, and in contrast to RDEB where poor hygiene is widely reported, only few reports describe poor dental hygiene; even though the severity of periodontal disease in KEB is more complex (Image 2.22).^168^, ^180^

Reports written by dentists, and more specifically periodontists, provide more insight into the periodontal aspects of Kindler EB. A 16‐year‐old patient with KEB presented advanced early‐onset periodontal disease. Her gingiva bled excessively during toothbrushing and even spontaneously. All her deciduous teeth exfoliated between 4 and 7 years of age and the permanent right mandibular central incisor exfoliated spontaneously when she was 10 years old. After oral examination, the diagnosis of periodontitis associated with systemic disease was established.^203^ Another series of five related patients aged 6 to 14 included clinical features, periodontal charts, and radiographs. Microstomia was identified in all patients. The 14‐year‐old patient had his two lower first molars extracted due to bone loss and gingival recession. Panoramic radiograph revealed discrete alveolar bone resorption in all four patients aged 6 to 12 years old and severe alveolar bone resorption in the 14 year old. Periodontal examination showed severe gingival inflammation, with periodontal pockets (>4 mm) in the patients aged 6 to 10 and aggressive periodontitis in the 12‐ and 14‐year‐old patients, with deep pockets (≥7 mm). The gingiva was thin and fragile, and the epithelium sloughed with minor abrasion. With periodontal treatment and regular dental care focused on good oral hygiene periodontal health may be improved and alveolar bone loss can be reduced. Regular dental visits are therefore very important to control desquamative gingivitis and aggressive periodontitis in patients with KEB.^204^

##### Caries

Caries have only been mentioned in two reports.^159^, ^183^ There is no suggestion that patients with Kindler EB would have a prevalence of caries different than the healthy population. Of interest, oral rehabilitation using dental implants have been reported once, with failure after 6 years.^160^

##### Cancer risk

Oral SCC can also present in patients with Kindler EB. To date, at least seven cases of oral SSC have been reported,^132^, ^133^, ^134^, ^135^, ^136^, ^137^, ^138^ two being fatal.^133^, ^135^ The age of diagnosis has ranged from 34 to 55 years of age and the affected sites include hard palate (one extending to the right cheek),^132^, ^135^ buccal mucosa,^134^ upper lip,^137^, ^138^ and lower lip.^133^, ^136^

### Discussion

The evidence gathered in this systematic review demonstrates that the oral findings vary according to the type and subtype of EB (Table 2.1). While patients with JEB, for example, present with generalized enamel hypoplasia as the main clinical challenge, individuals with RDEB present with extremely friable mucosa and severe scaring consequences and those with Kindler EB have early‐onset periodontal disease. This is very important, as it helps to plan every patient's treatment considering their unique features. As EB is the umbrella term for the condition, knowing the precise type and subtype of the condition is of upmost importance for the dental clinician to plan the long‐term care of each patient. Pediatric dentists will lead the team for young patients and special care dentists will lead the team taking care of adults. The information gathered in this article will help guide the inclusion of different dental specialists, for example including oral rehabilitation specialist in teams looking after patients with junctional EB, orthodontists in teams looking after children with RDEB and periodontist for teams caring for patients with Kindler EB.

A major limitation of this review is the lack of precise information on the diagnostic laboratory tests performed on several of the case reports. For example, often the clinical diagnosis is described, but there are no IFM or mutation analysis results to support it. On the other hand, some reports include mutation analysis (eg, mutation in gen *COL7A1)* but do not specify the clinical subtype of DEB the patient lives with. To overcome these limitations, dentists are encouraged to report their clinical cases with precise diagnostic information, including all the laboratory tests to support the diagnosis, as well as considering the latest classification scheme of EB.

A second difficulty is the lack of standardized assessment forms to evaluate oral features unique for EB such as vestibule obliteration, ankyloglossia, microstomia, absence of tongue papillae, and palatal rugae. To date there is the EB oropharyngeal severity score;^205^ however, that score assesses the severity of the condition, and does not describe the clinical features. A standardized EB oral assessment form would be of great benefit for the EB community.

This systematic review highlights the importance of multidisciplinary care. Both dental and medical teams need to communicate better on the precise diagnostic aspects of both the skin and oral conditions.

### Conclusions

Oral manifestations of inherited EB have unique patterns of involvement associated with each subtype of the condition. Understanding each subtype individually will help clinicians when planning initial and long‐term care of those individuals. Furthermore, in a rare disorder with multiple comorbidities, it is vital to treat each patient with EB using a multidisciplinary care approach with clear regular communication between health care professionals involved in the patient's care.

REFERENCES1

Kramer
SM
, 
Serrano
MC
, 
Zillmann
G
, et al. Oral health care for patients with epidermolysis bullosa‐best clinical practice guidelines. Int J Paediatr Dent. 2012;22(Suppl 1):1‐35.2293790810.1111/j.1365-263X.2012.01247.x2

Wright
JT
, 
Fine
JD
, 
Johnson
L
. Dental caries risk in hereditary epidermolysis bullosa. Pediatr Dent. 1994;16(6):427‐432.78549503

Wright
JT
, 
Fine
JD
, 
Johnson
LB
. Oral soft tissues in hereditary epidermolysis bullosa. Oral Surg Oral Med Oral Pathol. 1991;71(4):440‐446.205232910.1016/0030-4220(91)90426-d4

Wiebe
CB
, 
Penagos
H
, 
Luong
N
, et al. Clinical and microbiologic study of periodontitis associated with Kindler syndrome. J Periodontol. 2003;74(1):25‐31.1259359210.1902/jop.2003.74.1.255

Ergun
GA
, 
Lin
AN
, 
Dannenberg
AJ
, 
Carter
DM
. Gastrointestinal manifestations of epidermolysis bullosa. A study of 101 patients. Medicine (Baltimore). 1992;71(3):121‐127.163543710.1097/00005792-199205000-000026

Has
C
, 
Bauer
JW
, 
Bodemer
C
, et al. Consensus reclassification of inherited epidermolysis bullosa and other disorders with skin fragility. Br J Dermatol. 2020;bjd.18921. 10.1111/bjd.18921.320170157

Ozgur
F
, 
Sonmez
E
, 
Tuncbilek
G
. Cleft lip and cleft palate closure in 13 month‐old female with epidermolysis bullosa. J Craniofac Surg. 2005;16(5):843‐847.1619286610.1097/01.scs.0000168768.40862.c58

Touraine
MA
. Classification des epidermolyses bulleuses. Ann Dermatol Syph. 1942;8:138‐144.9

Baek
JO
, 
Lee
HY
, 
Oh
SW
, et al. A novel homozygous keratin 14 mutation in a patient with autosomal recessive epidermolysis bullosa simplex and squamous cell carcinoma of the tongue. Br J Dermatol. 2009;162(4):880‐882.10.1111/j.1365-2133.2009.09614.x2009600810

Ong
WL
, 
Bailey
E
, 
McCormack
C
, et al. Definitive radiotherapy for Merkel cell carcinoma in the setting of epidermolysis bullosa simplex. Australas J Dermatol. 2019;60(2):153‐154.3045056810.1111/ajd.1296011

Sedano
HO
, 
Gorlin
RJ
. Epidermolysis bullosa. Oral Surg Oral Med Oral Pathol. 1989;67(5):555‐563.265479910.1016/0030-4220(89)90272-712

Horn
HM
, 
Tidman
MJ
. The clinical spectrum of epidermolysis bullosa simplex. Br J Dermatol. 2000;142(3):468‐472.1073595210.1046/j.1365-2133.2000.03358.x13

Krämer
SM
. Análisis de las características bucodentarias de pacientes con diferentes subtipos de epidermolisis bullosa. Universidad de Chile; 2006.14

Chuang
LC
, 
Hsu
CL
, 
Lin
SY
. A fixed denture for a child with epidermolysis bullosa simplex. Eur J Paediatr Dent. 2015;16(4):315‐318.2663725715

Lin
Z
, 
Li
S
, 
Feng
C
, et al. Stabilizing mutations of KLHL24 ubiquitin ligase cause loss of keratin 14 and human skin fragility. Nat Genet. 2016;48(12):1508‐1516.2779862610.1038/ng.370116

Lee
JYW
, 
Liu
L
, 
Hsu
C
, et al. Mutations in KLHL24 add to the molecular heterogeneity of epidermolysis bullosa simplex. J Invest Dermatol. 2017;137(5):S88‐S88.10.1016/j.jid.2017.01.0042811112817

Argyropoulou
Z
, 
Liu
L
, 
Ozoemena
L
, et al. A novel PLEC nonsense homozygous mutation (c.7159G >T; p.Glu2387*) causes epidermolysis bullosa simplex with muscular dystrophy and diffuse alopecia: a case report. BMC Dermatol. 2018;18(1):1.2935280910.1186/s12895-018-0069-xPMC577559818

Lee
I
, 
Tian
C
, 
Hurst
A
. Novel heterozygous mutations in PLEC gene causing epidermolysis bullosa simplex with muscular dystrophy, case series of two affected sisters. Muscle and Nerve. 2017;56(3):603.19

Lalor
L
, 
Titeux
M
, 
Palisson
F
, et al. Epidermolysis bullosa simplex–generalized severe type due to keratin 5 p.Glu477Lys mutation: Genotype‐phenotype correlation and in silico modeling analysis. Pediatr Dermatol. 2019;36(1):132‐138.3051586610.1111/pde.1372220

Tryon
RK
, 
Tolar
J
, 
Preusser
SM
, et al. A homozygous frameshift variant in the KRT5 gene is compatible with life and results in severe recessive epidermolysis bullosa simplex. JAAD Case Reports. 2019;5(7):576‐579.3131270510.1016/j.jdcr.2019.03.025PMC661064121

Vahidnezhad
H
, 
Youssefian
L
, 
Daneshpazhooh
M
, et al. Biallelic KRT5 mutations in autosomal recessive epidermolysis bullosa simplex, including a complete human keratin 5 “knock‐out.” Matrix Biol. 2019;83:48‐59.3130224510.1016/j.matbio.2019.07.00222

Abasq‐Thomas
C
, 
Huguen
J
, 
Fraitag
S
, 
Misery
L
. Kaposi varicelliform eruption in a patient with dowling‐meara epidermolysis bullosa. Pediatr Dermatol. 2016;33:S28.10.1016/j.jdcr.2016.03.008PMC48875842728457123

Johar
U
, 
Jain
D
, 
Edge
CJ
. Neonatal teeth associated with epidermolysis bullosa: a case report. Br J Oral Maxillofac Surg. 2014;52(8):e116.24

Stellingsma
C
, 
Dijkstra
PU
, 
Dijkstra
J
, et al. Restrictions in oral functions caused by oral manifestations of epidermolysis bullosa. Eur J Dermatology. 2011;21(3):405–409.10.1684/ejd.2011.13562160990025

Carroll
DL
, 
Stephan
MJ
, 
Hays
GL
. Epidermolysis bullosa–review and report of case. J Am Dent Assoc. 1983;107(5):749‐751.622764810.14219/jada.archive.1983.032226

Wright
JT
. Oral Manifestations of Epidermolysis Bullosa In: Epidermolysis Bullosa Clinical, Epidemiologic, and Laboratory Advances and the Findings of the National Epidermolysis Bullosa Registry. Baltimore: The Johns Hopkins University Press; 1999:236‐256.27

Crawford
EG
, 
Burkes
EJ
, 
Briggaman
RA
. Hereditary epidermolysis bullosa: oral manifestations and dental therapy. Oral Surg Oral Med Oral Pathol. 1976;42(4):490‐500.106754810.1016/0030-4220(76)90296-628

Blom
A
, 
Caux
F
, 
Charlesworth
A
, et al. A new LAMA3 mutation in 2 patients with junctional epidermolysis bullosa. J Invest Dermatol. 2010;130:S81.29

Ungureanu
S
, 
Adni
T
, 
Brown
T
, 
Inston
N
, 
Heagerty
A
. Successful renal transplant in a patient with non‐Herlitz junctional epidermolysis bullosa. Clin Exp Dermatol. 2014;39(3):330‐332.2463507210.1111/ced.1230030

Kiritsi
D
, 
Huilaja
L
, 
Franzke
C‐W
, et al. Junctional epidermolysis bullosa with LAMB3 splice‐site mutations. Acta Derm Venereol. 2015;95(7):849‐851.2570856310.2340/00015555-207331

Wright
JT
. Epidermolysis bullosa: dental and anesthetic management of two cases. Oral Surg Oral Med Oral Pathol. 1984;57(2):155‐157.658362210.1016/0030-4220(84)90204-432

Nakamura
H
, 
Sawamura
D
, 
Goto
M
, et al. Analysis of the COL17A1 in non‐Herlitz junctional epidermolysis bullosa and amelogenesis imperfecta. Int J Mol Med. 2006;18(2):333‐337.1682094333

Yuen
WY
, 
Pasmooij
AMG
, 
Stellingsma
C
, 
Jonkman
MF
. Enamel defects in carriers of a novel LAMA3 mutation underlying epidermolysis bullosa. Acta Derm Venereol. 2012;92(6):695‐696.2243418510.2340/00015555-134134

Hayashi
AH
, 
Galliani
CA
, 
Gillis
DA
. Congenital pyloric atresia and junctional epidermolysis bullosa: A report of long‐term survival and a review of the literature. Journal of Pediatric Surgery. 1991;26:1341‐1345. 10.1016/0022-3468(91)90616-2.181227135

Valaki
MD
, 
Phillips
RJ
, 
Lake
BD
, 
Harper
JI
. Junctional epidermolysis bullosa and pyloric atresia: a distinct entity. Clinical and pathological studies in five patients. Br J Dermatol. 2006;133(5):732‐736.10.1111/j.1365-2133.1995.tb02747.x855502536

Diociaiuti
A
, 
Castiglia
D
, 
Morini
F
, et al. Long‐term follow‐up of a spontaneously improving patient with junctional epidermolysis bullosa associated with ITGB4 c.3977‐19T>a splicing mutation. Acta Derm Venereol. 2013;93(1):116‐118.2267421210.2340/00015555-138137

D'Angelo
M
. [Oral lesions in epidermolysis bullosa]. Minerva Stomatol. 1981;30(3):169‐174.694341238

Putnam
JJ
, 
Sferra
GWJ
, 
Carter
DM
, et al. Dental problems in junctional epidermolysis bullosa: report of a case with treatment considerations. Ann Dent. 1995;54(1‐2):14‐17.857253739

Brooks
JK
, 
Bare
LC
, 
Davidson
J
, 
Taylor
LS
, 
Wright
JT
. Junctional epidermolysis bullosa associated with hypoplastic enamel and pervasive failure of tooth eruption: oral rehabilitation with use of an overdenture. Oral Surg Oral Med Oral Pathol Oral Radiol Endod. 2008;105(4):e24‐e28.1832956410.1016/j.tripleo.2007.12.03840

Sadler
E
, 
Laimer
M
, 
Diem
A
, et al. Zahnveranderung bei junktionaler Epidermolysis bullosa ‐ Bericht uber eine Patientin mit einer Mutation im LAMB3‐Gen. Dental alterations in junctional epidermolysis bullosa ‐ report of a patient with a mutation in the LAMB3‐gene. J der Dtsch Dermatologischen Gesellschaft. 2005;3(5):359‐363.10.1111/j.1610-0387.2005.05703.x1637280341

Arwill
T
, 
Olsson
O
, 
Bergenholtz
A
. Epidermolysis bullosa hereditaria. 3. A histologic study of changes in teeth in the polydysplastic dystrophic and lethal forms. Oral Surg Oral Med Oral Pathol. 1965;19:723‐744.1429856010.1016/0030-4220(65)90342-742

Gardner
DG
, 
Hudson
CD
. The disturbances in odontogenesis in epidermolysis bullosa hereditaria letalis. Oral Surg Oral Med Oral Pathol. 1975;40(4):483‐493.105843910.1016/0030-4220(75)90246-743

Brain
EB
, 
Wigglesworth
JS
. Developing teeth in epidermolysis bullosa hereditaria letalis. A histological study. Br Dent J. 1968;124(6):255‐260.523870144

Lazarus
GS
. Collagenase and connective tissue metabolism in epidermolysis bullosa. J Invest Dermatol. 1972;58(4):242‐248.433652210.1111/1523-1747.ep1253994645

Wright
JT
, 
Cashion
S
, 
Hoover
R
. The esthetic stainless steel crown bridge: Report of two cases. Pediatr Dent. 1999;21(2):137‐141.1019734446

Wright
JT
, 
Johnson
LB
, 
Fine
JD
. Development defects of enamel in humans with hereditary epidermolysis bullosa. Arch Oral Biol. 1993;38(11):945‐955.829725810.1016/0003-9969(93)90107-w47

Bohaty
B
, 
Spencer
P
, 
Dunlap
C
, 
Wandera
A
. Epidermolysis bullosa: case report of appropriate classification of subtype because of an early dental exam. J Clin Pediatr Dent. 1998;22(3):243‐245.964110048

Wright
JT
, 
Hall
KI
, 
Deaton
TG
, 
Fine
JD
. Structural and compositional alteration of tooth enamel in hereditary epidermolysis bullosa. Connect Tissue Res. 1996;34(4):271‐279.908463610.3109/0300820960900527149

Kirkham
J
, 
Robinson
C
, 
Strafford
SM
, et al. The chemical composition of tooth enamel in junctional epidermolysis bullosa. Arch Oral Biol. 2000;45(5):377‐386.1073985910.1016/s0003-9969(00)00003-050

McGrath
JA
, 
Gatalica
B
, 
Li
K
, et al. Compound heterozygosity for a dominant glycine substitution and a recessive internal duplication mutation in the type XVII collagen gene results in junctional epidermolysis bullosa and abnormal dentition. Am J Pathol. 1996;148(6):1787‐1796.8669466PMC186165051

Laimer
M
, 
Nischler
E
. Intraoral disease In: Fine J‐D, Hintner H, eds. Life with Epidermolysis Bullosa (EB): Etiology, Diagnosis, Multidisciplinary Care and Therapy. Vienna: Springer Vienna; 2008:150‐166.52

Bauer
JW
, 
Lanschuetzer
C
. Type XVII collagen gene mutations in junctional epidermolysis bullosa and prospects for gene therapy. Clin Exp Dermatol. 2003;28(1):53‐60.1255863210.1046/j.1365-2230.2003.01192.x53

Jonkman
MF
, 
Pas
HH
, 
Nijenhuis
M
, 
Kloosterhuis
G
, 
van der Steege
G
. Deletion of a cytoplasmic domain of integrin β4 causes epidermolysis bullosa simplex1. J Invest Dermatol. 2002;119(6):1275‐1281.1248542810.1046/j.1523-1747.2002.19609.x54

Olague‐Marchan
M
, 
Twining
SS
, 
Hacker
MK
, et al. A disease‐associated glycine substitution in BP180 (type XVII collagen) leads to a local destabilization of the major collagen triple helix. Matrix Biol. 2000;19(3):223‐233.1093644710.1016/s0945-053x(00)00070-655

Kirkham
J
, 
Robinson
C
, 
Strafford
SM
, et al. The chemical composition of tooth enamel in junctional epidermolysis bullosa. Arch Oral Biol. 2000;45(5):377‐386.1073985910.1016/s0003-9969(00)00003-056

Adya
KA
, 
Inamadar
AC
, 
Palit
A
. “Pitted” lesions in dermatology. Int J Dermatol. 2017;56(1):3‐17.2761360510.1111/ijd.1335857

Kirkham
J
, 
Robinson
C
, 
Strafford
SM
, et al. The chemical composition of tooth enamel in recessive dystrophic epidermolysis bullosa: Significance with respect to dental caries. J Dent Res. 1996;75(9):1672‐1678.895262010.1177/0022034596075009090158

Asaka
T
, 
Akiyama
M
, 
Domon
T
, et al. Type XVII collagen is a key player in tooth enamel formation. Am J Pathol. 2009;174(1):91‐100.1903680610.2353/ajpath.2009.080573PMC263132259

Allegaert
K
, 
Naulaers
G
. Gabapentin as part of multimodal analgesia in a newborn with epidermolysis bullosa. Paediatr Anaesth. 2010;20(10):972‐973.2084951310.1111/j.1460-9592.2010.03396.x60

Parsapour
K
, 
Reep
MD
, 
Mohammed
L
, 
Church
A
, 
Shwayder
T
. Herlitz junctional epidermolysis bullosa presenting at birth with anonychia: a case report and review of H‐JEB. Pediatr Dermatol. 2001;18(3):217‐222.1143800210.1046/j.1525-1470.2001.018003217.x61

Kittridge
A
, 
Patel
R
, 
Novoa
R
, 
Tamburro
J
. Herlitz junctional epidermolysis bullosa with a novel mutation in LAMB3. Pediatr Dermatol. 2014;31(4):530‐532.2327829110.1111/pde.1201862

Cheney‐Peters
D
, 
Lund
TC
. Oral pyogenic granuloma after bone marrow transplant in the pediatric/adolescent population: report of 5 cases. J Pediatr Hematol Oncol. 2016;38(7):570‐573.2727181310.1097/MPH.000000000000059363

Momeni
A
, 
Pieper
K
. Junctional epidermolysis bullosa: a case report. Int J Paediatr Dent. 2005;15(2):146‐150.1579037510.1111/j.1365-263X.2005.00622.x64

Hintner
H
, 
Wolff
K
. Generalized atrophic benign epidermolysis bullosa. Arch Dermatol. 1982;118(6):375‐384.709224965

Pasmooij
AMG
, 
Pas
HH
, 
Jansen
GHL
, 
Lemmink
HH
, 
Jonkman
MF
. Localized and generalized forms of blistering in junctional epidermolysis bullosa due to COL17A1 mutations in the Netherlands. Br J Dermatol. 2007;156(5):861‐870.1726380710.1111/j.1365-2133.2006.07730.x66

Murrell
DF
, 
Pasmooij
AMG
, 
Pas
HH
, et al. Retrospective diagnosis of fatal BP180‐deficient non‐herlitz junctional epidermolysis bullosa suggested by immunofluorescence (IF) antigen‐mapping of parental carriers bearing enamel defects [3]. J Invest Dermatol. 2007;127(7):1772‐1775.1734492710.1038/sj.jid.570076667

Almaani
N
, 
Liu
L
, 
Dopping‐Hepenstal
PJC
, et al. Autosomal dominant junctional epidermolysis bullosa. Br J Dermatol. 2009;160(5):1094‐1097.1912033810.1111/j.1365-2133.2008.08977.x68

Nakar
S
, 
Ingber
A
, 
Kremer
I
, et al. Late‐onset localized junctional epidermolysis bullosa and mental retardation: a distinct autosomal recessive syndrome. Am J Med Genet. 1992;43(5):776‐779.164226010.1002/ajmg.132043050369

Barzegar
M
, 
Mozafari
N
, 
Kariminejad
A
, et al. A new homozygous nonsense mutation in LAMA3A underlying laryngo‐onycho‐cutaneous syndrome. Br J Dermatol. 2013;169(6):1353‐1356.2386944910.1111/bjd.1252270

Phillips
RJ
, 
Atherton
DJ
, 
Gibbs
ML
, 
Strobel
S
, 
Lake
BD
. Laryngo‐onycho‐cutaneous syndrome: an inherited epithelial defect. Arch Dis Child. 1994;70(4):319‐326.818536610.1136/adc.70.4.319PMC102978671

Cohn
HI
, 
Murrell
DF
. Laryngo‐onycho‐cutaneous syndrome. Dermatol Clin. 2010;28(1):89‐92.1994562010.1016/j.det.2009.10.01072

Sarkar
S
, 
Kumar
R
, 
Nandi
M
. A rare skin disorder misdiagnosed as juvenile idiopathic arthritis. Indian J Pediatr. 2016;83(7):742‐743.2655891410.1007/s12098-015-1922-073

Travis
SP
, 
McGrath
JA
, 
Turnbull
AJ
, et al. Oral and gastrointestinal manifestations of epidermolysis bullosa. Lancet (London, England). 1992;340(8834‐8835):1505‐1506.10.1016/0140-6736(92)92759-9136160074
14th Annual Medical Dermatology Meeting. Br J Dermatol. 2019;180(5):e146‐e177.3102572410.1111/bjd.1771875

Brain
JH
, 
Paul
BF
, 
Assad
DA
. Periodontal plastic surgery in a dystrophic epidermolysis bullosa patient: review and case report. J Periodontol. 1999;70(11):1392‐1396.1058850410.1902/jop.1999.70.11.139276

Parushetti
AD
, 
Agrawal
JM
, 
Nanjannawar
LG
, et al. Oral manifestations of epidermolysis bullosa dystrophica: a rare genetic disease. BMJ Case Rep. 2013;2013
10.1136/bcr-2012-007963.PMC36044612334917577

Krämer
SM
. Oral care and dental management for patients with epidermolysis bullosa. Dermatol Clin. 2010;28(2):303‐309.2044749510.1016/j.det.2010.02.02178

Olsen
CB
, 
Bourke
LF
. Recessive dystrophic epidermolysis bullosa. Two case reports with 20‐year follow‐up. Aust Dent J. 1997;42(1):1‐7.907863810.1111/j.1834-7819.1997.tb00087.x79

De Benedittis
M
, 
Petruzzi
M
, 
Favia
G
, 
Serpico
R
. Oro‐dental manifestations in Hallopeau‐Siemens‐type recessive dystrophic epidermolysis bullosa. Clin Exp Dermatol. 2004;29(2):128‐32.1498726510.1111/j.1365-2230.2004.01485.x80

Silva
LCP
, 
Cruz
RA
, 
Abou‐Id
LR
, 
Brini
LNB
, 
Moreira
LS
. Clinical evaluation of patients with epidermolysis bullosa: review of the literature and case reports. Spec Care Dentist. 2004;24(1):22‐27.1515705610.1111/j.1754-4505.2004.tb01675.x81

Siqueira
MA
, 
de Souza Silva
J
, 
Silva
FWG de P
, et al. Dental treatment in a patient with epidermolysis bullosa. Spec Care Dent. 2008;28(3):92‐95.10.1111/j.1754-4505.2008.00012.x1848965582

Serrano Martínez
C
, 
Silvestre Donat
FJ
, 
Bagán Sebastián
JV
, 
Peñarrocha Diago
M
, 
Alió Sanz
JJ
. Epidermólisis ampollosa hereditaria a propósito del manejo odontológico de tres casos clínicos. Med Oral. 2001;6(1):48‐56.1148813183

Harel‐Raviv
M
, 
Bernier
S
, 
Raviv
E
, 
Gornitsky
M
. Oral epidermolysis bullosa in adults. Spec Care Dentist. 1995;15(4):144‐148.900291710.1111/j.1754-4505.1995.tb00502.x84

Kaslick
RS
, 
Brustein
HC
. Epidermolysis bullosa. Review of the literature and report of a case. Oral Surg Oral Med Oral Pathol. 1961;14:1315‐1330.1445415910.1016/0030-4220(61)90263-885

Lindemeyer
R
, 
Wadenya
R
, 
Maxwell
L
. Dental and anaesthetic management of children with dystrophic epidermolysis bullosa. Int J Paediatr Dent. 2009;19(2):127‐134.1925039510.1111/j.1365-263X.2008.00940.x86

Haas
CD
. Epidermolysis bullosa dystrophica. Report of a case. Oral Surg Oral Med Oral Pathol. 1968;26(3):291‐295.524390510.1016/0030-4220(68)90397-687

Serrano‐Martínez
MC
, 
Bagán J
V
, 
Silvestre
FJ
, 
Viguer
MT
. Oral lesions in recessive dystrophic epidermolysis bullosa. Oral Dis. 2003;9(5):264‐268.1462889410.1034/j.1601-0825.2003.03971.x88

Reed
WB
, 
College
J
, 
Francis
MJO
, et al. Epidermolysis bullosa dystrophica with epidermal neoplasms. Arch Dermatol. 1974;110(6):894.461327989

Delebarre
H
, 
Chiaverini
C
, 
Vandersteen
C
, 
Savoldelli
C
. Orofacial management for epidermolysis bullosa during wisdom tooth removal surgery: A technical note. J Stomatol oral Maxillofac Surg. 2019;120(5):467‐470.3091076510.1016/j.jormas.2019.03.00790

Moghadam
BK
, 
Gier
RE
. Epidermolysis bullosa: oral management and case reports. ASDC J Dent Child. 1992;59(1):66‐69.153794591

Serrano Martínez
C
, 
Silvestre Donat
FJ
, 
Bagán Sebastián
JV
, 
Peñarrocha Diago
M
, 
Alió Sanz
JJ
. Hereditary epidermolysis bullosa, dental management of three cases. Med Oral. 2001;6(1):48‐56.1148813192

Korolenkova
MV
. [Dental treatment in children with dystrophic form of epidermolysis bullosa]. Stomatologiia (Mosk). 2015;94(2):34‐36.2614547510.17116/stomat201594234-3693

Peñarrocha‐Diago
M
, 
Serrano
C
, 
Sanchis
JM
, 
Silvestre
FJ
, 
Bagán
JV
. Placement of endosseous implants in patients with oral epidermolysis bullosa. Oral Surg Oral Med Oral Pathol Oral Radiol Endod. 2000;90(5):587‐590.1107738110.1067/moe.2000.11043894

Peñarrocha
M
, 
Larrazábal
C
, 
Balaguer
J
, et al. Restoration with implants in patients with recessive dystrophic epidermolysis bullosa and patient satisfaction with the implant‐supported superstructure. Int J Oral Maxillofac Implants. 2007;22(4):651‐655.1792952895

Puliyel
D
, 
Chiu
C
, 
Habibian
M
. Restorative and periodontal challenges in adults with dystrophic epidermolysis bullosa. J Calif Dent Assoc. 2014;42(5):313‐318.2508734996

Rekka
P
, 
Swathi
S
, 
Prabhu
Very
, 
Ramesh
S
. Dental and anesthetic management of a child with epidermolysis bullosa. J Indian Soc Pedod Prev Dent. 2011;29(2):155.2191195610.4103/0970-4388.8469097

Danescu
S
, 
Has
C
, 
Senila
S
, 
Ungureanu
L
, 
Cesarean
R
. Epidemiology of inherited epidermolysis bullosa in Romania and genotype‐phenotype correlations in patients with dystrophic epidermolysis bullosa. J Eur Acad Dermatol Venereol. 2015;29(5):899‐903.2520108910.1111/jdv.1270998

Mello
BUFF
, 
Net
NL
, 
Kobayashi
TY
, et al. General anesthesia for dental care management of a patient with epidermolysis bullosa: 24‐month follow‐up. Spec Care Dent. 2016;36(4):237‐240.10.1111/scd.121702693663299

Cuadrado‐Corrales
N
, 
Sánchez‐Jimeno
C
, 
García
M
, et al. A recurrent nonsense mutation occurring as a de novo event in a patient with recessive dystrophic epidermolysis bullosa. Dermatology. 2011;223(3):219‐221.2184976910.1159/000330331100

Müller
F
, 
Bergendal
B
, 
Wahlmann
U
, 
Wagner
W
. Implant‐supported fixed dental prostheses in an edentulous patient with dystrophic epidermolysis bullosa. Int J Prosthodont. 2010;23(1):42‐48.20234891101

Oliveira
MA
, 
Ortega
KL
, 
Martins
FM
, 
Maluf
PSZ
, 
Magalhes
MG
. Recessive dystrophic epidermolysis bullosa”oral rehabilitation using stereolithography and immediate endosseous implants. Spec Care Dent. 2010;30(1):23‐26.10.1111/j.1754-4505.2009.00117.x20051071102

Pacheco
W
, 
Marques de Sousa Araugio
R
. Orthodontic treatment of a patient with recessive dystrophic epidermolysis bullosa: a case report. Spec Care Dent. 2008;28(4):136‐139.10.1111/j.1754-4505.2008.00028.x18647373103

Pekiner
FN
, 
Yucelten
D
, 
Ozbayrak
S
, 
Sezen
EC
. Oral‐clinical findings and management of epidermolysis bullosa. J Clin Pediatr Dent. 2005;30(1):59‐65.1630260210.17796/jcpd.30.1.y503845545kn78x7104

Lee
H
, 
Al Mardini
M
, 
Ercoli
C
, 
Smith
MN
. Oral rehabilitation of a completely edentulous epidermolysis bullosa patient with an implant‐supported prosthesis: a clinical report. J Prosthet Dent. 2007;97(2):65‐69.1734137210.1016/j.prosdent.2006.12.010105

Louloudiadis
AK
, 
Louloudiadis
KA
. Case report: dystrophic epidermolysis bullosa: dental management and oral health promotion. Eur Arch Paediatr Dent. 2009;10(1):42‐45.1925452710.1007/BF03262667106

Oliveira
TM
, 
Sakai
VT
, 
Candido
LA
, 
Silva
SMB
, 
Machado
MAAM
. Clinical management for epidermolysis bullosa dystrophica. J Appl Oral Sci. 2008;16(1):81‐85.1908929510.1590/S1678-77572008000100016PMC4327286107

Camm
JH
, 
Gray
SE
, 
Mayes
TC
. Combined medical‐dental treatment of an epidermolysis bullosa patient. Spec Care Dentist. 1991;11(4):148‐150.183517210.1111/j.1754-4505.1991.tb01525.x108

Al‐Abadi
A
, 
Al‐Azri
SA
, 
Bakathir
A
, 
Al‐Riyami
Y
. Dental and anaesthetic challenges in a patient with dystrophic epidermolysis bullosa. Sultan Qaboos Univ Med J. 2016;16(4):e495‐e499.2800389910.18295/squmj.2016.16.04.016PMC5135464109

Djurić
Z
, 
Nagorni
A
, 
Živanović
D
. Esophagitis and almost complete esophageal occlusion in a girl with epidermolysis bullosa. Turk J Pediatr. 2012;54(3):301‐304.23094544110

Reddy
SS
, 
Lanjekar
A
, 
Kaushik
A
. Dystrophic epidermolysis bullosa: report of a case with electron microscopic study. Indian J Dermatol. 2011;56(4):456‐458.2196587210.4103/0019-5154.84718PMC3179027111

Block
MS
, 
Gross
BD
. Epidermolysis bullosa dystrophica recessive: oral surgery and anesthetic considerations. J Oral Maxillofac Surg. 1982;40(11):753‐758.695756610.1016/0278-2391(82)90154-9112

Schauer
F
, 
Hoffmann
J
, 
Fischer
J
, 
Has
C
. Oral manifestations as the main feature of late‐onset recessive dystrophic epidermolysis bullosa. J Eur Acad Dermatol Venereol. 2018;32(4):e161‐e163.2908032110.1111/jdv.14663113

Torres
CP
, 
Gomes‐Silva
JM
, 
Mellara
TS
, 
Carvalho
LP
, 
Borsatto
MC
. Dental care management in a child with recessive dystrophic epidermolysis bullosa. Braz Dent J. 2011;22(6):511‐516.2218964810.1590/s0103-64402011000600012114

Kummer
TR
, 
Nagano
HCM
, 
Tavares
SS
, 
Santos
BZ Dos
, 
Miranda
C
. Oral manifestations and challenges in dental treatment of epidermolysis bullosa dystrophica. J Dent Child (Chic). 2013;80(2):97‐100.24011299115

Stavropoulos
F
, 
Abramowicz
S
. Management of the oral surgery patient diagnosed with epidermolysis bullosa: report of 3 cases and review of the literature. J Oral Maxillofac Surg. 2008;66(3):554‐559.1828039410.1016/j.joms.2007.06.672116

Finke
C
, 
Haas
N
, 
Czarnetzki
BM
. [Value of dental treatment in interdisciplinary management of a child with epidermolysis bullosa dystrophica hereditaria (Hallopeau‐Siemens)]. Hautarzt. 1996;47(4):307‐310.865531810.1007/s001050050421117

Azrak
B
, 
Kaevel
K
, 
Hofmann
L
, 
Gleissner
C
, 
Willershausen
B
. Dystrophic epidermolysis bullosa: oral findings and problems. Spec Care Dentist. 2006;26(3):111‐115.1677418810.1111/j.1754-4505.2006.tb01433.x118

Leal
SC
, 
Lia
EN
, 
Amorim
R
, et al. Higher dental caries prevalence and its association with dietary habits and physical limitation in Epidermolysis Bullosa patients: a case control study. J Contemp Dent Pract. 2016;17(3):211‐216.2720720010.5005/jp-journals-10024-1829119

Boyer
HE
, 
Owens
RH
. Epidermolysis bullosa: a rare disease of dental interest. Review of the literature and report of a case. Oral Surg Oral Med Oral Pathol. 1961;14:1170‐1177.1387218210.1016/0030-4220(61)90205-5120

George
M
, 
Martinez
AE
, 
Mellerio
JE
, 
Nandi
R
. Maxillary alveolar process fracture complicating intubation in a patient with epidermolysis bullosa. Pediatr Anesth. 2009;19(7):706‐707.10.1111/j.1460-9592.2009.02995.x19638122121

Dougherty
ME
, 
Warden
GD
. A thirty‐year review of oral appliances used to manage microstomia, 1972 to 2002. J Burn Care Rehabil. 2003;24(6):418‐431.1461043310.1097/01.BCR.0000095517.97355.98122

Album
MM
, 
Gaisin
A
, 
Lee
KWT
, et al. Epidermolysis bullosa dystrophica polydysplastica: a case of anesthetic management in oral surgery. Oral Surg Oral Med Oral Pathol. 1977;43(6):859‐872.26667910.1016/0030-4220(77)90078-0123

Grover
S
. Generalised recessive dystrophic epidermolysis bullosa in two sisters. Indian J Dermatol Venereol Leprol. 2001;67(4):205‐256.17664745124

Nicopoulou‐Karayianni
E
, 
Patsakas
A
. [Epidermolysis bullosa dystrofica. Report of a case]. Hell Stomatol Chron. 1988;32(2):152‐154.3153693125

Singh‐Rambiritch
S
, 
Mokhine
IMN
, 
Wood
NH
, et al. Oral Medicine Case Book 48: epidermolysis bullosa. SADJ. 2013;68(3):132‐134.23951778126

Morgan
WC.

Dental anesthetic management of epidermolysis bullosa: a new approach. Oral Surg Oral Med Oral Pathol. 1975;40(6):732‐735.106002810.1016/0030-4220(75)90440-5127

Pawlaczyk
M
, 
Jaworska
A
, 
Kornacka
A
, et al. Dystrophic epidermolysis bullosa Hallopeau‐Siemens and localized congenital absence of the skin in the neonate. Przegl Dermatol. 2003;90(6):465‐469.128

McPhie
A
, 
Merkel
K
, 
Lossius
M
, 
Giordano
BP
, 
Kelly
MN
. Newborn infant with epidermolysis bullosa and ankyloglossia. J Pediatr Health Care. 2016;30(4):390‐395.2683321210.1016/j.pedhc.2015.12.005129

Fine
J‐D
. Premature death in inherited epidermolysis bullosa In: FineJ‐D, BauerEA, McGuireJ, MoshellA, eds. Epidermolysis Bullosa Clinical, Epidemiologic, and Laboratory Advances and the Findings of the National Epidermolysis Bullosa Registry. Baltimore: The Johnns Hopkins University Press; 1999:206‐224.130

Martinez
L
, 
Goodman
P
, 
Crow
WN
. Squamous cell carcinoma of the maxillary sinus and palate in epidermolysis bullosa: CT demonstration. J Comput Assist Tomogr. 1992;16(2):317‐319.154503510.1097/00004728-199203000-00027131

Schiller
F
. Tongue carcinoma in epidermolysis bullosa dystrophical. Arch Klin Exp Dermatol. 1960;209:643‐651.14442763132

Lotem
M
, 
Raben
M
, 
Zeltser
R
, et al. Kindler syndrome complicated by squamous cell carcinoma of the hard palate: successful treatment with high‐dose radiation therapy and granulocyte‐macrophage colony‐stimulating factor. Br J Dermatol. 2001;144(6):1284‐1286.1142207110.1046/j.1365-2133.2001.04262.x133

Saleva
M
, 
Has
C
, 
He
Y
, et al. Natural history of kindler syndrome and propensity for skin cancer ‐ case report and literature review [Internet]. JDDG ‐ J German Soc Dermatol. 2018;16:338‐341. 10.1111/ddg.13435.29384271134

Caldeira
A
, 
Trinca
WC
, 
Flores
TP
, et al. A Kindler syndrome‐associated squamous cell carcinoma treated with radiotherapy. Reports Pract Oncol Radiother. 2016;21(6):532‐536.10.1016/j.rpor.2016.07.004PMC502184727660560135

Souldi
H
, 
Bajja
MY
, 
Mahtar
M
. Kindler syndrome complicated by invasive squamous cell carcinoma of the palate. Eur Ann Otorhinolaryngol Head Neck Dis. 2018;135(1):59‐61.2864195710.1016/j.anorl.2017.05.003136

Has
C
, 
Wessagowit
V
, 
Pascucci
M
, et al. Molecular basis of Kindler syndrome in Italy: novel and recurrent Alu/Alu recombination, splice site, nonsense, and frameshift mutations in the KIND1 gene. J Invest Dermatol. 2006;126(8):1776‐1783.1667595910.1038/sj.jid.5700339137

Ashton
GHS
, 
McLean
WHI
, 
South
AP
, et al. Recurrent mutations in Kindlin‐1, a novel keratinocyte focal contact protein, in the autosomal recessive skin fragility and photosensitivity disorder, Kindler syndrome. J Invest Dermatol. 2004;122(1):78‐83.1496209310.1046/j.0022-202X.2003.22136.x138

Lanschuetzer
CM
, 
Muss
WH
, 
Emberger
M
, et al. Characteristic immunohistochemical and ultrastructural findings indicate that Kindler's syndrome is an apoptotic skin disorder. J Cutan Pathol. 2003;30(9):553‐560.1450740310.1034/j.1600-0560.2003.00119.x139

Peñarrocha‐Oltra
D
, 
Agustín‐Panadero
R
, 
Serra‐Pastor
B
, 
Peñarrocha‐Diago
M
, 
Peñarrocha‐Diago
M
. Oral rehabilitation with dental implants in patients with recessive dystrophic epidermolysis bullosa: a retrospective study with 2–15 years of follow‐up. Med Oral Patol Oral Cir Bucal. 2020;25(2):e262‐e267.3196798410.4317/medoral.23331PMC7103452140

O'Leary
TJ
, 
Drake
RB
, 
Naylor
JE
. The plaque control record. J Periodontol. 1972;43(1):38.450018210.1902/jop.1972.43.1.38141

Harris
JC
, 
Bryan
RA
, 
Lucas
VS
, 
Roberts
GJ
. Dental disease and caries related microflora in children with dystrophic epidermolysis bullosa. Pediatr Dent. 2001;23(5):438‐443.11699172142

Fortuna
G
, 
Aria
M
, 
Cepeda‐Valdes
R
, et al. Clinical features of gingival lesions in patients with dystrophic epidermolysis bullosa: a cross‐sectional study. Aust Dent J. 2015;60(1):18‐23.2572127510.1111/adj.12264143

Wright
JT
, 
Childers
NK
, 
Evans
KL
, 
Johnson
LB
, 
Fine
JD
. Salivary function of persons with hereditary epidermolysis bullosa. Oral Surg Oral Med Oral Pathol. 1991;71(5):553‐559.204709610.1016/0030-4220(91)90361-f144

Wright
JT
, 
Fine
JD
, 
Johnson
L
. Hereditary epidermolysis bullosa: oral manifestations and dental management. Pediatr Dent. 1993;15(4):242‐248.8247897145

Shah
H
, 
McDonald
F
, 
Lucas
V
, 
Ashley
P
, 
Roberts
G
. A cephalometric analysis of patients with recessive dystrophic epidermolysis bullosa. Angle Orthod. 2002;72(1):55‐60.1184327510.1043/0003-3219(2002)072<0055:ACAOPW>2.0.CO;2146

Liversidge
HM
, 
Kosmidou
A
, 
Hector
MP
, 
Roberts
GJ
. Epidermolysis bullosa and dental developmental age. Int J Paediatr Dent. 2005;15(5):335‐341.1612899710.1111/j.1365-263X.2005.00649.x147

Kostara
A
, 
Roberts
GJ
, 
Gelbier
M
. Dental maturity in children with dystrophic epidermolysis bullosa. Pediatr Dent. 2000;22(5):385‐388.11048306148

Sharma
S
, 
Bedi
S
. Dystrophic epidermolysis bullosa associated with non‐syndromic hypodontia. Indian Dermatol Online J. 2013;4(4):296‐299.2435000910.4103/2229-5178.120644PMC3853894149

Mars
M
, 
Houston
WJ
. A preliminary study of facial growth and morphology in unoperated male unilateral cleft lip and palate subjects over 13 years of age. Cleft Palate J. 1990;27(1):7‐10.230281810.1597/1545-1569(1990)027<0007:apsofg>2.3.co;2150

Fewtrell
MS
, 
Allgrove
J
, 
Gordon
I
, et al. Bone mineralization in children with epidermolysis bullosa. Br J Dermatol. 2006;154(5):959‐962.1663490110.1111/j.1365-2133.2005.07123.x151

Primo
BT
, 
da Costa
DJ
, 
Stringhini
DJ
, et al. Sialolithiasis in the duct of submandibular gland: a case report in patient with epidermolysis bullosa. J Contemp Dent Pract. 2013;14(2):339‐344.2381167010.5005/jp-journals-10024-1324152

Wright
JT
, 
Fine
J‐D
, 
Johnson
LB
, 
Steinmetz
TT
. Oral involvement of recessive dystrophic epidermolysis bullosa inversa. Am J Med Genet. 1993;47(8):1184‐1188.829155310.1002/ajmg.1320470811153

Pearson
RW
, 
Paller
AS
. Dermolytic (dystrophic) epidermolysis bullosa inversa. Arch Dermatol. 1988;124(4):544‐547.3355197154

Lin
AN
, 
Smith
LT
, 
Fine
J‐D
. Dystrophic epidermolysis bullosa inversa: report of two cases with further correlation between electron microscopic and immunofluorescence studies. J Am Acad Dermatol. 1995;33(2 II):361‐365.761588610.1016/0190-9622(95)91434-x155

Ricketts
DN
, 
Morgan
CL
, 
McGregor
JM
, 
Morgan
PR
. Kindler syndrome: a rare cause of desquamative lesions of the gingiva. Oral Surg Oral Med Oral Pathol Oral Radiol Endod. 1997;84(5):488‐491.939437910.1016/s1079-2104(97)90263-8156

Zhou
C
, 
Song
S
, 
Zhang
J
. A novel 3017‐bp deletion mutation in the FERMT1 (KIND1) gene in a Chinese family with Kindler syndrome. Br J Dermatol. 2009;160(5):1119‐1122.1929271810.1111/j.1365-2133.2009.09052.x157

Yazdanfar
A
, 
Hashemi
B
. Kindler syndrome: report of three cases in a family and a brief review. Int J Dermatol. 2009;148:145‐149.10.1111/j.1365-4632.2009.03936.x19200189158

Has
C
, 
Yordanova
I
, 
Balabanova
M
, et al. A novel large FERMT1 (KIND1) gene deletion in Kindler syndrome. J Dermatol Sci. 2008;52:209‐212.1883576010.1016/j.jdermsci.2008.07.007159

Krishna C
V
, 
Parmar N
V
, 
Has
C
. Kindler syndrome with severe mucosal involvement in childhood. Clin Exp Dermatol. 2014;39(3):340‐343.2463507510.1111/ced.12293160

Chimenos Küstner
E
, 
Fernández Fresquet
R
, 
López López
J
, 
Rodríguez de Rivera Campillo
E
. Kindler syndrome: a clinical case. Med Oral. 2003;8(1):38‐44.12556722161

Mansur
AT
, 
Elcioglu
NH
, 
Aydingöz
IE
, et al. Novel and recurrent KIND1 mutations in two patients with Kindler syndrome and severe mucosal involvement. Acta Dermato‐Venereologica. 2007;87:563‐565.1798990710.2340/00015555-0314162

Anwar
MI
, 
Rashid
A
, 
Ghafoor
R
, et al. Kindler's syndrome: a report of five cases in a family. J Coll Physicians Surg Pakistan. 2014;24(10):763‐765.10.2014/JCPSP.76376525327923163

Wada
M
, 
Masuda
K
, 
Tsuruta
D
, et al. Case of Kindler syndrome resulting from mutation in the FERMT1 gene [Internet]. J Dermatol. 2012;39:1057‐1058.2267206010.1111/j.1346-8138.2012.01598.x164

Ghosh
SK
, 
Bandyopadhyay
D
, 
Das
J
, 
Chatterjee
G
, 
Sarkar
S
. Kindler's syndrome: a case series of three Indian children. Indian J Dermatol. 2010;55(4):393‐6.2143090010.4103/0019-5154.74568PMC3051307165

Gkaitatzi
M
, 
Kalloniati
E
, 
Has
C
, et al. Kindler syndrome: a rare case report from Greece. Oxford Med Case Reports. 2019;2019(2):103‐105.10.1093/omcr/omz003PMC639640730838128166

Ohashi
A
, 
Kiniwa
Y
, 
Okuyama
R
, et al. A case of Kindler syndrome with severe esophageal stenosis. Int J Dermatol. 2015;54:e106‐8.2555642210.1111/ijd.12715167

Suga
Y
, 
Tsuboi
R
, 
Hashimoto
Y
, 
Yaguchi
H
, 
Ogawa
H
. A Japanese case of Kindler syndrome. Int J Dermatol. 2000;39(4):284‐286.1080997810.1046/j.1365-4362.2000.00962.x168

Sharma
RC
, 
Mahajan
V
, 
Sharma
NL
, 
Sharma
AK
. Kindler syndrome. Int J Dermatol. 2003;42(9):727‐732.1295669110.1046/j.1365-4362.2003.01659.x169

Wiebe
CB
, 
Silver
JG
, 
Larjava
HS
. Early‐onset periodontitis associated with Weary‐Kindler syndrome: a case report. J Periodontol. 1996;67(10):1004‐1010.891084010.1902/jop.1996.67.10.1004170

Wiebe
CB
, 
Petricca
G
, 
Häkkinen
L
, et al. Kindler syndrome and periodontal disease: review of the literature and a 12‐year follow‐up case. J Periodontol. 2008;79(5):961‐966.1845467810.1902/jop.2008.070167PMC2697853171

Ashton
GHS
. Kindler syndrome. Clin Exp Dermatol. 2004;29(2):116‐121.1498726310.1111/j.1365-2230.2004.01465.x172

Hacham‐Zadeh
S
, 
Garfunkel
AA
, 
Opitz
JM
, 
Reynolds
JF
. Kindler syndrome in two related Kurdish families. Am J Med Genet. 1985;20(1):43‐48.397007310.1002/ajmg.1320200107173

Kartal
D
, 
Borlu
M
, 
Has
C
, 
Folster‐Holst
R
. A novel mutation in the FERMT1 gene in Turkish siblings with Kindler syndrome. J Eur Acad Dermatol Venereol. 2016;30:1233‐1235.2586528810.1111/jdv.13163174

Lennartz
L
, 
Has
C
, 
Lehmann
P
. Kongenitale bullöse Poikilodermie (Kindler‐Syndrom) ‐ neue Mutation [Internet]. JDDG ‐ J German Soc Dermatol. 2012;10:919‐920.10.1111/j.1610-0387.2012.08050.x23078512175

Gupta
V
, 
Dogra
D
, 
Gupta
N
, 
Parveen
S
. Kindler's syndrome with long thick cuticles and mottled hyperpigmentation [Internet]. Indian J Dermatol Venereol Leprol. 2011;77:66‐68.2122088610.4103/0378-6323.74991176

Diociaiuti
A
, 
Zambruno
G
, 
Giancristoforo
S
, et al. Acral skin atrophy in an infant: an early clue to Kindler syndrome diagnosis. J Eur Acad Dermatol Venereol. 2016;30:1046‐1049.2576410610.1111/jdv.13101177

Khan
IU
, 
Fazal
S
. Kindler's syndrome: a case report. J Pakistan Assoc Dermatologists. 2008;18(1):49‐52.178

Kantheti
P
, 
Kubba
A
, 
Prabhu
A
, 
Batrani
M
, 
Hiremagalore
R
. Two novel mutations in KIND1 in Indian patients with Kindler syndrome. Clinical Exp Dermatol. 2017;42:95‐97.10.1111/ced.1294627862150179

Barbosa
NM
, 
Visioli
F
, 
Martins
MD
, 
Martins
MAT
, 
Munerato
MC
. Oral manifestations in Kindler syndrome: case report and discussion of literature findings. Spec Care Dent. 2016;36(4):223‐230.10.1111/scd.1216526815761180

Mendiratta
V
, 
Malik
M
. Kindler syndrome. Indian Pediatr. 2018;55(1):85.29396956181

Kargar
S
, 
Shiryazdi
SM
, 
Neamatzadeh
H
, et al. Kindler syndrome: the case of two Iranian sisters. G Ital di Dermatologia e Venereol. 2018;153(1):111‐114.10.23736/S0392-0488.16.04887-227391311182

Angelova‐Fischer
I
, 
Kazandjieva
J
, 
Vassileva
S
, 
Dourmishev
A
. Kindler syndrome: a case report and proposal for clinical diagnostic criteria. Acta Dermatoven APA. 2005;14(2):61‐67.16001103183

Malik
LM
, 
Azfar
NA
, 
Jamil
A
, 
Jahangir
M
. A case of Kindler syndrome with florid scabies. J Pakistan Assoc Dermatology. 2010;20:45‐49.184

Mas‐Vidal
A
, 
Miñones‐Suárez
L
, 
Toral
JF
, 
Mallo
S
, 
Pérez‐Oliva
N
. A novel mutation in the FERMT1 gene in a Spanish family with Kindler's syndrome [Internet]. J Eur Acad Dermatol Venereol. 2010;24:978‐979.2002844110.1111/j.1468-3083.2009.03554.x185

Penagos
H
, 
Jaen
M
, 
Sancho
MT
, et al. Kindler syndrome in native Americans from Panama: report of 26 cases. Arch Dermatol. 2004;140(8):939‐944.1531380910.1001/archderm.140.8.939186

Nofal
E
, 
Assaf
M
, 
Elmosalamy
K
. Kindler syndrome: a study of five Egyptian cases with evaluation of severity. Int J Dermatol. 2008;47(7):658‐662.1861386910.1111/j.1365-4632.2008.03721.x187

El Hachem
M
, 
Diociaiuti
A
, 
Proto
V
, et al. Kindler syndrome with severe mucosal involvement in a large Palestinian pedigree. Eur J Dermatol. 2015;25(1):14‐19.2551559810.1684/ejd.2014.2457188

Has
C
, 
Castiglia
D
, 
del Rio
M
, et al. Kindler syndrome: extension of FERMT1 mutational spectrum and natural history. Hum Mutat. 2011;32(11):1204‐1212.2193602010.1002/humu.21576189

Roda
A
, 
Travassos
AR
, 
Soares‐de‐Almeida
L
, 
Has
C
. Kindler syndrome in a patient with colitis and primary sclerosing cholangitis: coincidence or association?
Dermatol Online J. 2018;24(3):13030.29634879190

Dobrev
HP
, 
Vutova
NI
. Nailfold capillaroscopic changes in Kindler syndrome. Intractable Rare Dis Res. 2015;4(4):214‐216.2666878410.5582/irdr.2015.01038PMC4660865191

Gökalp
H
, 
Önder
M
. Kindler syndrome; new FERMT‐1 gene mutation and breast cancer. Turkiye Klin Dermatoloji. 2012;22(1):67‐70.192

Gomathy
S
, 
McGrath
JA
, 
Cheong
L
, 
Kabra
M
, 
Sharma
VK
. Kindler syndrome in India. J Am Acad Dermatol. 2013;68(4):AB175‐AB175.193

Techanukul
T
, 
Sethuraman
G
, 
Zlotogorski
A
, et al. Novel and recurrent FERMT1 gene mutations in Kindler syndrome. Acta Derm Venereol. 2011;91(3):267‐270.2133647510.2340/00015555-1063194

de Almeida
HL
, 
Heckler
GT
, 
Fong
K
, 
Lai‐Cheong
J
, 
McGrath
J
. Sporadic Kindler syndrome with a novel mutation. An Bras Dermatol. 2013;88(6 Suppl 1):212‐215.2434692310.1590/abd1806-4841.20132173PMC3875998195

Mendes
L
, 
Nogueira
L
, 
Vilasboas
V
, et al. Síndrome de Kindler ‐ relato de dois casos. An Bras Dermatol. 2012;87(5):779‐781.2304457610.1590/s0365-05962012000500020196

Gao
Y
, 
Bai
J
, 
Liu
X
, et al. A novel large deletion mutation of FERMT1 gene in a Chinese patient with Kindler syndrome. J Zhejiang Univ Sci B. 2015;16(11):957‐962.2653721410.1631/jzus.B1500080PMC4642877197

El Fekih
N
, 
Mahfoudh
A
, 
Zekri
S
, et al. Kindler syndrome: clinical and ultra‐structural particularities, a propos of three cases. Ann Pathol. 2011;31(4):246‐250.2183934710.1016/j.annpat.2011.05.009198

Downey
C
, 
Rio
MD
, 
Escamez
MJ
, 
Baselga
E
. Keratosis punctate: a clinical manifestation of kindler syndrome. Pediatr Dermatol. 2017;34:S130.199

Kavala
M
, 
Südoǧan
S
, 
Can
B
, 
Albayrak
Ö
. A case of kindler syndrome. Turkderm Deri Hast ve Frengi Ars. 2007;41(1):28‐30.200

Chmel
N
, 
Danescu
S
, 
Gruler
A
, et al. A deep‐intronic FERMT1 mutation causes kindler syndrome: an explanation for genetically unsolved cases. J Invest Dermatol. 2015;135:2876‐2879.2608355210.1038/jid.2015.227201

Atzori
L
, 
Lai
M
, 
Lappi
A
, 
Brundu
MA
, 
Rongioletti
F
. Erosive pustular dermatosis of the scalp and Kindler syndrome: a new association. J Eur Acad Dermatol Venereol. 2018;32:e102‐e104.2886980410.1111/jdv.14577202

Emanuel
PO
, 
Rudikoff
D
, 
Phelps
RG
. Aggressive squamous cell carcinoma in Kindler syndrome. Skinmed. 2006;5(6):305‐307.1708600210.1111/j.1540-9740.2006.05369.x203

Wiebe
CB
, 
Larjava
HS
. Abnormal deposition of type VII collagen in Kindler syndrome. Arch Dermatol Res. 1999;291(1):6‐13.1002572210.1007/s004030050377204

Yildirim
TT
, 
Kaya
FA
, 
Taskesen
M
, et al. Aggressive periodontitis associated with Kindler syndrome in a large Kindler syndrome pedigree. Turk J Pediatr. 2017;59(1):56‐61.2916836410.24953/turkjped.2017.01.009205

Fortuna
G
, 
Chainani‐Wu
N
, 
Lozada‐Nur
F
, et al. Epidermolysis Bullosa Oropharyngeal Severity (EBOS) score: A multicenter development and reliability assessment. J Am Acad Dermatol. 2013;68(1):83‐92.2257515810.1016/j.jaad.2012.04.009

## CHAPTER 3: Oral health care and dental treatment for children and adults living with epidermolysis bullosa—Clinical practice guidelines

Susanne Krämer | James Lucas | Francisca Gamboa | Miguel Peñarrocha Diago | David Peñarrocha Oltra | Marcelo Guzmán‐Letelier | Sanchit Paul | Gustavo Molina | Lorena Sepúlveda | Ignacio Araya | Rubén Soto | Carolina Arriagada | Reinhard Schilke | Mark Adam Antal | Fernanda Castrillón | Victoria Clark

### Introduction

Children and adults with EB present fragile skin and mucosa, as well as specific oral features, as described in the previous chapter, requiring a special approach from the dental perspective. The present article is and update of the 2012 CPG on oral health care for patients with EB^1^, including new evidence and a wider panel of international clinical experts. The present CPG follows a standard methodology based on a systematic review of the currently available scientific evidence.

### Aim

To provide the users with information on the current best practices for managing the oral health care of people living with EB.

### Health question covered in the guideline

Do patients with EB need specific precautions or treatment modifications compared to healthy individuals to avoid trauma to the skin and mucosa while providing dental care?

### Users

Specialists in Pediatric Dentistry, Special Care Dentistry, Orthodontics, Oral and Maxillofacial Surgery, Endodontics, Periodontics, Rehabilitation and General Dental Practitioners, Dental hygienists, Pediatricians, Dermatologists, Otolaryngologists (ENT), Nurses, Dietitians, Speech and Language Therapists, parents, and those living with Inherited Epidermolysis Bullosa.

### Target group

These guidelines can be applied to all patients diagnosed with EB. As such, the guideline considers information for all four major types of EB: EB Simplex (EBS), Junctional EB (JEB), Dystrophic EB (DEB), and Kindler EB (KEB).

### Methodology

#### Systematic Literature Searching

##### Literature sources

A systematic literature review was performed to identify all the oral care and treatment precautions for patients with EB. The literature search ranged from 2010 to March 2018. Consulted sources included the electronic databases MEDLINE (2010 to March 2018), EMBASE (2010 to March 2018), CINAHL (2010 to March 2018), The Cochrane Library (2010), DARE (2010), and the Cochrane controlled trials register (CENTRAL) (2010). In addition, hand searching journals, reviewing conference proceedings, and other guidelines sources such as DEBRA International Guideline depository were carried out. The reference lists of all papers for relevant citations were reviewed. When all the relevant studies were identified, they were sent to the experts to review for completeness.

##### Selection criteria of the articles

Selection criteria of the articles: primary or secondary articles in which the main topic is oral care and precautions during dental treatment (diagnosis, and⁄or treatment and⁄or prognosis) of patients with EB, published between 2010 and 2018 in any language.

##### Search strategy

To identify studies for this review, detailed search strategies were based on the search strategy developed for MEDLINE but revised appropriately for each database. The search strategy used a combination of controlled vocabulary and free text terms based on:

#1 "Epidermolysis Bullosa"[Mesh],
#2 ((Epidermolysis[tiab] OR Acantholysis[tiab])) AND Bullosa[tiab]
#3 "Dentistry"[Mesh]
#4 "Oral Health"[Mesh]
#5 "Mouth Diseases"[Mesh]
#6 "Dentistry"[tiab]
#7: #1 OR #2;
#8: #3 OR #4 OR #5 OR #6;
#9: #7 AND #8;
#10: #9 AND ("2010/11/01"[PDat]: "2018/03/01"[PDat]).

##### Methods used for formulating the recommendations

To formulate the recommendations of the selected studies, the SIGN system was used as described on the 50 Guideline Developer's Handbook, NHS Scottish Intercollegiate Guidelines Network SIGN. Revised Edition January 2008 Figure 3.1.^2^

**FIGURE 3.1:**
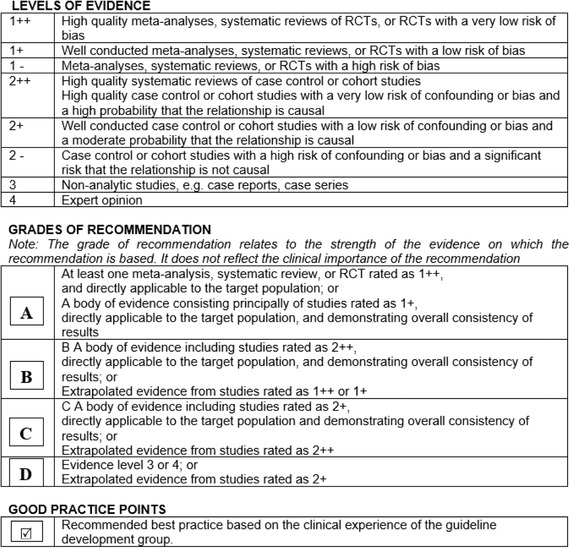
Method used for formulating the recommendations as described on the 50 Guideline Developer's Handbook, NHS Scottish Intercollegiate Guidelines Network SIGN. Revised Edition January 2008^2^

#### Guideline development process

The information from the selected studies was gathered in a draft document by the clinical leads and methodologists. The draft document was analyzed and discussed at a consensus meeting held in Dubai on August 30th, 2018, by the clinical leads, clinical experts, and patient representatives guided by the methodologists as described in Tables 1‐4 (page 2 to 4). The consensus report was reviewed by external specialists and health care professionals from the multidisciplinary team (Table 5, page 4), as well as patients and representatives from the DEBRA association groups from Australia, Brazil, New Zealand, Spain, and United States (Table 3, page 3). They reviewed the document to ensure that the degree to which the evidence addresses patients' concerns is reflected in the guideline. The final version was piloted in four centers in four different countries for three months as described in Table 6 on page 4 (September 2019 to December 2019).

#### Guideline implementation and monitoring

##### Implementation barriers

According to the context of implementation of this guideline, some barriers to be considered are:

▪ Access to training in EB specific issues.
▪ Insufficient availability of health services in some parts of the world.
▪ To improve implementation (to reduce barriers) a broadcast of the guideline will be developed by the guideline development panel and uploaded for open access on DI web page.
▪ Dentists experts in EB should be encouraged in organizing training for local dentists to motivate them to treat people with EB and give them confidence.

##### Guideline monitoring and/or auditing criteria

The implementation of these recommendations could be monitored and evaluated through audits and completing of the “CPG Evaluation Form: Pre‐implementation” (available at https://surveyhero.com/c/aabc0100). The panel recommends clinical sites to conduct prepractice audit, implement the CPG, and reaudit to test improvements. Audit tools can be used from SIGN35. DEBRA International would value your feedback on the site findings to continue to improve CPG quality.

##### Further areas of research

- Continuous follow‐up of the recommendations stated in this guideline.
- Treatment of oral ulcers in patients with EB.

##### Guideline updating procedure

The guideline will be updated every 5 years after its second version. If new relevant evidence is detected before the update, the information will be published on the web site http://www.debra-international.org/. The team in charge of this update will be formed by Prof. Susanne Krämer and Prof. James Lucas in 2025.

### 3.1 Oral care for patients with inherited epidermolysis bullosa

#### Introduction


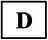
 A preventive protocol is today's dental management approach of choice.^3^, ^4^, ^5^

The approach to dental treatment for patients with EB, in particular for those with the more severe types, has changed dramatically over the last 40 years. Crawford et al in 1976^6^ considered extraction of all teeth to be the treatment of choice for patients with RDEB. Two decades later, in 1999, Wright^7^ declared that it was possible to manage dental abnormalities successfully with a combination of anesthetic and restorative techniques. In 2008, Skogedal et al^4^ advocated that caries can be successfully prevented in patients with RDEB by continuous follow‐up aimed at dietary advice, oral hygiene habits, frequent professional cleaning, and fluoride therapy. For those adult patients who did not successfully access a preventive approach and have lost their teeth, Peñarrocha and coworkers demonstrated a high success rate of implant supported complete oral rehabilitation.^8^

**Importance of oral preventive care and dental treatment: Patient perspective**

This list was ordered according to the preferences of the patients and their representatives in the consensus meetings hold in Santiago in 2010 and restated in the consensus meeting in Dubai 2018.

1. To prevent and treat pain and infection. This is important considering that patients with oral pain will reduce their nutritional intake.
2. To improve aesthetics and self‐esteem.
3. A healthy dentition improves the patient's ability to chew and swallow that improves the nutritional status. Maintaining a functional dentition also reduces the potential for oral and esophageal soft tissue damage through more efficient mastication.
4. Improved phonetics: when anterior teeth are restored, allowing for better positioning of the tongue.
5. Improved swallowing: maintaining a healthy dentition provides the structures needed for oral functioning, more precisely to complete the preparatory phase of swallowing.
6. Maintaining a harmonious relationship between teeth stabilizes the occlusion for better function, aesthetics, and allows for better hygiene.

#### 3.1.1 Access to dental clinic

Accessibility of dental care can be limited for some patients. Although in most developed countries, dental care is presumed to be guaranteed, it remains a privilege for many patients globally. There is a lack of knowledge about the disease in the dental profession^9^ and other health care professionals. Dental care can be complicated by the fears of both the patient and the dentist.^10^ Providers who regularly care for patients with EB find allowing plenty of time and being very gentle can improve the patients confidence, and treatment success. For example, it is important to pay attention to the surface that the patient is laying on, how the patient's face is touched, or head is held. Even the simplest of procedures, such as an oral exam, takes longer due to the limited oral access and the discomfort or fear of developing blisters secondary to soft tissue manipulation.


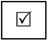
 The clinic must be of easy access for patients using wheelchairs and walking frames.


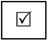
 If the patient must travel a long distance to attend the specialist dentist in the EB unit, a shared care approach can be arranged with a local dentist, who can provide more regular preventive care.

#### 3.1.2 Early referral


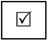
 Dental care is part of the multidisciplinary team in EB, and therefore patients should be referred to the dentist immediately upon diagnosis. Patients should be referred to the dentist before oral problems present (ideally 3 to 6 months); as early referral and close follow‐up are the key to keeping patients as healthy as possible from the oral point of view.

**IMAGE 3.1:**
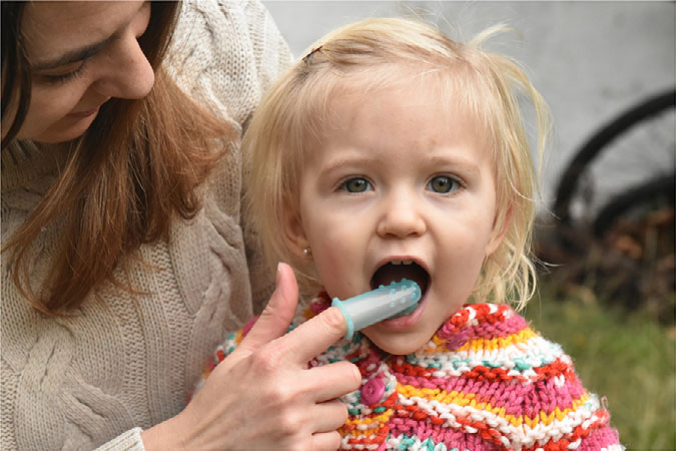
Early oral hygiene instruction with a finger guard brush

**IMAGE 3.2:**
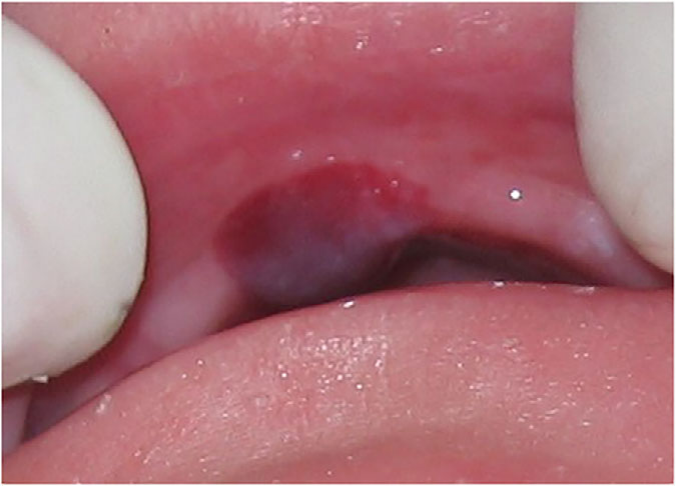
Two‐week‐old newborn with severe RDEB. Early diagnosis and education to parents on bullae management

**IMAGE 3.3:**
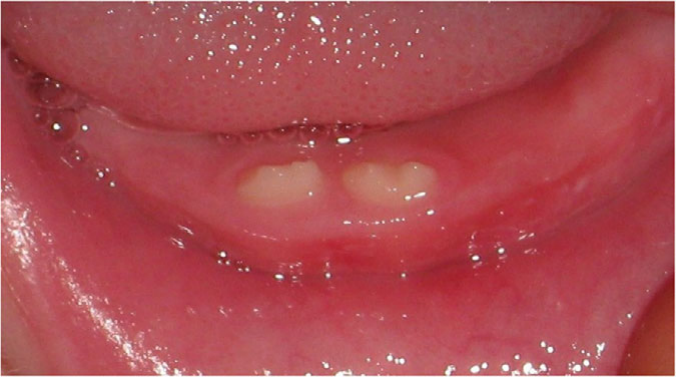
Early diagnosis of generalized enamel hypoplasia in an 8 months old child with JEB

The first consultation should be aimed at:

- Education of the parents and caregivers: Counseling on diet (including sugar free medications), oral hygiene routines (Image 3.1), fluorides, technical aids, and oral manifestations of EB. This preventive advice should be provided even before the teeth erupt (Image 3.2).
- Early diagnosis of enamel abnormalities such as those seen in junctional EB (JEB). This is possible as soon as the first primary tooth erupts (Image 3.3).
- Early diagnosis of tooth crowding, mainly in recessive dystrophic EB (RDEB).
- Early diagnosis of incipient caries lesions.

Patients with EB should be referred to a dentist as early as possible to identify any feature related to EB that needs special attention, for example, generalized enamel hypoplasia.^7^, ^10^, ^11^, ^12^ This enables dentists to start preventive programs and reduces the risk of developing dental diseases.^13^, ^14^ Many case reports have shown that patients visit the dentist only when they already have several carious lesions or pain.^11^, ^15^, ^16^, ^17^, ^18^

#### 3.1.3 Oral assessment

Dental evaluation can be aimed at: (a) identifying treatment needs, (b) identifying oral features of EB, or (c) measuring disease activity and structural damage (ie, severity of the disease).

The Epidermolysis Bullosa Oropharyngeal Severity Score (EBOS) was developed to fulfill the third aim: quantifying the oropharyngeal severity in different types/subtypes of EB, rather than any possible scarring phenotype.^19^, ^20^ This could be an important tool when studying the impact of different therapies on the clinical features of the condition. The score has shown strong intraobserver reliability^19^ and a lower interobserver reliability.^21^ The score was designed to evaluate the key features of disease activity: erythema, atrophy, blistering, and erosion and ulceration; as well as the presence or absence of four clinical parameters: microstomia, ankyloglossia, and intraoral scaring such as vestibular obliteration and enamel hypoplasia.^19^ Later publications, however, have discussed whether enamel hypoplasia, for example, measures disease severity or only correlates to altered genes that cause structural damage of the enamel. It has been proposed to either evaluate it separately in another scale or considered as a different measure.^22^ Also, it is still to be confirmed whether buccal vestibule and floor of the mouth evaluation should be left or removed from the EBOS scale.^22^ Studies using this tool have not found strict genotype‐oropharyngeal phenotype correlations in DEB.^23^


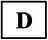
 Patients with RDEB and Kindler EB are at increased risk of developing intraoral SCC. Screening is important as early as during the third decade in RDEB^7^, ^24^, ^25^, ^26^, ^27^ and fourth decade in KEB.^28^, ^29^, ^30^, ^31^, ^32^, ^33^, ^34^

#### 3.1.4 Behavioral support

Dental treatment can be a challenge due to sensitivity of the mucosa, constant presence of blisters, risk of causing new lesions, microstomia,^35^, ^36^ limited ability for full cooperation from the patient, and risk of causing new lesions during protective stabilization due to the fragility of the skin. With appropriate behavioral support, patients can gain confidence in the dental team and cooperate to the best of their ability with treatment.^14^ Behavioral support should be patient centered and must involve the whole dental team who should agree on goals and roles and ensure that any plan to facilitate care is proportionate to the benefits of the proposed treatment.

As sedation and general anesthesia are techniques often used for providing dental treatment for patients with EB, a whole chapter has been dedicated to that topic in this special issue of the journal: Chapter 5: Sedation and anesthesia for adults and children with EB undergoing dental treatment—Clinical Practice Guidelines.

#### 3.1.5 Skin management

There are some general aspects dentists should consider when assessing or treating patients with EB to reduce the occurrence of new blisters and wounds. The environment should be cool and air conditioned as overheating can increase skin fragility.^37^ At any age, no tape or adhesive should be applied directly onto the skin; nonadherent wound dressings can be used instead (see table 5.1 on page 75).^38^, ^39^ Babies with EB should be lifted by placing one hand behind the child's bottom and one hand behind the neck, rather than from under the arms, to minimize friction and blister development in this area. The patient should be transferred by gentle lifting rather than sliding.^37^ A topical barrier cream or emollient such as Vaseline^®^ (Unilever, London, UK) or Linovera^®^ (B.Braun, Melsungen, Germany) should be used when examining the oral cavity to prevent direct contact with the oral/facial tissues. If using Vaseline, beware to keep any sources of oxygen away as may be hazardous.

#### 3.1.6 Patient positioning


**IMAGE 3.4:**
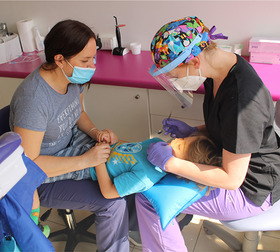
Knee‐to‐knee position for examining a 3‐year‐old patient

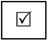
 Allow patients to position themselves in their own time or allow parent/caregiver to position the child in a comfortable position, as they are familiar with how best to handle their child. Do not try to assist them if you are not aware of the areas where they have wounds.


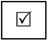
 Consider padding the dental chair or ask patients to bring any pressure reduction item such as a wipeable seat cushion, blanket or mattress topper.


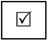
 Give patients breaks to rest and change position, according to their needs.


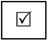
 For very small children, consider examining on parent's lap using the “knee to knee” technique (Image 3.4).

Even though most patients do not express discomfort in relation to the lesions on their back while sitting on dental chairs,^35^ supplemental padding can be used during dental treatment to prevent potential friction trauma to the skin.^35^

Patients should place themselves on the operating table if possible.^40^ A stretcher should be padded with a wipeable soft material, seat cushion, or mattress topper.^3^, ^41^, ^42^ Transfer and position changing should be done by moving the blanket, as patients should not slide on/across areas.^42^ If available, slide sheets can be used to aid patient positioning. Ensure that all team members are aware they need to lift and not slide the patient onto the table.

**IMAGE 3.5:**
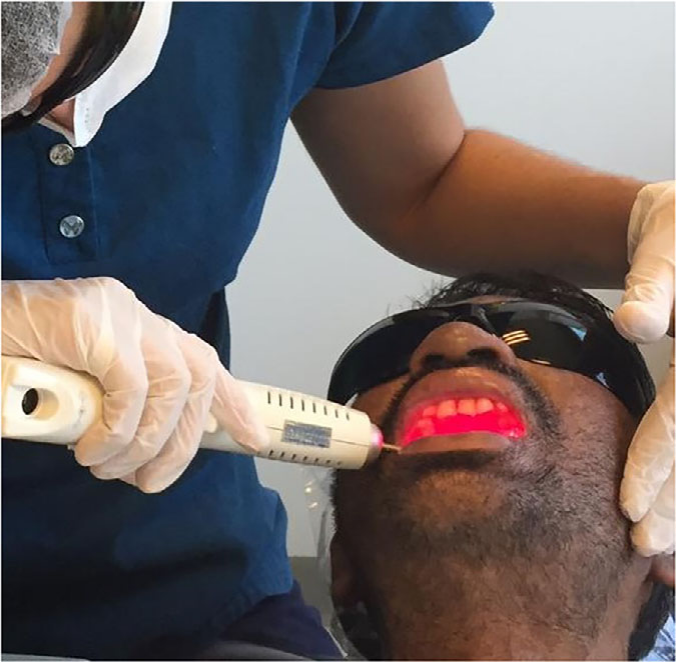
Thirty‐three‐year‐old patient with EB having low‐level laser therapy (LLLT) after oral surgery to reduce pain

#### 3.1.7 Oral bullae and ulcerations

Although oral bullae, ulcers, and erosions are the most common oral feature of EB, there are only two published studies of therapeutic approaches for these oral lesions. In 2001, Marini and Vecchiet^43^ described that sucralfate suspension reduced the development and duration of oral mucosal blisters and ulcers, reduced the associated oral pain, and improved plaque and gingival inflammation indices.^43^ In 2017, Sindici and colleagues^44^ published a pilot evaluation of the use of cord blood platelet gel (CBPG) and low‐level laser therapy (LLLT) over a 3‐day treatment period: one application daily on 19 long‐standing symptomatic oral lesions of seven patients with dystrophic EB. Reported pain and clinical size of lesions improved from the first day of treatment provided, reducing discomfort from ulceration. During the follow‐up period, only one patient developed a new lesion in the same treatment site; all patients continued to have other oral lesions at untreated sites. The only adverse effect reported was the unpleasant taste of the medication that was still reported by two patients (28%) after 24 weeks.^44^

**IMAGE 3.6:**
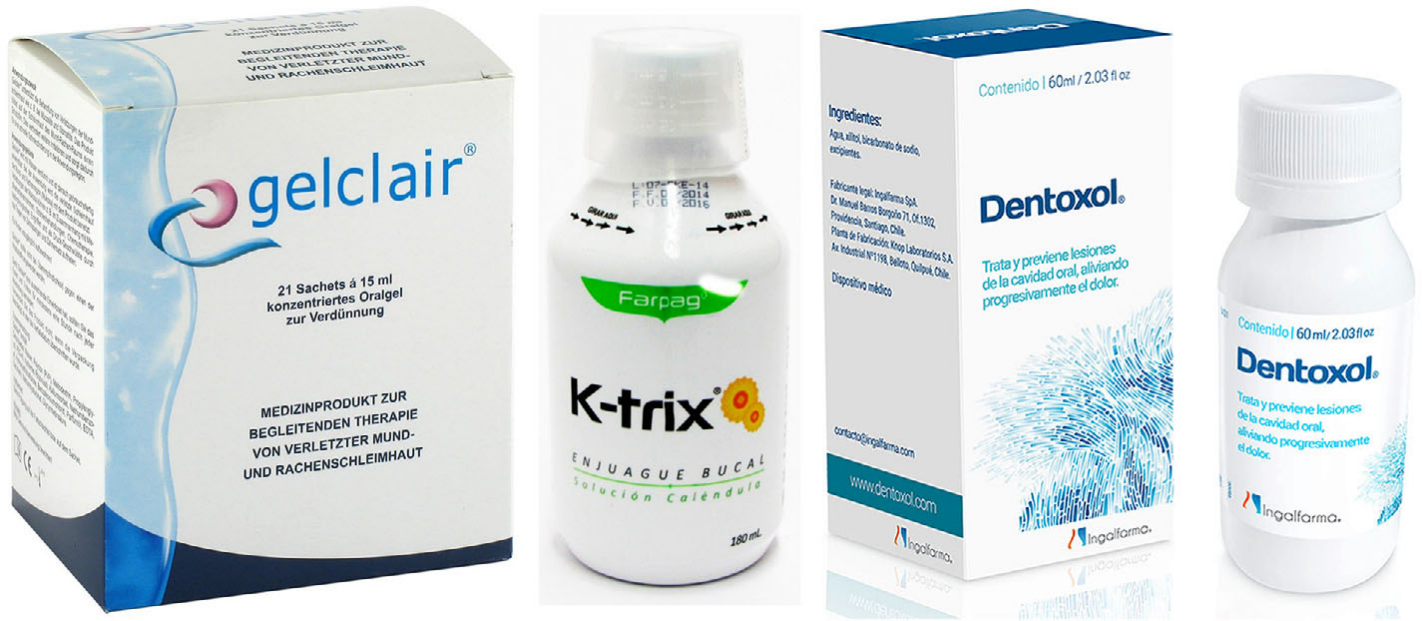
(A)‐(C) Mouth washes and oral gels aimed at wound healing

In addition to these strategies, mouthwashes and oral gels aimed at managing mucositis and oral lesions are commonly prescribed to patients with EB. The availability of these mouthwashes will vary between countries, and the clinical effectiveness will also vary among patients. Some of the products available are: Gelclair^®^ (Helsinn Healthcare SA, Switzerland), K‐trix^®^ (calendula based; Farpag, Colombia), and Dentoxol^®^ (Ingalfarma, Chile) (Image 3.6A–C). Several patients report the use of gargling saltwater as a cost‐effective and readily available alternative. There is a lack of published scientific reports on their effectiveness in EB. Randomized controlled trials are needed to determine the best treatment strategies.

#### 3.1.8 Preventive strategies

##### Partnership

A partnership approach between the family, the patient, and the dentists is fundamental for achieving and maintaining an adequate oral health status. The information transmitted to parents or caregivers should take into account their background knowledge on dental care, socioeconomic status, educational level, and the directions should be simple and easy to follow.^45^

##### Oral hygiene

**At home**

Concern is expressed by some patients, parents, and dentists regarding the use of toothbrushes and potential damage to the oral mucosa. Some patients find it difficult to perform oral hygiene due to oral lesions^14^, ^46^ bleeding, blisters, limited mouth opening,^45^, ^47^ and parents’ fear of causing pain.^48^ Studies have found that those patients who have been instructed on oral hygiene brush with a similar frequency, but use dental floss less regularly than those without EB.^47^

Even though patients might develop mitten deformities on their hands, only few patients have raised this problem as an issue for holding a toothbrush.^45^ Similar disparity exists with regard to the use of dental floss. Some authors strongly advocate for its use,^45^ others have proven its difficulty.^35^

**IMAGE 3.7:**
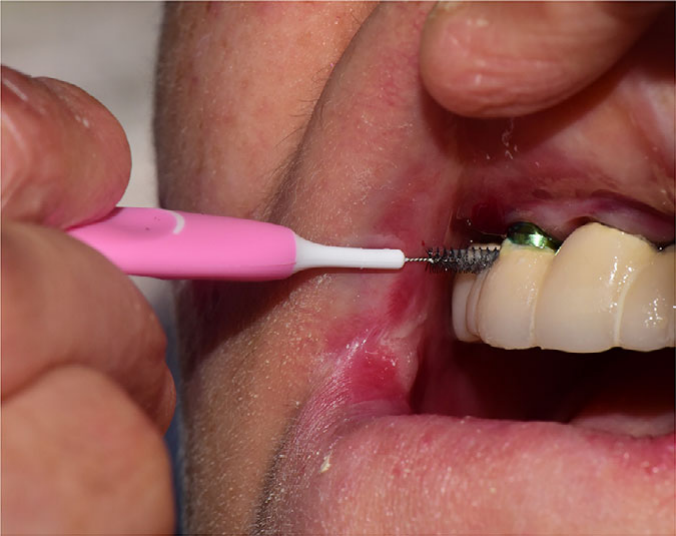
Forty‐one‐year‐old patient with intermediate RDEB cleaning her fixed denture with an interdental brush


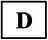
 Toothbrushing is possible in all patients with EB, even in patients with severe RDEB. The following suggestions can help determine the appropriate toothbrush for each patient:

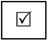

**IMAGE 3.8:**
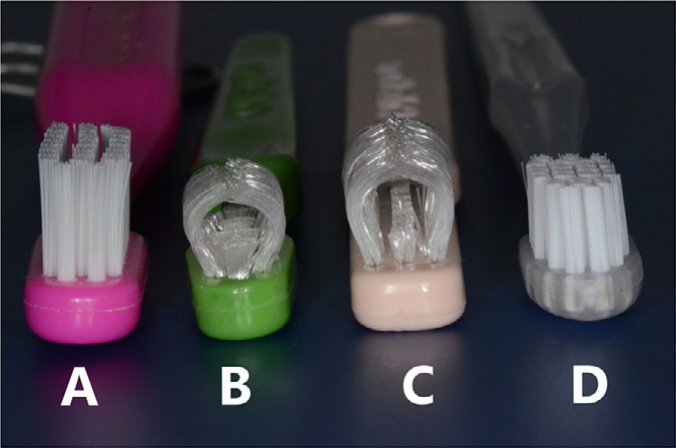
Examples of toothbrushes available for patients with limited mouth opening: (A) standard toothbrush. (B) Collis Curve baby toothbrush, (C) Collis Curve Junior Toothbrush, and (D) Pro Super‐fine (Esro AG) Toothbrush

**IMAGE 3.9:**
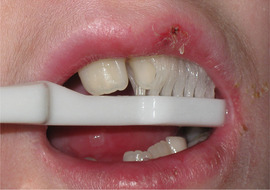
Collis Curve™ toothbrush (Collis‐Curve Toothbrush, TX, USA) cleans the palatal and buccal sides of the teeth simultaneously

  Small head.^7^, ^11^, ^15^, ^49^
- Soft bristle.^7^, ^11^, ^49^, ^50^
- The smallest toothbrush available (such as a baby‐size toothbrush^35^, ^45^) should be used.
- Bristles can be further softened by soaking them in warm/hot water.^10^
- In patients with severe microstomia, short bristles are indicated to access occlusal surfaces of molars. If there are no commercial short bristle toothbrushes available, bristles can be cut. If bristles are cut, one needs to ensure that they remain soft and do not harm the tissue.
- Parents or caregivers are advised to assist children, to improve plaque removal and helping to reduce the risk of tissue damage.^15^ Occasionally, adolescents and adults will also require support from caregivers for daily oral hygiene to increase effectiveness in areas difficult to reach.
- A manual toothbrush may be preferable to an electric brush, because of the increased possibilities of generating tissue trauma or bullae.
- Special toothbrushes, as, for example, Collis Curve^®^ toothbrush, Dr. Barman's Superbrush^®^, and Oralieve 360° Toothbrush^®^ might be good options for patients with RDEB, but more research on its efficiency is needed (Images 3.8 and 3.9).
- Finger guard brushes can be used by parents/carers as bristles are soft (Image 3.1).
- Special adaptations of the toothbrush handle can be advantageous for patients with pseudosyndactyly and manual dexterity problems. An orthotic such as the Oliber^®^ could be useful for patients with pseudosyndactyly (Image 3.11).

**IMAGE 3.10:**
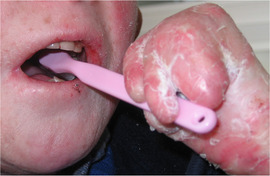
Thirty‐year‐old patient with RDEB and pseudosyndactyly performing oral hygiene with a small handle

**IMAGE 3.11:**
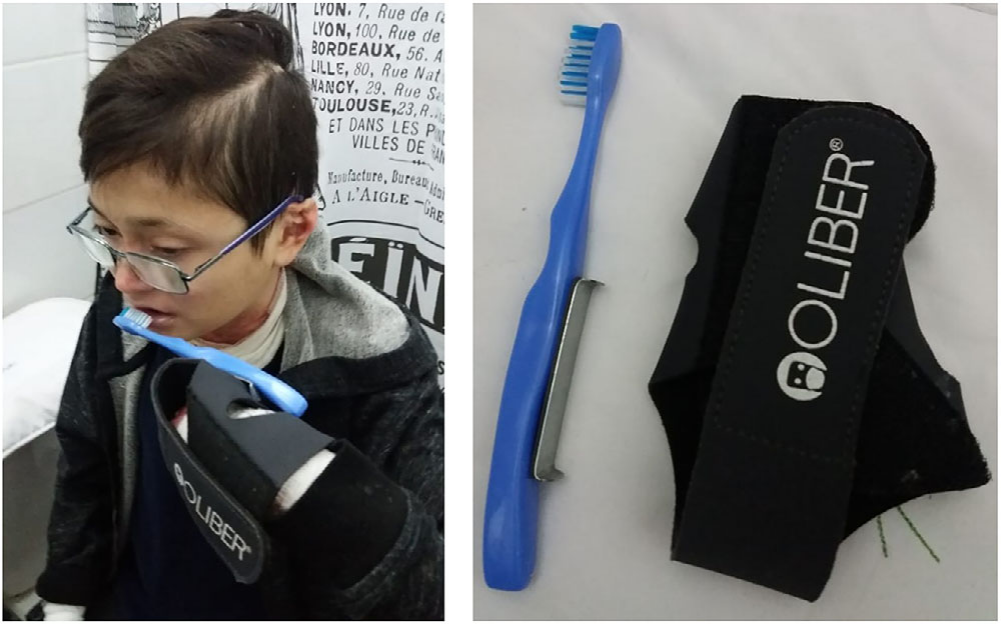
A 19‐year‐old patient with RDEB and complete pseudosyndactyly performing oral hygiene with the Oliber^®^ orthotic


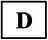
 Rinsing with water during the day, particularly after meals,^10^, ^51^ also helps oral hygiene as it helps remove food debris or sugar deposits particularly in patients with reduced oral function and restricted oral clearance. Oral irrigators can remove food debris, but low water pressure needs to be used to avoid mucosal injury.


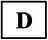
 Disclosing solution or tablets to help identify dental plaque are a useful tool to help patients assess their effectiveness when brushing their teeth. They can be used by all patients with EB (Image 3.12).^45^

**IMAGE 3.12:**
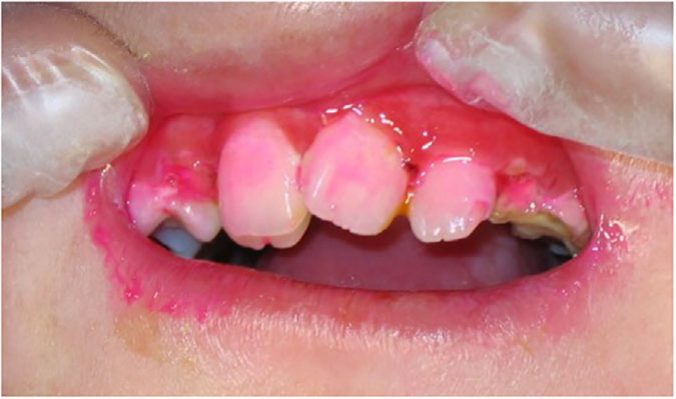
Use of disclosing solution in a patent with RDEB to educate on brushing technique

##### Professional hygiene


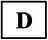
 Gentle and careful ultrasonic scaler and selective polishing techniques can be used in all patients, including severe RDEB.^11^ Hemorrhagic bullae can appear due to vibration on the mucosa. If this happens, they should be drained by piercing the bullae with a sterile needle or by a cut with scissors to avoid lesion expansion due to fluid pressure (more detailed description in Section 3.2.1.iv).

Some clinicians prefer to use hand scaling technique to reduce the need for suction and have a better view and control of the treatment.

#### Adjuvant therapies

##### Chlorhexidine


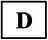
 Chlorhexidine 0.12% has widely been advocated for oral disease prevention in patients with EB.^7^, ^10^, ^11^, ^15^, ^17^, ^45^, ^51^, ^52^ It has shown to be effective for candida while ineffective for caries control.

A variety of application methods have been used, including mouthwashes, swabs, sprays, gels, and topical varnish applications. An example of a preventive treatment protocols is a rinse two times a day for 2 weeks every 3 months.


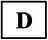
 Alcohol‐free formulations are advised in patients with oral lesions.^10^, ^11^, ^49^

##### Fluoride


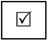
 Caregivers should begin brushing a child's teeth as soon as they come into the mouth. Fluoridated toothpaste should be used with dose appropriate to the age.


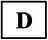
 Topical applications of high‐dose fluoride varnish are suggested every 3 months in patients with high caries risk; or at each dental visit.^7^, ^15^, ^18^, ^35^, ^51^


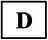
 For children who live in nonfluoridated communities, the importance of daily fluoride supplements has been highlighted.^10^ Dosage should be prescribed according to local regulations, considering age and weight.

Fluoride can also be prescribed as a foam,^14^ gel preparation,^45^ or mouthwash. Gel preparations can be applied with a toothbrush, in a custom made plastic tray^10^ or with cotton rolls. Mouthwash formulations should be alcohol‐free in patients with oral lesions. These 0.05% and 0.2% fluoridated solutions can also be applied topically with a cotton bud on all teeth once a day.^53^ For patients with sensitivity, the use of a nonflavored, nonfoaming, fluoridated toothpaste, as, for example, Oranurse^®^ (found RIS Healthcare, Welwyn, UK) may be useful.

A preventive fluoride regimen should consider these recommendations, together with the best international evidence available on caries prevention strategies.

#### Dietary modifications

Nutritional requirements of patients with EB can be significantly increased, especially in severe subtypes such as RDEB. This regular and targeted intake of high kcal/high protein foods is essential for growth and prevention of malnutrition‐related comorbidities. To meet these high targets, dietary advice may also include the use of high sucrose nutritional supplements, which can be particularly cariogenic. Dietetic advice should be given to minimize the diet's impact on dental health while optimizing the patient's nutritional status, ideally from birth onward.

Leal and coworkers found that patients with RDEB have a similar number of food intakes and similar sugar content than the control group; the main difference was the food consistency, as patients with DEB preferred soft food.^47^

From the patient's perspective: “This is tricky because a lot of kids are tiny, and just desperate to get calories, especially when they are younger and less cooperative. Many also drink high calorie formulas, and others will have g‐tubes. (…) My daughter who is 6.5 (…) drinks a very high calorie medical formula to supplement her diet. She drinks this with the nipples she has been using since she was in the neonatal intensive care unit. We have never made her stop because she needs the calories. She drinks everything else in an age appropriate way, but I think because the formula is pretty thick, she can only tolerate it in this tiny stream. I'm sharing this because I know many other kids with EB who do this. I think dentists should be aware that it occurs, maybe even ask about it. Though I wouldn't expect patients to stop if it is working…because they will prioritize caloric intake.” (R.B., Los Angeles, USA)


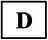
 A dietary caries‐prevention program should be instigated at an early age.^17^, ^50^


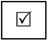
 It is essential that dentists and dietitians/nutritionists collaborate on an appropriate program for each patient, as opposed to giving contradictory advice that may confuse patients and parents/guardians.

#### Fissure sealants and other aids


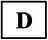
 Sealing fissures and fossae has been recommended, as oral hygiene and other preventive measures can be difficult to perform.^10^, ^18^, ^45^, ^54^


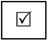
 When moisture control is challenging due to limited cooperation, compromised access, and difficult long‐term follow‐up, glass ionomer can be used as an alternative to resin‐based sealing materials.


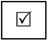
 Other remineralization techniques, such as silver diamine fluoride (SDF) can be used for the noninvasive management of caries lesions in patients with EB (Image 3.14).


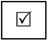
 Xylitol‐containing chewing gum can be used as a preventive strategy for those patients with a high risk of caries. There have been reports where either xylitol chewing gum, or mints have been used in EB patients with no adverse mucosal effects.^35^

**IMAGE 3.13:**
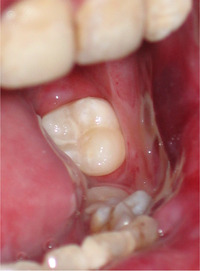
Fissure sealing in a lower second molar of a 16‐year‐old patient with RDEB

**IMAGE 3.14:**
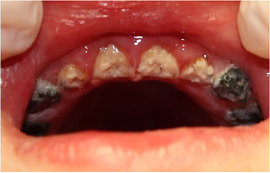
Caries arrest from SDF (note black stain of the teeth) in 6‐year‐old patients with severe RDEB and severe early childhood caries (ECCs)

#### 3.1.9 Microstomia

**IMAGE 3.15:**
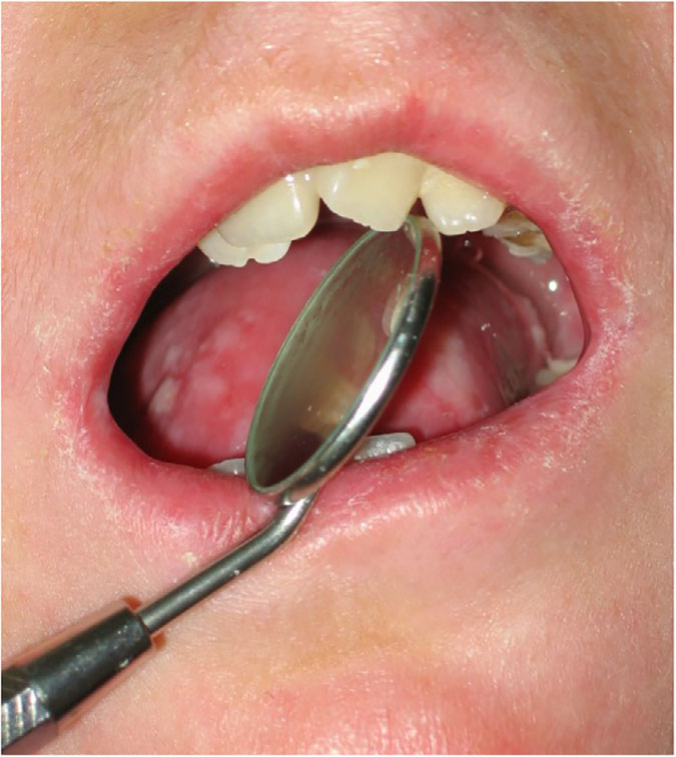
Microstomia makes access to the oral cavity difficult

**IMAGE 3.16:**
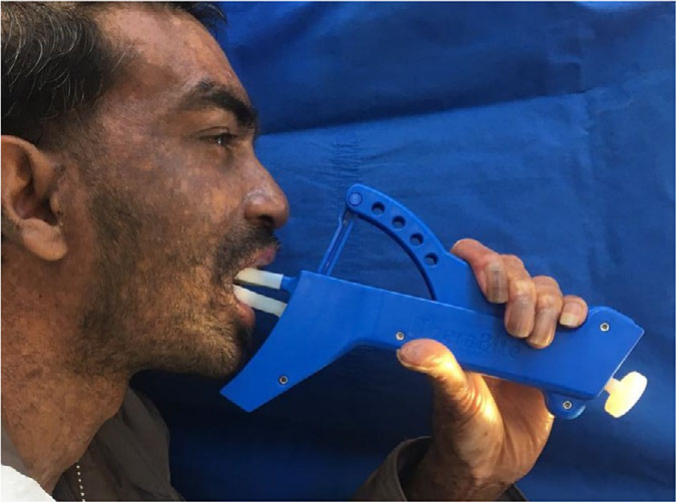
Adult with Kindler EB performing mouth opening exercises with a commercial device: TheraBite^®^ (Atos Medical, Malmö, Sweden)

**IMAGE 3.17:**
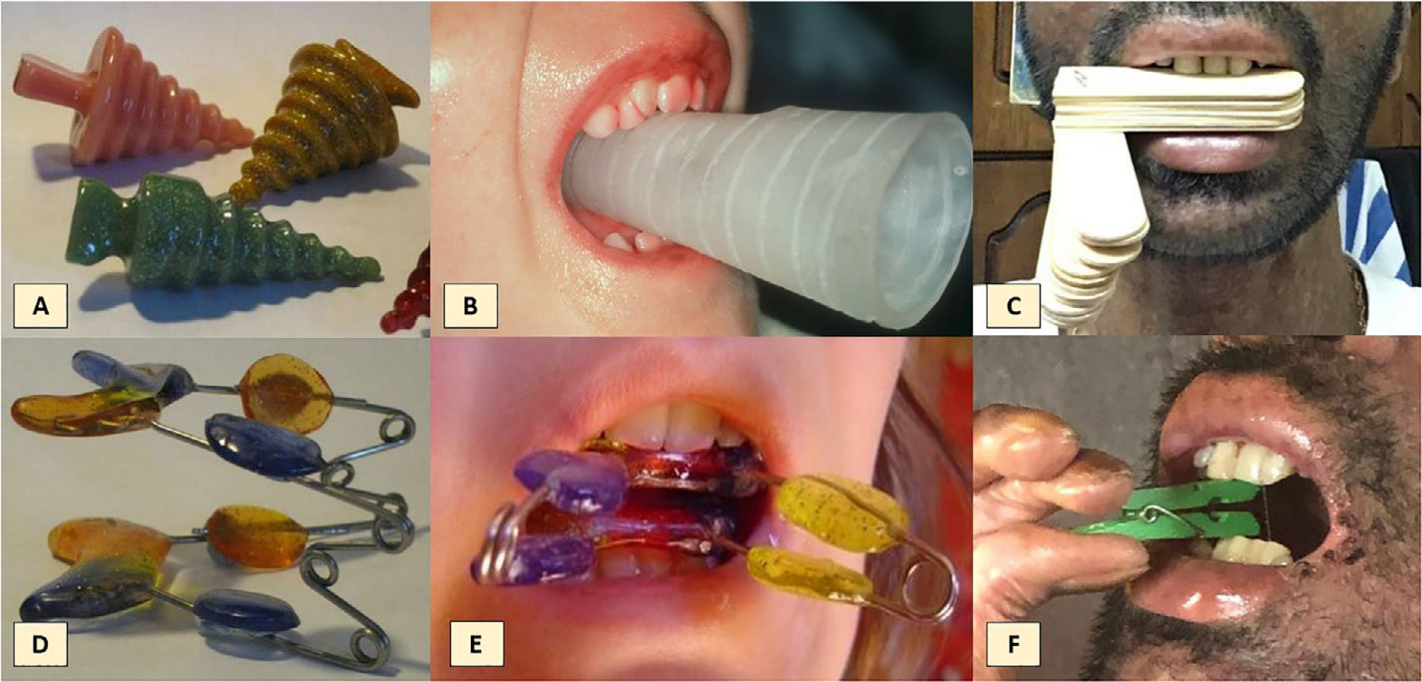
Custom and homemade appliances to perform mouth opening exercises. (A) and (B) Acrylic cones, (C) wooden spatulas, (D) and (E) mouth trainer, and (F) clothes peg to gently exercise opening

Limited mouth opening has been reported as the greatest clinical difficulty for providing dental treatment (Image 3.15)^55^, ^56^ as well as complicating intubation.^57^ In this context, the consulted literature provides no definitive solutions. Slight increments in the maximum oral aperture have been obtained with mechanical techniques. Four techniques have been described (Images 3.16 and 3.17).

The first technique uses resin plugs of progressively increasing caliber. The patient increased his maximal mouth opening from 19 to 23 mm after 10 minutes of exercise, and to 30 mm at the end of a treatment session.^54^ Unfortunately, this parameter returned to the initial values on discontinuing mechanical therapy. Other suggestions include daily exercises with wooden spatulas,^58^ mouth trainer, and threaded acrylic cone (Images 3.16 and 3.17).

A surgical approach to improve microstomia has also been reported, aimed at releasing the scar bands at the commissures and buccal mucosa.^59^, ^60^, ^61^ More research is needed to confirm the long‐term effectiveness of this procedure.


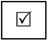
 Patients with severe generalized RDEB should perform daily exercises to improve/maintain a good mouth opening. This can be performed, for example, during dressing changes.


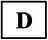
 Performing exercises half an hour before dental treatment helps improve access.^55^


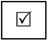
 Improving mouth opening also favors speech and swallowing.


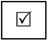
 A surgery to release the buccal intraoral scar bands can aid mouth opening.

#### 3.1.10 Prescriptions


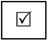
 When prescribing medications for patients with RDEB, it is important to consider that swallowing could be difficult due to esophageal stenosis/strictures or could cause esophageal trauma. Therefore, medications should be in soluble or liquid form. If sugar‐free preparations are not available, parents should be advised of the sugar content and advised ideally to brush or at least rinse the child's teeth with water directly after administration of the medication to reduce the risk of decay.

#### 3.1.11 Review appointments

Frequent recall visits have shown to be useful to maintain dental health in patients with EB.^15^, ^16^, ^62^ There are examples of patients who previously had extensive carious teeth who remained caries free when attending frequent review appointments.^15^, ^62^ On the other hand, clinical cases have been reported, showing that patients who failed to attend the review visits developed several caries within 2 years, despite a preventive program being explained.^11^, ^17^ Moreover, in some patients, even the advice and follow‐up appointments are not enough to avoid the development of dental caries.^47^

As many patients have to travel long distances, review appointments should be scheduled together with other health care appointments. A shared care approach can be considered.


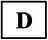
 Frequency of dental review should be scheduled on an individual basis according to the amount of plaque present and risk of caries. Every 3 to 6 months may be sufficient for some patients, for others monthly appointments may be necessary.^5^, ^7^, ^14^, ^16^, ^35^, ^45^, ^48^, ^54^, ^63^


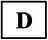
 As the predisposition to develop intraoral SCC increases with age, cancer screening must be considered a very important aspect of the review appointment in patients with RDEB from the third decade and in patients with Kindler EB from the forth decade, regardless of the presence of teeth.^24^, ^28^, ^29^, ^30^, ^31^, ^32^, ^33^, ^34^, ^51^


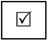
 Any unusual ulcer or persistent white or red lesion should be biopsied to ensure that these do not represent precancerous or cancerous lesion in the mouth.^1^


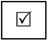
 Monitoring with clinical photographs should also be considered.

To summarize, the review sessions should be aimed at:

- Caries prevention/early diagnosis
- Professional plaque removal
- Topical fluoride application
- Dietary advice
- Review progress or deterioration of patient's oral condition
- Cancer screening

### 3.2 Dental treatment for patients with Inherited Epidermolysis Bullosa

#### 3.2.1 Treatment modifications—Precautions

Even though patients with mild oral involvement do not require many treatment modifications, a careful approach benefits every patient. Patients with the generalized forms of RDEB require the most specific precautions during treatment to minimize soft tissue damage.

##### i. EB simplex (EBS)

**IMAGE 3.18:**
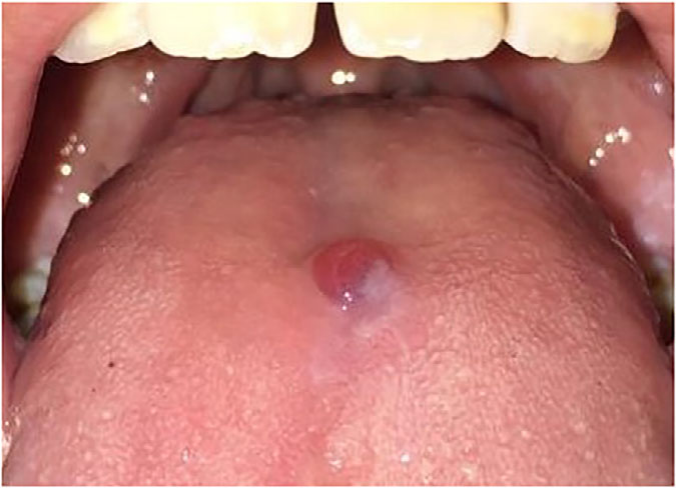
Blister on the tongue of a patient with EBS due to plectin mutation


Clinicians should ask about history of mucosal fragility since manipulation can precipitate lesions in mildly affected patients.^7^ Although this has not happened to the members of the panel; it is recognized that EB is very diverse and that it could happen. Patients with generalized types of EBS can have difficulties, for example, tolerating the suction tip, as well as other dental procedures (Image 3.18).

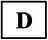
 Most authors agree that routine dental treatment can be provided.^7^, ^54^, ^64^

##### ii. Junctional EB (JEB)

**IMAGE 3.19:**
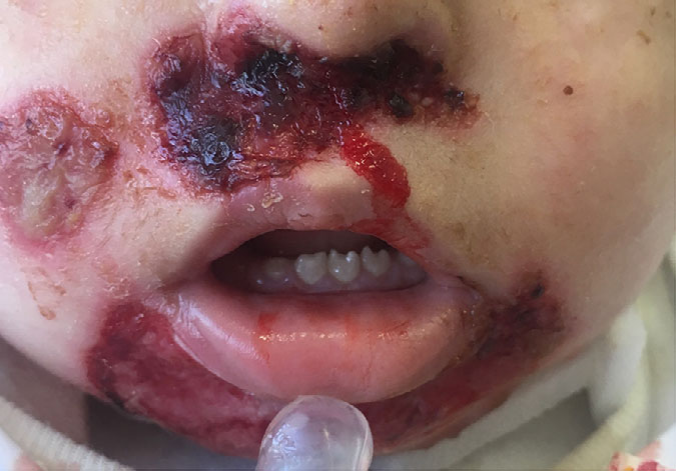
Two‐year‐old patient with JEB and perioral granulation tissue

Mucosal and skin fragility varies considerably between subtypes of JEB and patients. The avoidance of adhesive contact with the skin and careful manipulation is always advised (Image 3.19). Following the suggestions listed in section *Recessive DEB* can be of help for these patients as well.

**IMAGE 3.20:**
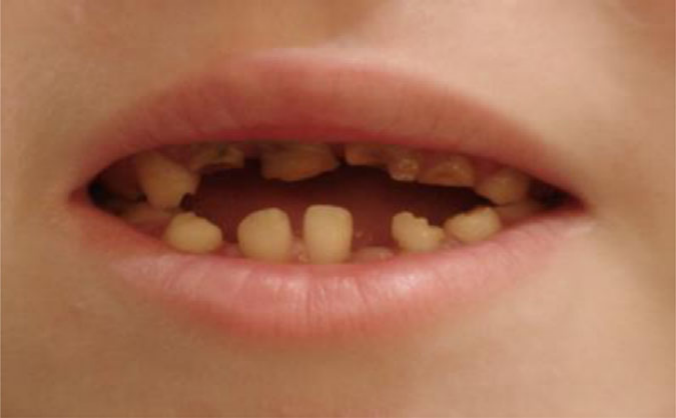
Five‐year‐old patient with JEB: generalized enamel hypoplasia. Perioral granulation tissue has healed without scarring


This group of patients will require a special dental rehabilitation plan, as they present with generalized enamel hypoplasia (Image 3.20).

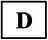
 Mucosal management does not require many modifications;^6^ however, a careful approach is advised as tissue manipulation can produce oral ulceration.


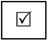
 This group requires an aggressive preventive program and frequent visits to the dentist as they present with generalized enamel hypoplasia (enamel defects on all their teeth), leading to an increased risk of cavities and severe attrition.

If tooth sensitivity is severe or aesthetic considerations are causing behavioral issues, dental rehabilitation should include crowns or veneers from an early age.


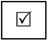
 A periodontist should be included in the multidisciplinary care of patients with JEB if they present gingival overgrowth needing gingivectomy.

##### iii. Dominant DEB (DDEB)


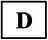
 Patients with DDEB are able to receive routine dental treatment with little or no modifications.^6^, ^24^ Nevertheless, a careful approach is still advised as tissue manipulation can produce oral ulceration.

##### iv. Recessive DEB (RDEB)

Patients with the *severe* and the *intermediate* subtypes of RDEB require several treatment modifications and a careful approach to avoid as much tissue damage as possible. It has been described that on prolonged contact with dental instruments, the epithelium can be sloughed off, exposing areas of erythema.^35^ Management of these patients ideally requires a well‐organized multidisciplinary team approach,^14^, ^37^, ^41^, ^48^, ^63^ with good communication involving case discussion.

**IMAGE 3.21:**
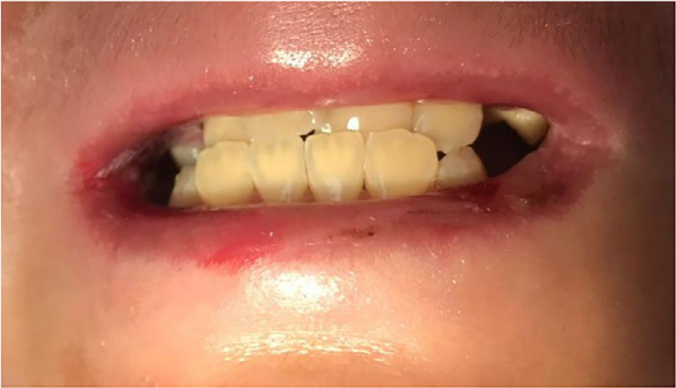
Lips well lubricated with Linovera^®^ during dental treatment in a 9‐year‐old patient with RDEB

**1**. **Lubrication**

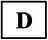
 Lips should always be lubricated with petrolatum, Vaseline^®^ (Unilever), Linovera^®^ (B.Braun), or other appropriate lubricants or emollients before any procedure is performed to reduce adherence and shearing forces that could lead to tissues separation and lesions formation (Image 3.21).^3^, ^7^, ^35^, ^45^, ^50^, ^63^, ^65^

There have been reports suggesting the lubrication of all the instruments. The panel of experts recommends lubricating mainly the back of the intraoral mirror, because it has direct contact with the oral mucosa.

**2**. **Suction tip**

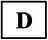
 Bullae formation or epithelial sloughing can occur upon contact with the suction tip.^3^ It is suggested to lean the suction tip or saliva ejector upon hard tissue, ie, on occlusal tooth surface or on a wet cotton roll.^66^


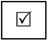
 Avoid use of high vacuum suction as this could cause sloughing of extensive areas of tissue.

**3**. **Bullae**

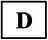
 Blood or fluid‐filled bullae that occur during treatment should be lanced and drained with a sterile needle or by a cut with scissors to avoid lesion expansion due to fluid pressure.^38^, ^39^, ^54^, ^55^, ^67^ The incision should be made at the lowest point of the bullae to allow gravitational drainage allowing the tissue to remain covering the underlying tissue (Images 3.23, 4.9 and 5.2).

**IMAGE 3.22:**
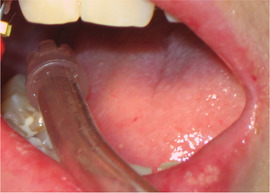
Suction tip leaned on tooth surface to avoid mucosal sloughing

**IMAGE 3.23:**
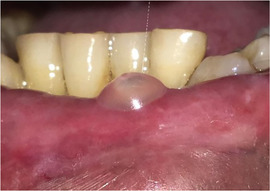
Fluid‐filled bulla that arose on the lower lip of a 33‐year‐old patient during dental treatment. It should be drained immediately

The overlying skin (roof of the blister) should never be removed as it acts like a natural dressing and aids healing, reducing pain, and minimizing the risk of exogenous infection.^39^

As observed in Image 3.35, mucosal sloughing might happen even when all the precautions are followed. Some patients feel that it is of benefit to reposition the broken mucosa, as this reduces the healing time and discomfort.

**IMAGE 3.24:**
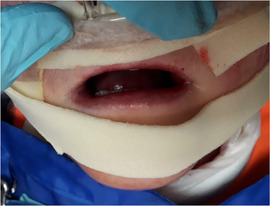
Nonadhesive foam dressing protecting the contact areas

**4**. **Pressure**

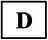
 Extreme care of fragile tissues is important. To handle tissues, a little pressure (compressive forces) can be applied, but no sliding movements (lateral traction or other shear forces) should be used, as these can cause tissue sloughing.^7^, ^11^, ^55^


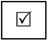
 Patients might prefer to have their extraoral areas covered with nonadherent wound dressings, as, for example, Mepilex Transfer^®^ (Mölnlycke, Gothenburg, Sweden) to reduce the shearing forces and wounds on the lips (Image 3.24).

**5**. **Air‐water syringe**

Occasionally, when using the air syringe to dry the teeth during a dental examination or treatment, an air‐filled bulla can occur. If this happens, they have to be drained.


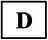
 Air syringe can be used, but should be managed carefully.^11^

**6**. **Instruments**

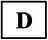
 Due to limited access, it is easier to use pediatric size instruments.^1^


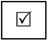
 A laryngeal mirror can also be helpful in patients with severe microstomia. Flat malleable retractors are useful for separating the cheeks, as they spread force over larger area and can protect tissue if having to prepare a tooth for restorative treatment. They come in various widths and are typically available in hospital operating rooms.

**7**. **Isolation**

**IMAGE 3.25:**
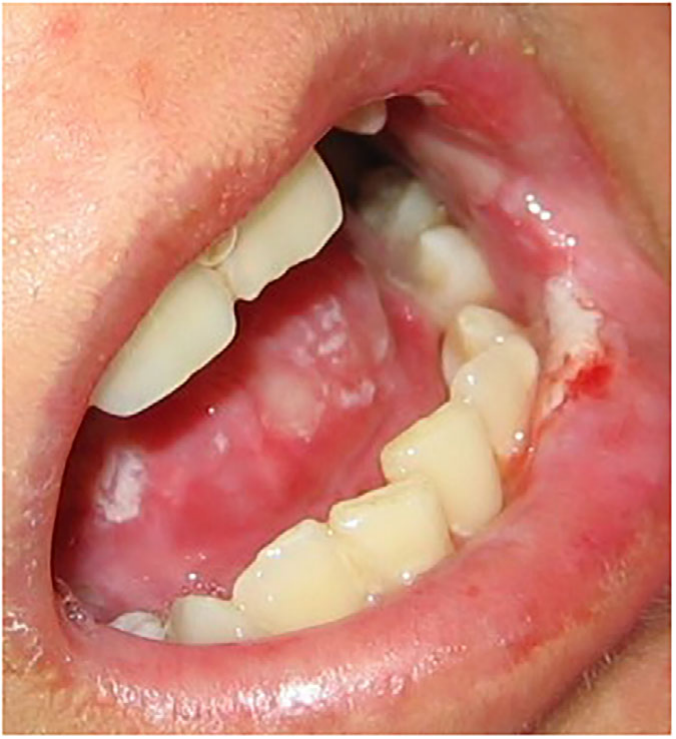
Lip wound caused by the removal of a cotton roll that was not lubricated or soaked with water

Relative isolation:


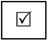
 • Dry cotton rolls stick to the mucosa and can damage the mucosa during removal (Image 3.25). To avoid mucosal damage, cotton rolls can be lubricated with water soluble lubricants such as surgilube^®^ (Novartis, Switzerland) before placing them inside the mouth. When removing them, they must be soaked with water.


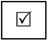
 • Consider reducing the size of the cotton rolls so they can fit in limited spaces. This might be done by splitting cotton rolls in half to fit in the obliterated vestibules.^35^

**IMAGE 3.26:**
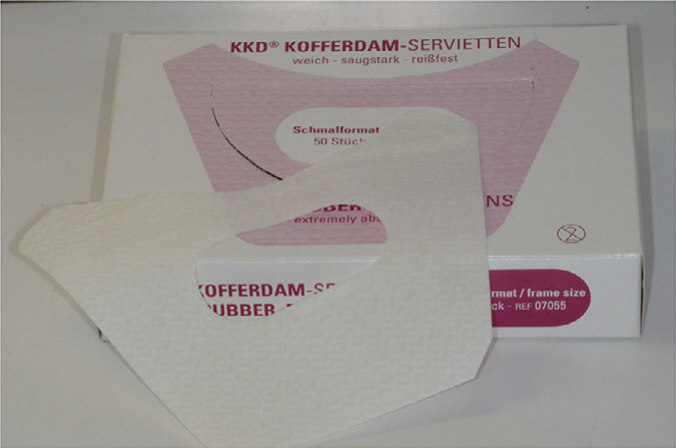
A rubber dam napkin

Complete isolation:


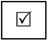
 • Rubber dam can be retained using clamps, but also aided with wooden wedges or Wedjets^®^ (Coltene, Altstätten Switzerland) dental dam stabilizing cord. If choosing clamps, they have to be placed with caution as their placement and positioning could cause blisters or wounds on the lips and cheeks.


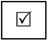
 • The back of the rubber dam can be lubricated to reduce friction with the oral mucosa. Alternatively, a rubber dam napkin can be used (Image 3.26).

Even though these recommendations aid in the placement of a rubber dam to achieve complete tooth isolation, sometimes, it is not possible due to limited mouth opening^35^ or the patient's anxiety.^18^

**8**. **Visual access**

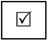
 In severe microstomia, it is easier to separate the lip using the handle of the mirror instead of the mirror itself, or flat malleable retractors as explained before.


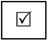
 When possible, consider use of head light.

**9**. **End of session**

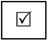
 At the end of every clinical session, it is important to check for fluid‐filled or blood‐filled bullae and drain them. As explained previously, overlying skin should never be removed and mucosa or skin that slough‐off during treatment should be repositioned. It is also important to check and remove any remnants of dental materials in the sublingual space or vestibule, as the patients have ankyloglossia (tongue fused to floor of mouth) and cannot clear the mouth easily. This can be done with a wet cotton roll.

##### v. Kindler EB (KEB)


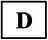
 A careful approach is advised, as mucosal sloughing can occur following dental treatment such as scaling.^68^


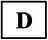
 Periodontal health is the main area of concern for dental therapy as there is a higher prevalence, earlier onset, and faster progression of periodontitis.^69^ Scaling, root planning, and regular dental care are important factors. Regular dental visits are a very important factor to control erosive gingivitis and aggressive periodontitis.^70^

#### 3.2.2 Dental radiography


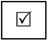
 In most patients with EBS, JEB, DDEB, localized RDEB, and Kindler EB, all diagnostic techniques can be used with no or little technique modification.


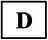
 In patients with *severe and intermediate* form of RDEB routine, periapical technique has been proven to be extremely difficult especially in the posterior area due to microstomia, ankyloglossia, and scarring of the sublingual area. Orthopantomography (panoramic radiograph) is the technique of choice.^1^

**IMAGE 3.27:**
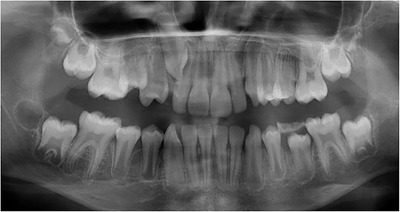
Orthopantomography of a 10‐year‐old patient with severe RDEB


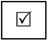
 Other diagnostic techniques that could be considered include:

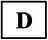
Bitewings using small films.
- Covering the edge of the film with soft periphery wax.
- Some digital panoramic radiographs have extraoral bitewings capabilities making them a good option for patients with limited access.
- Use of cone beam radiography can target certain areas without tissue damage.
- For digital sensors, sharp edges should be avoided. The sleeve should not have any sharp edges. As an alternative, the finger of a glove can be used.
- Occlusal technique can also be used for anterior teeth^14^ or lateral oblique for mandibular posterior teeth.

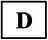
 If periapical radiographs are required in RDEB, care must be taken not to damage the mucosa.^11^ Lubrication of the film packet has been advised to avoid tissue damage.^71^

#### 3.2.3 Restorations

Restorative treatment can be difficult in patients with RDEB due to microstomia, soft‐tissue fragility, vestibular obliteration, and complex anesthetic management.^72^


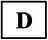
 Caries removal: Some authors prefer to remove carious tissue manually and with low‐speed round burs.^14^


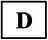
 The use of small‐sized instruments, short‐shaft dental burs, and hand pieces with a small‐sized head are indicated.^45^ Swing oscillating handpieces can also aid to reach difficult angles.


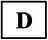
 There are no contraindications to the use of conventional dental materials.^7^, ^73^


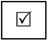
 The restorative material to be used will depend on the possibility of achieving isolation, caries risk, and cultural and economic factors.


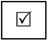
 Consider minimally invasive dentistry (MID) techniques, such as SDF or atraumatic restorative technique (ART).^18^, ^74^


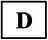
 The use of stainless steel crowns should be considered.^7^, ^54^


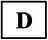
 Restorations and dentures should be carefully adapted and highly polished to lessen the risk of iatrogenic oral mucosal blisters and ulcers.^50^


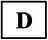
 Iatrogenic blisters can develop after treatment even if all precautions are in place.^54^


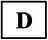
 Soft tissue lesions resulting from restorative treatment typically heal in 1 to 2 weeks and require no specific treatment.^66^


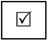
 If required, analgesics can be prescribed.

#### 3.2.4 Endodontics


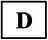
 Root canal (endodontic) treatment (RCT) can be performed in all patients, unless there is no access due to microstomia (limited mouth opening).^66^


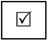
 In patients with severe microstomia access to the pulp chamber might need to be modified. For example, anterior teeth might need buccal access.


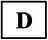
 For determining root canal working length in patients with RDEB and severe microstomia, it is best to use electronic apex locator^14^, ^18^ or, if unavailable, a panoramic radiograph (as periapical radiographs are difficult to take). Cone beam computed tomography (CBCT) may be useful to determine working length prior to endodontic treatment.


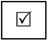
 Rotary instruments used with small‐head endodontic motors have an advantage if there is limited working space. Short files (21 mm) as well as some occlusal reduction of the cusps can allow RCT even in cases where otherwise treatment would not be possible due to lack of space.


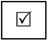
 Concern has been raised regarding the safety of using sodium hypochlorite for root canal decontamination when isolation is challenging due to difficulties in maintaining the rubber dam in position due to the lack of vestibule and space for the clamp. Gingival barrier can be used to facilitate single tooth isolation. (See section *RDEB Isolation technique*.)

#### 3.2.5 Impression taking

**IMAGE 3.28:**
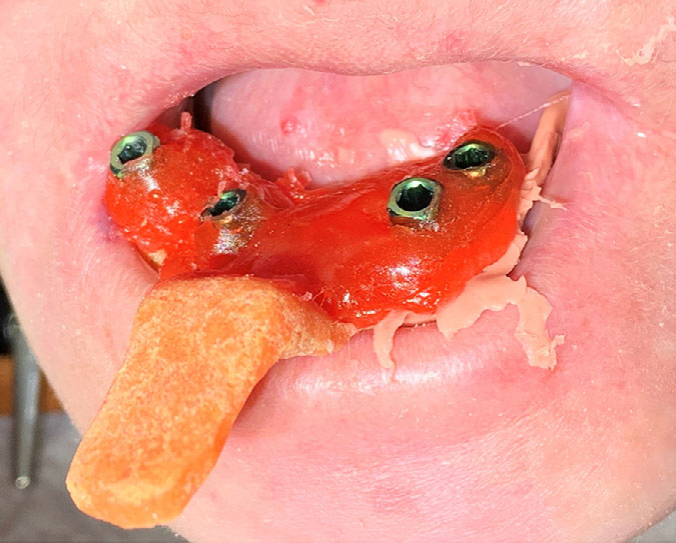
Impression using silicone and a custom‐made tray in a 36‐year‐old patient with severe RDEB

**IMAGE 3.29:**
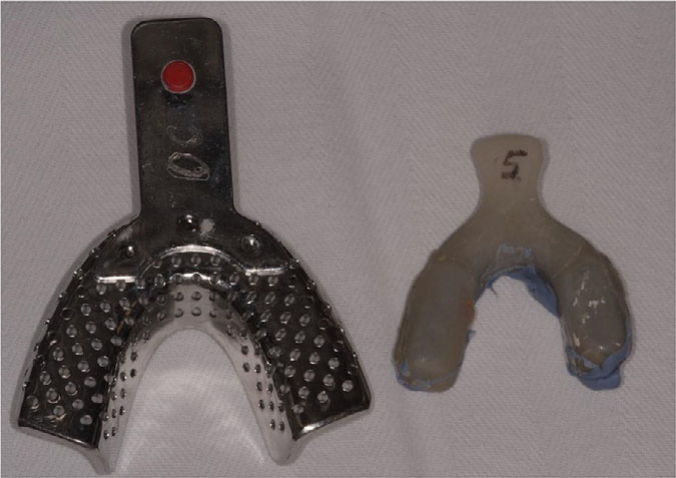
Stock tray and custom‐made acrylic tray (size comparison)

**IMAGE 3.30:**
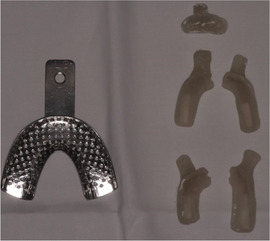
Sectioned individual impression tray

Concern has been raised by dentists and patients regarding the safety of taking impressions in patients with fragile mucosa. No adverse events (ie, mucosal damage) have been published. The main challenge is microstomia, as impression trays might not fit into the mouth.


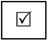
 Impressions should be taken with special care in RDEB.^66^, ^75^, ^76^


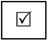
 All type of impression material can be used.


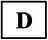
 Microstomia can be a real challenge. As an alternative to stock impression trays, custom‐made acrylic trays or specially cut topical gel application trays have been proposed^50^, ^77^ (Images 3.28, 3.29, and 3.30).

**IMAGE 3.31:**
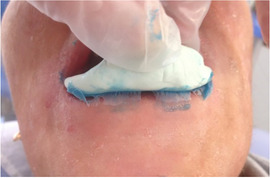
Impression using the silicone putty as a tray

A suggestion for designing the custom‐made tray is to take a first impression with high‐viscosity silicone putty and to construct the custom‐made tray on a second step. Alternatively, silicone putty can act as a custom‐made tray with low‐viscosity silicone to capture the details of the preparation (Image 3.31).

It is very important not to introduce too much silicone into the oral cavity, since in the polymerized state, it can be difficult to remove.


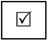
 If the cervical margin is subgingival, a gingivectomy may be needed. For information on this matter, consult the gingivectomy section.


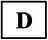
 Intraoral scanners can be a noninvasive harmless impression solution for oral rehabilitation, such as surgical and prosthodontic implant planning and placement in RDEB.^56^


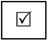
 Stereolithographic models can be used as a diagnostic aid.

#### 3.2.6 Oral rehabilitation

Oral rehabilitation can be fixed or removable depending on the health system and financial possibilities.


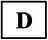
 Whenever possible, fixed rehabilitation is advised.^48^

##### Fixed rehabilitation


**IMAGE 3.32:**
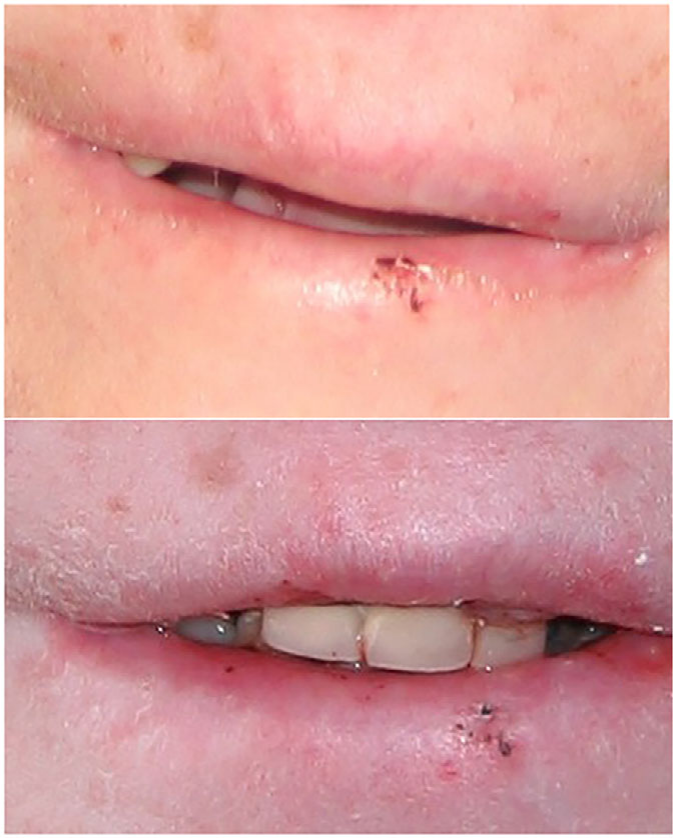
The smile of a 30‐year‐old patient with severe RDEB and severe microsomia before and after fixed crown oral rehabilitation


**IMAGE 3.33:**
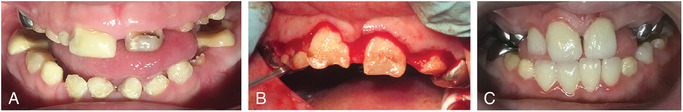
A 9‐year‐old boy with junctional EB and generalized hypoplastic enamel being treated with gingivectomy and complete oral rehabilitation of the hypoplastic teeth in one session under sedation. (A) Before treatment (upper right central incisor has a temporary restoration), (B) first stage of treatment: gingivectomy, and (C) 1 week after the clinical session: all incisors are crowned


**IMAGE 3.34:**
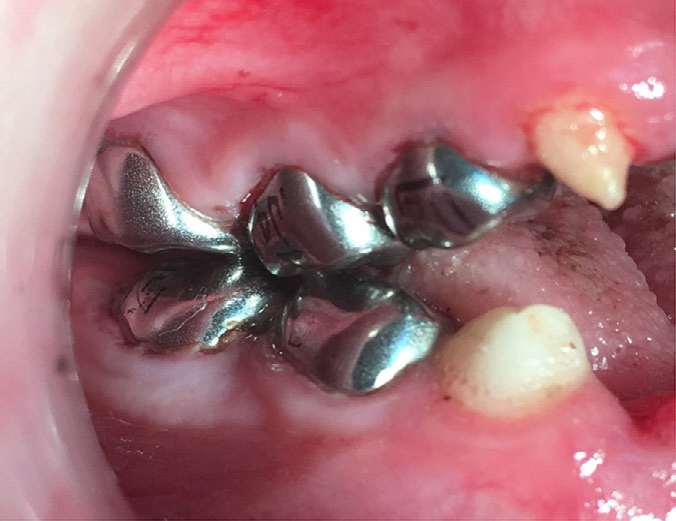
Stainless steel crowns on a 6‐year‐old boy with junctional EB and generalized hypoplastic enamel


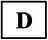
 Successful oral rehabilitation with fixed bridges has been reported in several patients with severe RDEB;^11^, ^50^ improving aesthetics, oral function, and enhancing patients’ confidence (Image 3.32).^50^

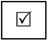
 In patients with generalized enamel hypoplasia, restoration of the entire dentition with full crowns may be necessary. This treatment has to be planned carefully and discussed with the parents and the patient, as it may consist of several stages until full permanent dentition has been established and restored (Image 3.33).^67^, ^78^

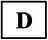
 The use of stainless steel crowns has been reported as a successful approach in children with RDEB and JEB^6^, ^7^, ^54^, ^79^ (Image 3.34).

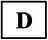
 An anecdotal fixed denture in a 3‐year‐old child with EBS to replace the anterior teeth without causing irritation on the mucosa was reported by Chuang.^48^ The maxillary growth needs to be carefully followed up in this type of rehabilitations.

##### Removable dentures

The tolerance to bear tissue‐supported dentures depends on the degree of mucosal fragility of each EB subtype and patient.


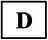
 Reports of patients successfully tolerating removable dentures include patients with EBS, JEB, DDEB, and pretibial RDEB.^6^, ^54^, ^80^, ^81^


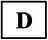
 In selected cases, patients with generalized forms of RDEB can use dentures if the flanges are adapted and the clasps are flat and do not irritate the opposing mucosa.


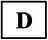
 Overdentures have been described as a practical, economic, nonsurgical treatment option for patients with JEB and generalized hypoplastic enamel who present with failure of eruption.^80^ Careful follow‐up is needed due to the high risk of caries.

##### Implant rehabilitation

As this is an area of high interest for patients and clinicians, a new chapter on this topic has been dedicated to dental implants: Chapter 4: dental implants in patients with recessive dystrophic EB ‐ clinical practice guidelines.

#### 3.2.7 Periodontal treatment


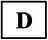
 Periodontal treatment can be performed in all patients with EB. Special care must be taken in patients with RDEB, as there might be substantial bleeding during the procedure.^11^, ^66^ Some authors prefer to use hand scalers.^35^


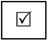
 Gingivectomy can be performed with laser or scalpel. Patients with Kindler EB may need this technique to remove hyperplastic gingival papillae and patients with JEB to manage gingival hyperplasia (Image 3.33B).

#### 3.2.8 Oral surgery

##### Suturing

There has been debate in the literature about the feasibility of suturing after oral surgery in patients with EB.^9^, ^15^, ^55^, ^63^, ^79^, ^82^


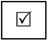
 Sutures can be used safely in all patients with EB but need careful placement and might not always be possible due to the mucosal fragility.

##### Vestibuloplasty

Severe obliteration of the oral vestibule due to scarring can cause difficulties in eating,^59^ performing oral hygiene,^59^ providing dental treatment, and reducing food clearance due to reduced mobility.


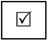
 Periodontal plastic surgery and vestibuloplasty to deepen the vestibule or to restore the alveolar ridge height has been reported in two patients with dominant dystrophic EB (DDEB).^59^, ^60^ The panel has limited but positive experience on this surgery in patients with RDEB. This surgery is recommended when required, ie, when the obliteration affects the patient's quality of life or oral function.


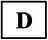
 Inserting a soft acrylic stent extending to the newly established vestibule avoids fusion of the connective tissue layers and allows time for epithelium migration on both surfaces.^60^


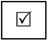
 Alternatively, a periodontal dressing, such as Coe‐Pak^®^ (GC, Tokyo, Japan), can be placed between the alveolar ridge and the lips to avoid fusion during the healing period/during the first days after the surgery.

##### Oral commissures release


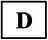
 Only few cases of surgical release of oral commissures scarring have been reported, with limited details on the long‐term success.^83^

##### Biopsy


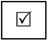
 Biopsies of oral tissues may be required when oral squamous cell carcinoma (OSCC) is suspected. (See Section 3.1.11)

##### Surgical extractions

Contemporary oral health care is targeted at prevention of oral disease, but some patients still require extractions due to severe caries or the need for orthodontic care that involves severe dental crowding. Surgical/difficult extractions should be performed by an experienced dental provider.


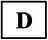
 When planning surgical extractions, especially if multiple extractions are needed, it is advisable to consult the patient's physician as profound anemia could complicate the dental surgery.^41^


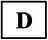
 For multiple extractions, it has been suggested to extract first the anterior teeth (ie, from premolar to premolar) and then the molars to allow optimal access.^41^


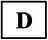
 An atraumatic technique should be used, making firm and safe mucosal incisions to prevent bullae formation.^10^, ^55^


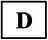
 Hemostasis can be achieved with gentle pressure using gauze packs.^9^, ^79^ These should be wet to avoid tissue adherence. Other hemostatic agents, such as collagen sponges, gelatin sponges and oxidized cellulose can be used safely.

Wound healing process of extraction sockets in patients with this condition remains unaltered. Some authors have reported the extraction of healthy third or even second permanent molars in patients with severe RDEB to improve or facilitate oral hygiene.^4^, ^84^ There is controversy among different authors about this intervention. Severe tooth crowding,^12^, ^54^, ^85^ reduced alveolar arches secondary to growth retardation,^49^, ^86^ and severe microstomia^3^, ^15^, ^54^, ^55^, ^82^, ^87^, ^88^, ^89^ are described in patients with generalized severe RDEB, which would justify preventive extractions. However, nowadays most patients receive dietetic advice that optimizes nutrition and growth. They receive orthodontic treatment (serial extractions) and are advised on exercises to improve microstomia. Therefore, preventive extractions of permanent molars need to be assessed very carefully on an individual basis.

###### Perioperative complications

**IMAGE 3.35:**
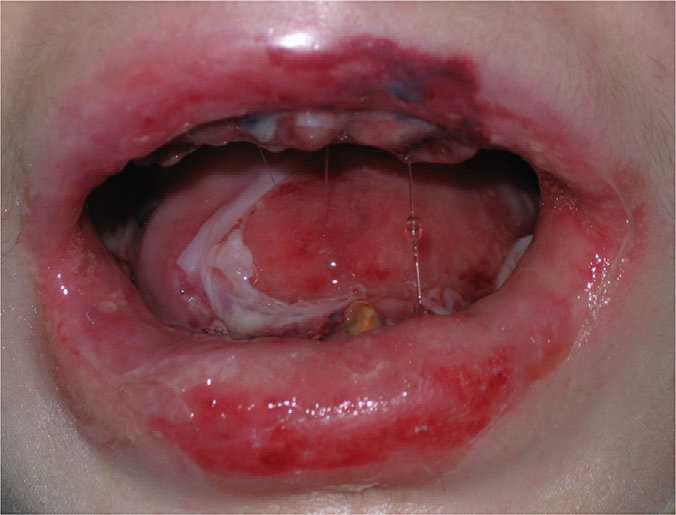
Bullae, ulcers, and mucosal sloughing after surgical extractions

Despite attempts to use as gentle manipulation as possible and follow the precautions described above, mucosal sloughing and blister formation has been reported after almost every surgical extraction in patients with severe RDEB (Image 3.35).^3^, ^9^, ^41^, ^54^, ^79^ Blisters can arise at the angles of the mouth, lips, vestibule, tongue, and any sites of manipulation; some measuring up to 4 cm × 3 cm.^3^, ^41^ In some instances, they might only be noticed by the patient or carer only on the second postoperative day.^9^ As described in Section 3.2.1, tissue that has sloughed off should ideally be repositioned. If this is not possible, it should either be left of cut with scissors, but never torn, as this would increase the size of the wound.

###### Postoperative complications

Despite the potential for extensive mucosal damage during surgery, postoperative complications are rare.^9^, ^41^, ^90^ Healing of the oral tissues occurs gradually after 1 to 2 weeks.^17^, ^53^, ^79^ Healing of the alveolar sockets seems to be uneventful.^9^, ^62^ Nevertheless, scarring of the oral commissure or wounded areas can be accentuated after surgery.^3^, ^9^ The patient should be advised to perform mouth opening, lip, and tongue movement exercises during the healing process to maintain oral functioning.

The use of postoperative antibiotics will depend on each individual case, and there is no particular need because of the patient's EB condition.

###### Osseointegrated implants

As osseointegrated implants are an area of growing interest for people with EB and their clinicians, a separate *“Guideline on Dental Implants in Patients with Recessive Dystrophic Epidermolysis Bullosa”* has been developed. Only key elements are presented.

Successful rehabilitation using dental implants has been reported in patients with generalized RDEB, intermediate JEB, and inversa‐RDEB.^7^, ^55^, ^65^, ^87^ One‐year osseointegration success rate, based on 217 implants, is 98.6%. Peri‐implant mucosa remained in good condition in all patients.^8^, ^56^, ^65^ It has been reported that after rehabilitation, patients improved their ability to chew, swallow, and their quality of life.^55^, ^65^, ^75^, ^76^

###### Sialolithiasis of submandibular gland

Successful surgical removal of a sialolith using local anesthesia has been reported once. The intervention was extremely challenging due to the patients microstomia and ankyloglossia.^36^ Another patient who has had the same issues reported successful stone dislodgment after sucking lemon to increase salivary flow.

#### 3.2.9 Orthodontics


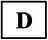
 In patients with *less severe mucosal fragility* and oral scars (EBS, JEB, DDEB, KEB):

- Orthodontic treatment only requires minor modifications.^7^ Patients with generalized and severe forms of EBS and JEB, however, can have more mucosal fragility requiring the precautions indicated below.

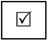
 In patients *with severe mucosal fragility* and oral scars (RDEB):

Serial extractions are strongly recommended in patients with severe RDEB to prevent dental crowding, as this contributes to high caries risk and periodontal disease.

**IMAGE 3.36:**
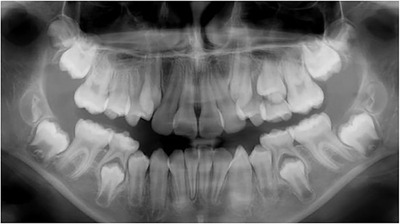
Eleven‐year‐old patient with severe RDEB. Serial extractions of the first upper premolars were planned to allow eruption of the canines

**IMAGE 3.37:**
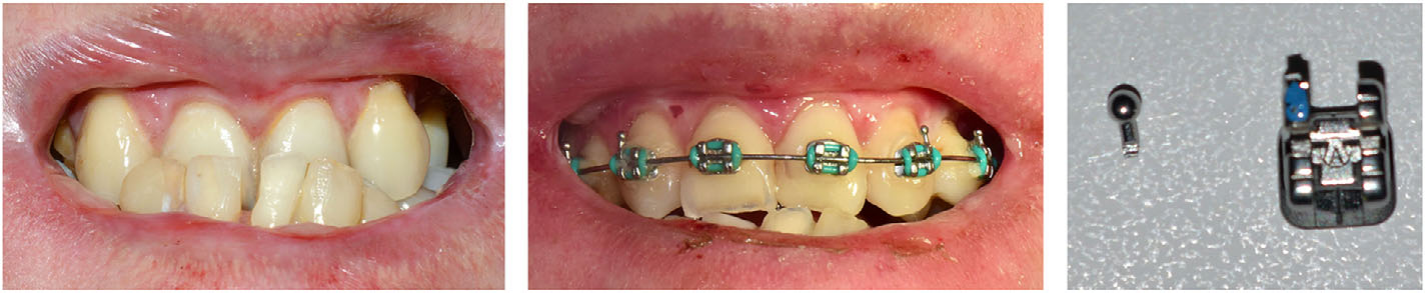
Twenty‐three‐year‐old patient with severe RDEB: (A) Before and (B) during orthodontic treatment. (C) The hook of the canine bracket had to be removed as it caused trauma to the lip. Note changes in upper lip, as the patient also received vestibuloplasty during the same period


- The aim of orthodontics in severe RDEB should be to avoid tooth crowding and obtain tooth alignment.
- Serial extractions should be performed at the appropriate stage of dental development (Image 3.36).
- A risk‐benefit analysis should be performed on an individual basis to avoid the need for repeated general anesthetic for serial extractions. These procedures should ideally be done with behavioral support techniques and local anesthesia.
- When using fixed orthodontic appliances, microstomia and vestibule obliteration might affect the treatment plan. Most patients tolerate braces surprisingly well, although small modifications such as removing the hooks might be necessary (Image 3.37). Placement and bonding of posterior brackets might be challenging and not possible in all patients.

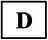
 To prevent lesions on the soft tissues orthodontic wax/relief wax can be applied on the brackets.^84^

Even though some authors have stated that orthodontic treatment can only be performed in mild forms of EB,^77^ fixed orthodontics have successfully been able to achieve tooth movement in order to: (a) correct a one tooth cross bite, (b) close diastema, and (c) align the anterior teeth in patients with severe RDEB.

A tooth‐borne removable appliances and clear aligners may also be possible treatment options.

##### Images

We would like to acknowledge the support of patients, clinicians, and researches from different clinical centers globally for collaborating by providing images for the present guideline. Written informed consent has been obtained for all images where patients could be recognized.

A special acknowledgment to: Image 5: Dr. Daniela Adorno and Dr. Gina Pennacchiotti, Oral Pathologist, University of Chile; Image 8 and 26: Dr. Reinhard Schilke, Hochschule Hannover, Germany; Image 17 B: Dr. María Concepción Serrano, Spain; Image 17 D and E: Sabine Daby, DEBRA Germany; Image 28: Dr. Antonio Olivares, Chile; Images 29 and 30: Dr. Mark Antal, University of Szeged, Hungary; Image 37: Dr. Sebastián Véliz, Orthodontist, Universidad de Chile.

REFERENCES1

Krämer
SM
, 
Serrano
MC
, 
Zillmann
G
, et al. Oral health care for patients with epidermolysis bullosa ‐ best clinical practice guidelines. Int J Paediatr Dent. 2012;22(Suppl. 1):1‐35.2293790810.1111/j.1365-263X.2012.01247.x2
Scottish Intercollegiate Guidelines Network.
, 
Harbour
RT
, 
Forsyth
L
. SIGN 50: A Guideline Developer's Handbook. Ediburgh, Scotland: Scottish Intercollegiate Guidelines Network; 2008:102.3

Stavropoulos
F
, 
Abramowicz
S
. Management of the oral surgery patient diagnosed with Epidermolysis Bullosa: report of 3 cases and review of the literature. J Oral Maxillofac Surg. 2008;66(3):554‐559.1828039410.1016/j.joms.2007.06.6724

Skogedal, N.
, 
Saltnes, S.
, 
Storhaug
K
. Recessive dystrophic epidermolysis bullosa (RDEB). Caries prevention and preventive extractions of molars In Clinical Presentation of 3 Cases. 2008.5

Louloudiadis
AK
, 
Louloudiadis
KA
. Case report: dystrophic epidermolysis bullosa: dental management and oral health promotion. Eur Arch Paediatr Dent. 2009;10(1):42‐45.1925452710.1007/BF032626676

Crawford
EG
, 
Burkes
EJ
, 
Briggaman
RA
. Hereditary epidermolysis bullosa: oral manifestations and dental therapy. Oral Surg Oral Med Oral Pathol. 1976;42(4):490‐500.106754810.1016/0030-4220(76)90296-67

Wright
JT
. Oral manifestations of epidermolysis bullosa In: FineJ‐D, ed. Epidermolysis Bullosa Clinical, Epidemiologic, and Laboratory Advances and the Findings of the National Epidermolysis Bullosa Registry. Baltimore: The Johns Hopkins University Press; 1999:236‐256.8

Larrazabal‐Morón
C
, 
Boronat‐López
A
, 
Peñarrocha‐Diago
M
, 
Peñarrocha‐Diago
M
. Oral rehabilitation with bone graft and simultaneous dental implants in a patient with epidermolysis bullosa: a clinical case report. J Oral Maxillofac Surg. 2009;67(7):1499‐1502.1953142410.1016/j.joms.2009.03.0349

Boyer
HE
, 
Owens
RH
. Epidermolysis bullosa: a rare disease of dental interest. Review of the literature and report of a case. Oral Surg Oral Med Oral Pathol. 1961;14:1170‐1177.1387218210.1016/0030-4220(61)90205-510

Nowak
AJ
. Oropharyngeal lesions and their management in epidermolysis bullosa. Arch Dermatol. 1988;124(5):742‐745.336499611

Olsen
CB
, 
Bourke
LF
. Recessive dystrophic epidermolysis bullosa. Two case reports with 20‐year follow‐up. Aust Dent J. 1997;42(1):1‐7.907863810.1111/j.1834-7819.1997.tb00087.x12

De Benedittis
M
, 
Petruzzi
M
, 
Favia
G
, 
Serpico
R
. Oro‐dental manifestations in Hallopeau‐Siemens‐type recessive dystrophic epidermolysis bullosa. Clin Exp Dermatol. 2004;29(2):128‐132.1498726510.1111/j.1365-2230.2004.01485.x13

Cagirankaya
LB
, 
Hatipoglu
MG
, 
Hatipoglu
H
. Localized epidermolysis bullosa simplex with generalized enamel hypoplasia in a child. Pediatr Dermatol. 2006;23(2):167‐168.1665022910.1111/j.1525-1470.2006.00206.x14

Kummer
TR
, 
Nagano
HCM
, 
Tavares
SS
, 
Dos Santos
BZ
, 
Miranda
C
. Oral manifestations and challenges in dental treatment of epidermolysis bullosa dystrophica. J Dent Child (Chic). 2013;80(2):97‐100.2401129915

Azrak
B
, 
Kaevel
K
, 
Hofmann
L
, 
Gleissner
C
, 
Willershausen
B
. Dystrophic epidermolysis bullosa: oral findings and problems. Spec Care Dentist. 2006;26(3):111‐115.1677418810.1111/j.1754-4505.2006.tb01433.x16

Momeni
A
, 
Pieper
K
. Junctional epidermolysis bullosa: a case report. Int J Paediatr Dent. 2005;15(2):146‐150.1579037510.1111/j.1365-263X.2005.00622.x17

Silva
LCP
, 
Cruz
RA
, 
Abou‐Id
LR
, 
Brini
LNB
, 
Moreira
LS
. Clinical evaluation of patients with epidermolysis bullosa: review of the literature and case reports. Spec Care Dentist. 2004;24(1):22‐27.1515705610.1111/j.1754-4505.2004.tb01675.x18

Korolenkova
MV
. [Dental treatment in children with dystrophic form of epidermolysis bullosa]. Stomatologiia (Mosk). 2015;94(2):34‐36.2614547510.17116/stomat201594234-3619

Fortuna
G
, 
Chainani‐Wu
N
, 
Lozada‐Nur
F
, et al. Epidermolysis Bullosa Oropharyngeal Severity (EBOS) score: a multicenter development and reliability assessment. J Am Acad Dermatol. 2013;68(1):83‐92.2257515810.1016/j.jaad.2012.04.00920

Fortuna
G
, 
Aria
M
, 
Cepeda‐Valdes
R
, et al. Clinical features of gingival lesions in patients with dystrophic epidermolysis bullosa: a cross‐sectional study. Aust Dent J. 2015;60(1):18‐23.2572127510.1111/adj.1226421

Fortuna
G
, 
Lozada‐Nur
F
, 
Pollio
A
, et al. Patterns of oral mucosa lesions in patients with epidermolysis bullosa: comparison and agreement between oral medicine and dermatology. J Oral Pathol Med. 2013;42(10):733‐740.2377283210.1111/jop.1209422

Fortuna
G
, 
Aria
M
, 
Cepeda‐Valdes
R
, 
Salas‐Alanís
JC
. Evaluation of internal consistency of the epidermolysis bullosa oropharyngeal severity score (EBOS). Acta Odontol Scand. 2015;73(2):156‐160.2559817210.3109/00016357.2014.93146023

Fortuna
G
, 
Pollio
A
, 
Aria
M
, et al. Genotype–oropharyngeal phenotype correlation in Mexican patients with dystrophic epidermolysis bullosa. Int J Oral Maxillofac Surg. 2014;43(4):491‐497.2421083510.1016/j.ijom.2013.10.00424

Wright
JT
, 
Fine
JD
, 
Johnson
LB
. Oral soft tissues in hereditary epidermolysis bullosa. Oral Surg Oral Med Oral Pathol. 1991;71(4):440‐446.205232910.1016/0030-4220(91)90426-d25

Reed
WB
, 
College
J
, 
Francis
MJO
, et al. Epidermolysis bullosa dystrophica with epidermal neoplasms. Arch Dermatol. 1974;110(6):894‐902.461327926

Martinez
L
, 
Goodman
P
, 
Crow
WN
. Squamous cell carcinoma of the maxillary sinus and palate in epidermolysis bullosa: CT demonstration. J Comput Assist Tomogr. 16(2):317‐9.154503510.1097/00004728-199203000-0002727

Schiller
F
. Tongue carcinoma in epidermolysis bullosa dystrophical. Arch Klin Exp Dermatol. 1960;209:643‐651.1444276328

Lotem
M
, 
Raben
M
, 
Zeltser
R
, et al. Kindler syndrome complicated by squamous cell carcinoma of the hard palate: successful treatment with high‐dose radiation therapy and granulocyte‐macrophage colony‐stimulating factor. Br J Dermatol. 2001;144(6):1284‐1286.1142207110.1046/j.1365-2133.2001.04262.x29

Saleva
M
, 
Has
C
, 
He
Y
, et al. Natural history of kindler syndrome and propensity for skin cancer ‐ case report and literature review [Internet]. JDDG ‐ J German Soc Dermatol. 2018;16:338‐341. 10.1111/ddg.13435
2938427130

Caldeira
A
, 
Trinca
WC
, 
Flores
TP
, et al. A Kindler syndrome‐associated squamous cell carcinoma treated with radiotherapy. Reports Pract Oncol Radiother. 2016;21(6):532‐536.10.1016/j.rpor.2016.07.004PMC50218472766056031

Souldi
H
, 
Bajja
MY
, 
Mahtar
M
. Kindler syndrome complicated by invasive squamous cell carcinoma of the palate. Eur Ann Otorhinolaryngol Head Neck Dis. 2018;135(1):59‐61.2864195710.1016/j.anorl.2017.05.00332

Has
C
, 
Wessagowit
V
, 
Pascucci
M
, et al. Molecular basis of Kindler syndrome in Italy: novel and recurrent Alu/Alu recombination, splice site, nonsense, and frameshift mutations in the KIND1 gene. J Invest Dermatol. 2006;126(8):1776‐1783.1667595910.1038/sj.jid.570033933

Ashton
GHS
, 
McLean
WHI
, 
South
AP
, et al. Recurrent mutations in Kindlin‐1, a novel keratinocyte focal contact protein, in the autosomal recessive skin fragility and photosensitivity disorder, Kindler syndrome. J Invest Dermatol. 2004;122(1):78‐83.1496209310.1046/j.0022-202X.2003.22136.x34

Lanschuetzer
CM
, 
Muss
WH
, 
Emberger
M
, et al. Characteristic immunohistochemical and ultrastructural findings indicate that Kindler's syndrome is an apoptotic skin disorder. J Cutan Pathol. 2003;30(9):553‐560.1450740310.1034/j.1600-0560.2003.00119.x35

Puliyel
D
, 
Chiu
C
, 
Habibian
M
. Restorative and periodontal challenges in adults with dystrophic epidermolysis bullosa. J Calif Dent Assoc. 2014;42(5):313‐318.2508734936

Primo
BT
, 
da Costa
DJ
, 
Stringhini
DJ
, et al. Sialolithiasis in the duct of submandibular gland: a case report in patient with epidermolysis bullosa. J Contemp Dent Pract. 2013;14(2):339‐344.2381167010.5005/jp-journals-10024-132437

Gonzalez
ME
. Evaluation and treatment of the newborn with epidermolysis bullosa. Semin Perinatol. 2013;37(1):32‐39.2341976110.1053/j.semperi.2012.11.00438

Denyer
J
, 
Pillay
E
, 
Clapham
J
. Best Practice Guidelines Skin and wound care in Epidermolysis Bullosa [Internet]. London; 2017
www.woundsinternational.com
39

Pope
E
, 
Lara‐Corrales
I
, 
Mellerio
J
, et al. A consensus approach to wound care in epidermolysis bullosa. J Am Acad Dermatol. 2012;67(5):904‐917.2238703510.1016/j.jaad.2012.01.016PMC365540340

Ames
WA
, 
Mayou
BJ
, 
Williams
KN
, 
Williams
K
. Anaesthetic management of epidermolysis bullosa. Br J Anaesth. 1999;82(5):746‐751.1053655410.1093/bja/82.5.74641

Album
MM
, 
Gaisin
A
, 
Lee
KWT
, et al. Epidermolysis bullosa dystrophica polydysplastica: a case of anesthetic management in oral surgery. Oral Surg Oral Med Oral Pathol. 1977;43(6):859‐872.26667910.1016/0030-4220(77)90078-042

Lin
Y‐C
, 
Golianu
B
. Anesthesia and pain management for pediatric patients with dystrophic epidermolysis bullosa. J Clin Anesth. 2006;18(4):268‐271.1679742810.1016/j.jclinane.2005.11.00443

Marini
I
, 
Vecchiet
F
. Sucralfate: a help during oral management in patients with epidermolysis bullosa. J Periodontol. 2001;72(5):691‐695.1139440710.1902/jop.2001.72.5.69144

Sindici
E
, 
Astesano
S
, 
Fazio
L
, et al. Treatment of oral lesions in dystrophic epidermolysis bullosa: a case series of cord blood platelet gel and low‐level laser therapy. Acta Derm Venereol. 2017;97(3):383‐384.2753503710.2340/00015555-251245

Torres
CP
, 
Gomes‐Silva
JM
, 
Mellara
TS
, 
Carvalho
LP
, 
Borsatto
MC
. Dental care management in a child with recessive dystrophic epidermolysis bullosa. Braz Dent J. 2011;22(6):511‐516.2218964810.1590/s0103-6440201100060001246

Sharma
S
, 
Bedi
S
. Dystrophic epidermolysis bullosa associated with non‐syndromic hypodontia. Indian Dermatol Online J. 2013;4(4):296‐299.2435000910.4103/2229-5178.120644PMC385389447

Leal
SC
, 
Lia
EN
, 
Amorim
R
, et al. Higher dental caries prevalence and its association with dietary habits and physical limitation in Epidermolysis Bullosa patients: a case control study. J Contemp Dent Pract. 2016;17(3):211‐216.2720720010.5005/jp-journals-10024-182948

Chuang
LC
, 
Hsu
CL
, 
Lin
SY
. A fixed denture for a child with epidermolysis bullosa simplex. Eur J Paediatr Dent. 2015;16(4):315‐318.2663725749

Wright
JT
, 
Fine
JD
, 
Johnson
L
. Hereditary epidermolysis bullosa: oral manifestations and dental management. Pediatr Dent. 1993;15(4):242‐248.824789750

Siqueira
MA
, 
de Souza Silva
J
, 
Silva
FWG de P
, et al. Dental treatment in a patient with epidermolysis bullosa. Spec Care Dent. 2008;28(3):92‐95.10.1111/j.1754-4505.2008.00012.x1848965551

Wright
JT
, 
Fine
JD
, 
Johnson
L
. Dental caries risk in hereditary epidermolysis bullosa. Pediatr Dent. 1994;16(6):427‐432.785495052

Harris
JC
, 
Bryan
RA
, 
Lucas
VS
, 
Roberts
GJ
. Dental disease and caries related microflora in children with dystrophic epidermolysis bullosa. Pediatr Dent. 2001;23(5):438‐443.1169917253

Oliveira
TM
, 
Sakai
VT
, 
Candido
LA
, 
Silva
SMB
, 
Machado
MAAM
. Clinical management for epidermolysis bullosa dystrophica. J Appl Oral Sci. 2008;16(1):81‐85.1908929510.1590/S1678-77572008000100016PMC432728654

Serrano Martínez
C
, 
Silvestre Donat
FJ
, 
Bagán Sebastián
JV
, 
Peñarrocha Diago
M
, 
Alió Sanz
JJ
. Epidermólisis ampollosa hereditaria a propósito del manejo odontológico de tres casos clínicos. Med oral. 2001;6(1):48‐56.1148813155

Peñarrocha‐Diago
M
, 
Serrano
C
, 
Sanchis
JM
, 
Silvestre
FJ
, 
Bagán
JV
. Placement of endosseous implants in patients with oral epidermolysis bullosa. Oral Surg Oral Med Oral Pathol Oral Radiol Endodontol. 2000;90(5):587‐590.10.1067/moe.2000.1104381107738156

Oliveira
MA
, 
Ortega
KL
, 
Martins
FM
, 
Maluf
PSZ
, 
Magalhes
MG
. Recessive dystrophic epidermolysis bullosa”oral rehabilitation using stereolithography and immediate endosseous implants. Spec Care Dent. 2010;30(1):23‐26.10.1111/j.1754-4505.2009.00117.x2005107157

George
M
, 
Martinez
AE
, 
Mellerio
JE
, 
Nandi
R
. Maxillary alveolar process fracture complicating intubation in a patient with epidermolysis bullosa. Pediatr Anesth. 2009;19(7):706‐707.10.1111/j.1460-9592.2009.02995.x1963812258

Lanschuetzer
CM
, 
Fine
J‐D
, 
Laimer
M
, et al. General Aspects In: FineJ‐D, HintnerH, eds. Life with Epidermolysis Bullosa (EB). Vienna: Springer Vienna; 2009: 1‐95. 10.1007/978-3-211-79271-1_1
59

Buduneli
E
, 
Ilgenli
T
, 
Buduneli
N
, 
Ozdemir
F
. Acellular dermal matrix allograft used to gain attached gingiva in a case of epidermolysis bullosa. J Clin Periodontol. 2003;30(11):1011‐1015.1476112510.1034/j.1600-051x.2003.00398.x60

Brain
JH
, 
Paul
BF
, 
Assad
DA
. Periodontal plastic surgery in a dystrophic epidermolysis bullosa patient: review and case report. J Periodontol. 1999;70(11):1392‐1396.1058850410.1902/jop.1999.70.11.139261

Ciccarelli
AO
, 
Rothaus
KO
, 
Carter
DM
, 
Lin
AN
. Plastic and reconstructive surgery in epidermolysis bullosa: Clinical experience with 110 procedures in 25 patients. Ann Plast Surg. 1995;35(3):254‐261.750351810.1097/00000637-199509000-0000662

Finke
C
, 
Haas
N
, 
Czarnetzki
BM
. [Value of dental treatment in interdisciplinary management of a child with epidermolysis bullosa dystrophica hereditaria (Hallopeau‐Siemens)]. Hautarzt. 1996;47(4):307‐310.865531810.1007/s00105005042163

Harel‐Raviv
M
, 
Bernier
S
, 
Raviv
E
, 
Gornitsky
M
. Oral epidermolysis bullosa in adults. Spec Care Dentist. 1995;15(4):144‐148.900291710.1111/j.1754-4505.1995.tb00502.x64

Hochberg
MS
, 
Vazquez‐Santiago
IA
, 
Sher
M
. Epidermolysis bullosa. A case report. Oral Surg Oral Med Oral Pathol. 1993;75(1):54‐57.841987610.1016/0030-4220(93)90406-t65

Peñarrocha
M
, 
Larrazábal
C
, 
Balaguer
J
, et al. Restoration with implants in patients with recessive dystrophic epidermolysis bullosa and patient satisfaction with the implant‐supported superstructure. Int J Oral Maxillofac Implants. 2007;22(4):651‐655.1792952866

Krämer
SM
. Oral care and dental management for patients with epidermolysis bullosa. Dermatol Clin. 2010;28(2):303‐309.2044749510.1016/j.det.2010.02.02167

Schaffer
SR
. Head and neck manifestations of epidermolysis bullosa. Clin Pediatr (Phila). 1992;31(2):81‐88.154428010.1177/00099228920310020468

Wiebe
CB
, 
Silver
JG
, 
Larjava
HS
. Early‐onset periodontitis associated with Weary‐Kindler syndrome: a case report. J Periodontol. 1996;67(10):1004‐1010.891084010.1902/jop.1996.67.10.100469

Wiebe
CB
, 
Penagos
H
, 
Luong
N
, et al. Clinical and microbiologic study of periodontitis associated with Kindler syndrome. J Periodontol. 2003;74(1):25‐31.1259359210.1902/jop.2003.74.1.2570

Yildirim
TT
, 
Kaya
FA
, 
Taskesen
M
, et al. Aggressive periodontitis associated with Kindler syndrome in a large Kindler syndrome pedigree. Turk J Pediatr. 2017;59(1):56‐61.2916836410.24953/turkjped.2017.01.00971

Kaslick
RS
, 
Brustein
HC
. Epidermolysis bullosa. Review of the literature and report of a case. Oral Surg Oral Med Oral Pathol. 1961;14:1315‐1330.1445415910.1016/0030-4220(61)90263-872

Wright
JT
. Epidermolysis bullosa: dental and anesthetic management of two cases. Oral Surg Oral Med Oral Pathol. 1984;57(2):155‐157.658362210.1016/0030-4220(84)90204-473

Wright
JT
. Comprehensive dental care and general anesthetic management of hereditary epidermolysis bullosa. A review of fourteen cases. Oral Surg Oral Med Oral Pathol. 1990;70(5):573‐578.214657910.1016/0030-4220(90)90401-d74
The Hall Technique A minimal intervention, child centred approach to managing the carious primary molar A Users Manual [Internet
]. Retrieved 2020 May 9, from https://dentistry.dundee.ac.uk/files/3M_93CHallTechGuide2191110.pdf.75

Lee
H
, 
Al Mardini
M
, 
Ercoli
C
, 
Smith
MN
. Oral rehabilitation of a completely edentulous epidermolysis bullosa patient with an implant‐supported prosthesis: a clinical report. J Prosthet Dent. 2007;97(2):65‐69.1734137210.1016/j.prosdent.2006.12.01076

Müller
F
, 
Bergendal
B
, 
Wahlmann
U
, 
Wagner
W
. Implant‐supported fixed dental prostheses in an edentulous patient with dystrophic epidermolysis bullosa. Int J Prosthodont. 2010;23(1):42‐48.2023489177

Barna
BK
, 
Eördegh
G
, 
Iván
G
, et al. [Epidermolysis bullosa: oral manifestations and their treatments]. Orv Hetil. 2017;158(40):1577‐1583.2896726710.1556/650.2017.3084478

Winter
GB
, 
Brook
AH
. Enamel hypoplasia and anomalies of the enamel. Dent Clin North Am. 1975;19(1):3‐24.16289179

Lindemeyer
R
, 
Wadenya
R
, 
Maxwell
L
. Dental and anaesthetic management of children with dystrophic epidermolysis bullosa. Int J Paediatr Dent. 2009;19(2):127‐134.1925039510.1111/j.1365-263X.2008.00940.x80

Brooks
JK
, 
Bare
LC
, 
Davidson
J
, 
Taylor
LS
, 
Wright
JT
. Junctional epidermolysis bullosa associated with hypoplastic enamel and pervasive failure of tooth eruption: oral rehabilitation with use of an overdenture. Oral Surg Oral Med Oral Pathol Oral Radiol Endodontol. 2008;105(4):e24‐e28.10.1016/j.tripleo.2007.12.0381832956481

Levy
BP
, 
Reeve
CM
, 
Kierland
RR
. The oral aspects of epidermolysis bullosa dystrophics: a case report. J Periodontol. 1969;40(7):431‐434.525724510.1902/jop.1969.40.7.43182

Haas
CD
. Epidermolysis bullosa dystrophica. Report of a case. Oral Surg Oral Med Oral Pathol. 1968;26(3):291‐295.524390510.1016/0030-4220(68)90397-683

Lin
AN
, 
Smith
LT
, 
Fine
J‐D
. Dystrophic epidermolysis bullosa inversa: report of two cases with further correlation between electron microscopic and immunofluorescence studies. J Am Acad Dermatol. 1995;33(2 II):361‐365.761588610.1016/0190-9622(95)91434-x84

Pacheco
W
, 
Marques de Sousa Araugio
R
. Orthodontic treatment of a patient with recessive dystrophic epidermolysis bullosa: a case report. Spec Care Dent. 2008;28(4):136‐139.10.1111/j.1754-4505.2008.00028.x1864737385

Wright
JT
, 
Childers
NK
, 
Evans
KL
, 
Johnson
LB
, 
Fine
JD
. Salivary function of persons with hereditary epidermolysis bullosa. Oral Surg Oral Med Oral Pathol. 1991;71(5):553‐559.204709610.1016/0030-4220(91)90361-f86

Shah
H
, 
McDonald
F
, 
Lucas
V
, 
Ashley
P
, 
Roberts
G
. A cephalometric analysis of patients with recessive dystrophic epidermolysis bullosa. Angle Orthod. 2002;72(1):55‐60.1184327510.1043/0003-3219(2002)072<0055:ACAOPW>2.0.CO;287

Peñarrocha
M
, 
Rambla
J
, 
Balaguer
J
, et al. Complete fixed prostheses over implants in patients with oral epidermolysis bullosa. J Oral Maxillofac Surg. 2007;65(7):103‐106.10.1016/j.joms.2007.03.0201758635488

Serrano‐Martínez
MC
, 
Bagán J
V
, 
Silvestre
FJ
, 
Viguer
MT
. Oral lesions in recessive dystrophic epidermolysis bullosa. Oral Dis. 2003;9(5):264‐268.1462889410.1034/j.1601-0825.2003.03971.x89

Pizzo
G
, 
Scardina
GA
, 
Messina
P
. Effects of a nonsurgical exercise program on the decreased mouth opening in patients with systemic scleroderma. Clin Oral Investig. 2003;7(3):175‐178.10.1007/s00784-003-0216-51451330590

Carroll
DL
, 
Stephan
MJ
, 
Hays
GL
. Epidermolysis bullosa–review and report of case. J Am Dent Assoc. 1983;107(5):749‐751.622764810.14219/jada.archive.1983.0322

## CHAPTER 4: Dental implants in patients with recessive dystrophic epidermolysis bullosa (RDEB)—Clinical practice guidelines

Susanne Krämer | James Lucas | David Peñarrocha Oltra | Marcelo Guzmán‐Letelier | Maria Concepción Serrano | Gustavo Molina | Sanchit Paul | Francisca Gamboa | Lorena Sepúlveda | Ignacio Araya | Carolina Arriagada | Rubén Soto | Fernanda Castrillón | Victoria Clark | Miguel Peñarrocha Diago

### Introduction

Children and adults living with recessive dystrophic EB (RDEB) present severe skin fragility. The oral mucosa is extremely friable and may slough off easily when touched. Patients present generalized blistering that may be fluid‐ or blood‐filled and arise at any oral mucosal surface, especially the tongue. Due to scarring, patients will present ankyloglossia and absence of tongue papillae and palatal rugae. The labial and buccal vestibules present severe obliteration, compromising oral hygiene procedures, dental treatment, and the wearing of removable prosthetic appliances. Progressive microstomia remains as one of the main challenges, as it gives rise to a wide variety of functional problems that include difficulties in eating, speech, and oral hygiene maintenance.^1^, ^2^, ^3^, ^4^, ^5^, ^6^, ^7^, ^8^, ^9^, ^10^, ^11^, ^12^, ^13^ Although the approach to dental treatment is focused on prevention and behavioral support, dental disease can be difficult to control, and patients may lose several teeth or even become edentulous and will need oral rehabilitation to improve their function and aesthetics.^11^

DEBRA International (DI) is the worldwide network of national groups working on behalf of those living with EB. As part of their vision to work toward ensuring access to the best quality support and medical care for people living with EB, DI entrusts the development of clinical practice guidelines (CPGs) to health care professionals with significant experience in EB. The first CPG on oral care was published in 2012.^11^ Since then, new scientific literature has become available and it has become necessary to update the guideline. As dental implants have become an increasing area of interest, with multiple high‐quality reviews in the recent years,^14^, ^15^, ^16^, ^17^, ^18^ the guideline development panel has considered it appropriate to add a new chapter on dental implants in RDEB. The present CPG on dental implants for patients with RDEB has been developed by an international panel, using a standard methodology based on a systematic review of the currently available scientific evidence and consensus meeting.

### Aim

To provide the users with information on the current best practices on dental implant‐based oral rehabilitation for patients with RDEB.

### Users

Specialists in Implantology, Oral Surgery, Rehabilitation, Oral and Maxillofacial Surgery, Special Care Dentistry and Periodontology.

The development panel recognizes that this CPG will provide information for dentists and specialists in the field of oral rehabilitation on the special approach needed for patients with RDEB. This is not a guideline for dentists to learn how to place dental implants. It can be used by patients and other health care providers to be aware of the challenges and precautions of implants in RDEB and to make evidence‐based decisions, but it will not enable them to perform the procedures.

### Target group

These guidelines can be applied to all patients diagnosed with RDEB.

### Methodology

**Question definition**

A meeting was held to define the questions to be addressed in the guideline. The selected questions are:

#### 4.1 General information

1. What is the success rate of placing dental implants in patients with RDEB?
2. What are the benefits and advantages in quality of life for patients with RDEB after rehabilitation with dental implants?
3. Which are the benefits of a fixed implant‐supported denture compared to a removable implant retained denture in patients with RDEB?
4. Which are the main challenges of placing implants in patients with RDEB?
5. Does osteoporosis affect dental implant osseointegration in patients with RDEB?
6. Which preoperative tests are needed when planning dental implants in patients with RDEB?
7. What is the best time frame between dental extractions and implant placement in patients with RDEB?
8. What are the special precautions that need to be taken when handling fragile skin and mucosa during oral surgery in patients with RDEB?
9. Would implants reduce the functional space between upper and lower arch thus limiting the space to eat?

#### 4.2 Prescriptions

10. What antibiotic prescription should be considered when planning a dental implant surgery in patients with RDEB?
11. What analgesia, anxiety, and pain management strategies should be considered when planning a dental implant surgery in patients with RDEB?

#### 4.3 Implant surgery

12. Which special considerations need to be taken during the incision when performing a dental implant surgery in patients with RDEB?
13. Which types of implants have been used successfully in patients with RDEB?
14. Which surgical technique should be used for placing dental implants in the upper maxilla of patients with RDEB?
15. Which surgical technique should be used for placing dental implants in the mandible of patients with RDEB?
16. Which type of bone grafting should be used during implant surgery in patients with RDEB?
17. Which type of interim denture has been used successfully in patients with RDEB?
18. Should implants be left to heal submerged or exposed?
19. Which type of suture can be safely used in patients with RDEB?
20. What special postoperative instructions need to be considered after implant surgery in patients with RDEB?

#### 4.4 Implant rehabilitation

21. What is the best time for loading dental implants in patients with RDEB?
22. Which types of prostheses have been used successfully in patients with RDEB?

#### 4.5 Follow‐up

23. Which complications have been reported during or after implant supported rehabilitation in patients with RDEB?
24. What is the best frequency of maintenance recall appointments after implant supported rehabilitation in patients with RDEB?

##### Systematic literature searching

###### Literature sources

The literature search ranged from 1970 to August 2018. Consulted sources included the electronic databases MEDLINE (1970 to August 2018), EMBASE (1980 to August 2018), The Cochrane Library (2018), DARE (2018), and the Cochrane controlled trials register (CENTRAL) (2018). In addition, hand‐searching journals, reviewing conference proceedings and other guidelines sources such as The US National Guideline Clearinghouse and The German Guidelines Clearinghouse were carried out.

Dissertations, conference proceedings, technical reports, and other unpublished documents that meet the selection criteria were also included. The reference lists of all papers for relevant citations were reviewed. When all the relevant studies were identified, they were sent to the experts to review for completeness.

##### Selection criteria of the articles

Primary or secondary articles in which the main topics are dental implants in patients with RDEB, published between 1970 and 2018 in any language, including case reports and case series. It was desirable for the reports to have the EB diagnoses confirmed by IFM or genetic testing; however, this was largely unavailable and could not be used as a selection criterion. The criteria used to reject articles at first‐stage screening (based on title and abstract) and second‐stage screening (based on a review of the full text) were: (a) The article does not relate to inherited EB. (b) The article describes dental treatment in patients with inherited EB, but does not consider dental implants. (c) Cohort already published in previous articles. (d) Literature review which provides no new clinical information.

##### Search strategy

To identify studies for this review, detailed search strategies were developed for each database. These were based on the search strategy developed for MEDLINE but revised appropriately for each database.

The search strategy used a combination of controlled vocabulary and free text terms based on:

#1 (Epidermolysis Bullosa):ti, ab, kw
#2 (Acantholysis Bullosa):ti, ab, kw
#3 MeSH descriptor Epidermolysis Bullosa explode all trees
#4 MeSH descriptor Dental Implants explode all trees
#5 MeSH descriptor Dental Implants, Single‐Tooth explode all trees
#6 MeSH descriptor Immediate Dental Implant Loading explode all trees
#7 (dental):ti, ab, kw
#8 (tooth):ti, ab, kw
#9 (teeth):ti, ab, kw
#10 (implant):ti, ab, kw
#11 (Osseointegration):ti, ab, kw
#12 (Implantation):ti, ab, kw
#13 #1 OR (#2 OR (#3))
#14 #4 OR(#5 OR(#6))
#15 #7 OR(#8 OR(#9))
#16 #10 OR(#11 OR(#12))
#17 #15 AND #16
#18 #14 OR #17
#19 #13 AND #18

##### Method used for formulating the recommendation

To formulate the recommendations, from the selected studies, the SIGN Guidelines were used.

**FIGURE 4.1:**
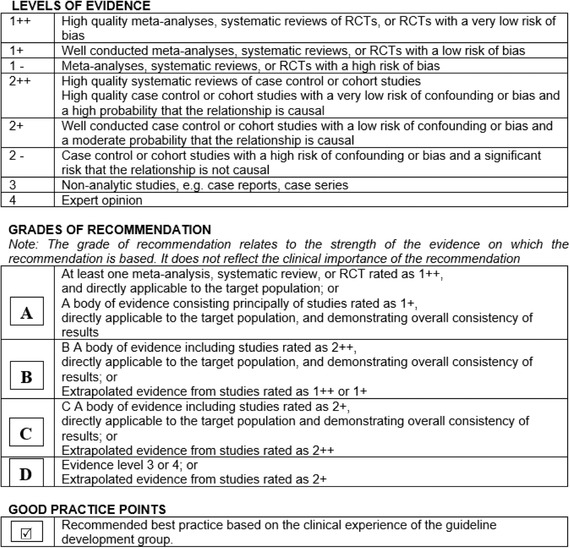
Method used for formulating the recommendations as described on the 50 Guideline Developer's Handbook, NHS Scottish Intercollegiate Guidelines Network SIGN. Revised edition January 2008^19^

##### Clinical experts and patient representatives

The information from the selected studies was gathered in evidence tables. These were analyzed and discussed by a panel consisting of clinical leads, clinical experts, patient representatives, and methodologists as described in Tables 1‐4 (see pages 2‐4). The panel meeting was held in Dubai on August 30th, 2018. In April 2019, a new literature search was performed to identify any new evidence available. The panel report was reviewed by three external specialists and a member of the multidisciplinary EB team, as well as five patient groups (Tables 3 and 5, pages 3 and 4). The final version was piloted in two centers in different countries for three months (September 2019 to December 2019) as described in Table 6, page 4. Patients and their representatives reviewed the document to make sure that the degree to which the evidence addresses patients' concerns is reflected in the guideline.

##### Guideline implementation and monitoring

**Implementation barriers**

According to the context of implementation of this guideline, some barriers to be considered are:

▪ Lack of knowledge and training of some health professionals to implement the recommendations.
▪ Lack of patient's adherence to the recommendations.
▪ Insufficient provision of health services in some parts of the world.

##### Guideline monitoring and/or auditing criteria

The implementation of these recommendations could be monitored and evaluated through audits and completing of the “CPG Evaluation Form: Pre implementation” (available at: https://surveyhero.com/c/aabc0100). The panel recommends clinical sites to conduct prepractice audit, implement the CPG, and reaudit to test improvements. Audit tools can be used from SIGN35. DI would value your feedback on the site findings to continue to improve CPG quality.

###### Further areas of research

- To evaluate the impact of dental implant rehabilitation on oral functioning.

##### Guideline updating procedure

The guideline will be updated every 5 years after its first version. If new relevant evidence is detected before the update, the information will be published on the web site http://www.debra-international.org/.

The team in charge of this update will be formed by Dr. Susanne Krämer and Dr. David Peñarrocha in 2025.

### Results

**Literature search**

The search strategy identified 23 articles. Eight were original articles on dental implants in EB, three were literature reviews, nine articles were repeated on different databases, and three articles were about EB but did not consider dental implants. Cross‐referencing allowed identifying other six additional articles on dental implants in EB. Only original articles on implants in EB were included in the tables of evidence. Review articles, repeated reports, and articles on other topics were excluded.

### 4.1 General information on implants in RDEB (questions 1 to 9)

   

**1**. **What is the success rate of placing dental implants in patients with RDEB?**

*Summary*

**TABLE 4.1:** Summary of success rate of dental implants in patients with RDEB

Appraised literature	**N° of Patients**	**N° of implants**	**1‐year osseointegration success rate**	**3‐year osseointegration success rate**	**5‐year osseointegration success rate**	**Rehabilitation success rate**
Peñarrocha ‐Diago, 2000^5^	4	15	100%	100% (7)	NA	100%
Peñarrocha, Rambla; 2007^20^	3	27	96.3% (27)	100% (18)	100% (20)	100%
Peñarrocha, 2007^21^	6	38	97.4%	100% (29)	100% (20)	100%
Lee, 2007^22^	1	8	100%	NA	NA	75%, fixed successfully
Larrazabal, 2009^8^	1	2	100%	NA	NA	100%
Oliveira, 2010^23^	1	2	100%	NA	NA	100%
Müller, 2010^24^	1	10	100%	100%	NA	50%, fixed successfully
Peñarrocha, 2011^25^	6	36	100% (36)	100% (16)	NA	100%
Peñarrocha, 2012^26^	4	23	100% (23)	100% (4)	NA	100%
Agustín‐Panadero, 2015^27^	1	8	87.5%	NA	NA	100%
Guzmán, 2016^28^	1	11	100% (6)	100% (6)	NA	100%
Agustín‐Panadero, 2017^29^	1	4	100%	NA	NA	100%
Molina, 2017^30^	1	7	100%	100%	NA	100%
Agustín‐Panadero, 2019^31^	4	31	100%	100%	NA	100%

Key: NA, information not available; *N*, total number reported in the article. Number in brackets: number of implants reported with the specific follow‐up time.

The literature available up to April 2019 reports implant‐based oral rehabilitation of 35 patients with RDEB, with a total of 222 dental implants. The osseointegration success rate after 1 year follow‐up (based on 217 implants) was 98.6%. Out of this group, 110 implants were followed for 3 years, with a success rate of 100%. The 5‐year success rate based on 40 implants is also 100%. The average oral rehabilitation success rate was 98%. The two patients who experienced fracture of their dental prosthesis had them successfully repaired.

*Level of evidence*

- Nonanalytic studies (3)

*Recommendation*


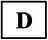
 Dental Implants can be placed successfully and restored in patients with RDEB.

**2**. **What are the benefits and advantages in quality of life for patients with RDEB after rehabilitation with dental implants?**

*Summary of evidence*

**IMAGE 4.1:**
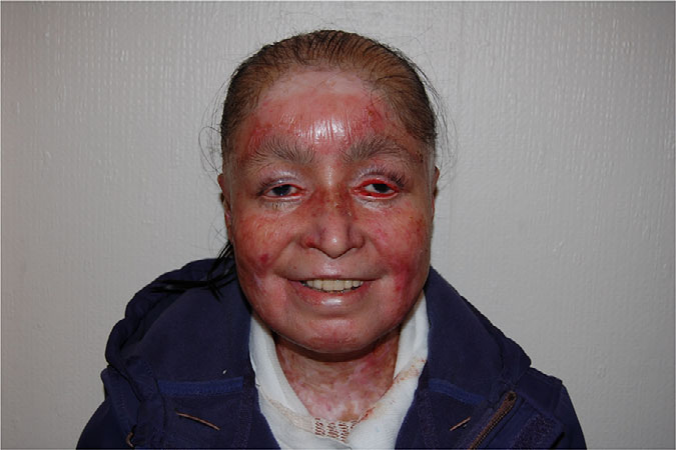
Thirty‐seven‐year‐old woman with severe recessive DEB who acknowledges improved aesthetics after complete dental implant supported rehabilitation

Oral rehabilitation with dental implants improved the patient's quality of life by:

1. Oral function: ^5^, ^8^, ^20^, ^21^, ^22^, ^23^, ^24^, ^25^, ^26^, ^27^, ^28^
  - Improved chewing (helping to improve nutrition)
  - Improved oral stage of swallowing
  - Improved speech
2. Aesthetics, increasing self‐esteem and sociability (Image 4.1).^5^, ^8^, ^20^, ^21^, ^23^, ^24^, ^25^, ^26^, ^27^, ^28^
3. Comfort, because of the improved retention of the prostheses.^21^
4. Oral health status, as it reduced the risk of soft tissues trauma.^20^, ^28^

*Level of evidence*

- Nonanalytic studies (3)

*Recommendation*


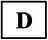
 Performing dental implants in patients with RDEB is recommended, as patients improve their quality of life through enhanced oral functioning, aesthetics, comfort, and general oral health status.^5^, ^8^, ^20^, ^21^, ^22^, ^23^, ^24^, ^25^, ^26^, ^27^, ^28^, ^29^

**3**. **Which are the benefits of a fixed implant‐supported denture compared to a removable implant‐retained denture in patients with RDEB?**

*Summary of evidence*

Fixed implant‐supported dentures produce less risk of soft tissue trauma. There is less mechanical irritation to the oral mucosa and patients do not need to remove them.^20^, ^21^, ^22^, ^24^, ^28^

Fixed implant‐supported dentures result in a higher satisfaction level as reported by the patients, when compared to removable implant‐supported dentures.^21^, ^25^

*Level of evidence*

- Nonanalytic studies (3)

*Recommendation*


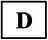
 Fixed implant supported dentures should be preferred as they provide more advantages than removable implant supported dentures.^21^, ^25^

**4**. **Which are the main challenges of placing implants in patients with RDEB? (If reported, specify the patient's maximal mouth opening.)**

*Summary of evidence*

**IMAGE 4.2:**
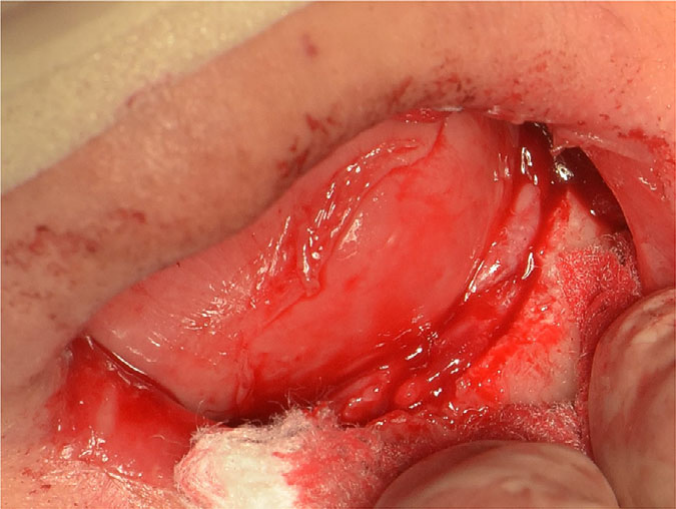
Severe microstomia limiting access to surgical field, mucosal sloughing can be observed on the tongue surface

**IMAGE 4.3:**
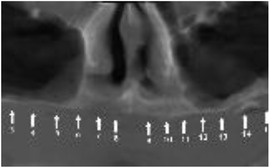
Limited alveolar height on a maxillary bone of a patient with RDEB

The main challenges when performing implant surgery in patients with RDEB are:

1. Accessing the surgical site is complicated because of severe microstomia, severe ankyloglossia, and obliteration of oral vestibule (Image 4.2).^5^, ^20^, ^21^, ^23^, ^24^, ^25^, ^26^, ^27^, ^28^, ^29^
2. Surgery is challenged by skin and mucosa fragility, tissue sloughing, and formation of blood‐filled bullae and blistering complications at minor trauma (Image 4.2).^5^, ^8^, ^20^, ^21^, ^23^, ^24^, ^25^, ^26^, ^27^, ^28^
3. Choosing a site to place the implant is challenged by severe alveolar bone atrophy (Image 4.3).^20^, ^25^, ^29^
4. Oral rehabilitation (dental prosthesis) is challenged by microstomia and difficulty in taking impression.^20^, ^23^, ^25^, ^27^, ^29^

*Level of evidence*

- Nonanalytic studies (3)

**5**. **Does osteoporosis affect dental implant osseointegration in patients with RDEB?**

*Summary of evidence*

Even though osteoporosis is a clinical feature of RDEB^32^ and clinically patients present with severe alveolar bone atrophy,^5^, ^20^, ^21^, ^25^, ^26^, ^33^ implants osseointegrate successfully. The success rate after 1 year, as observed in question 1, is 98.6%.

*Level of evidence*

- Nonanalytic studies (3)

*Recommendation*


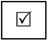
 Implant success rate does not seem to be affected by osteoporosis (osseointegration 98.6% after 1 year). Nevertheless, the panel of experts suggests conventional or delayed loading of the implants to minimize the risk of failure. More information is provided in question 21.

**6**. **Which preoperative tests are needed when planning dental implants in patients with RDEB?**

*Summary of evidence*

Reported clinical cases and case series have used panoramic radiograph,^5^, ^8^, ^20^, ^21^, ^23^, ^25^, ^26^, ^27^, ^29^, ^30^, ^31^ cone‐beam computerized tomography,^5^, ^8^, ^20^, ^21^, ^23^, ^25^, ^26^, ^27^, ^28^, ^29^, ^30^, ^31^, ^33^ and stereolithographic models^23^ to plan the surgery and rehabilitation.

**IMAGE 4.4:**
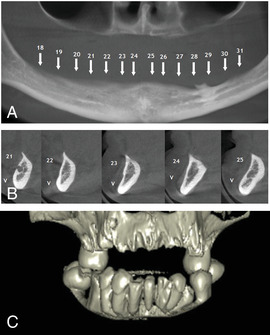
Panoramic radiograph (A) and cone‐beam tomography (B and C) are the main preoperative images needed for surgery planning

*Level of evidence*

- Nonanalytic studies (3)

*Recommendation*

Imaging:


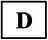
 When planning implant surgery, a panoramic radiograph and cone‐beam‐computerized tomography should be assessed (Image 4.4)

Models:


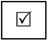
 If available, stereolithographic models can be useful to help plan the implant surgery in a noninvasive manner.

**7**. **What is the best time frame between dental extractions and implant placement in patients with RDEB?**

*Summary of evidence*

There have been reports of immediate implant placement after extraction,^23^, ^27^, ^29^ as well as implant surgery 3 months^28^ and 6 months after extraction.^22^ The literature reviewed does not provide enough information on the diagnosis of each tooth, in order to provide a clear guideline or recommendation.

*Level of evidence*

- Nonanalytic studies (3)

*Recommendation*


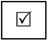
 Whenever possible, immediate implant placement after the dental extraction should be considered to reduce the number of interventions.

**8**. **What are the special precautions that need to be taken when handling fragile skin and mucosa during oral surgery in patients with RDEB?**

*Summary of evidence and recommendations*

**IMAGE 4.5:**
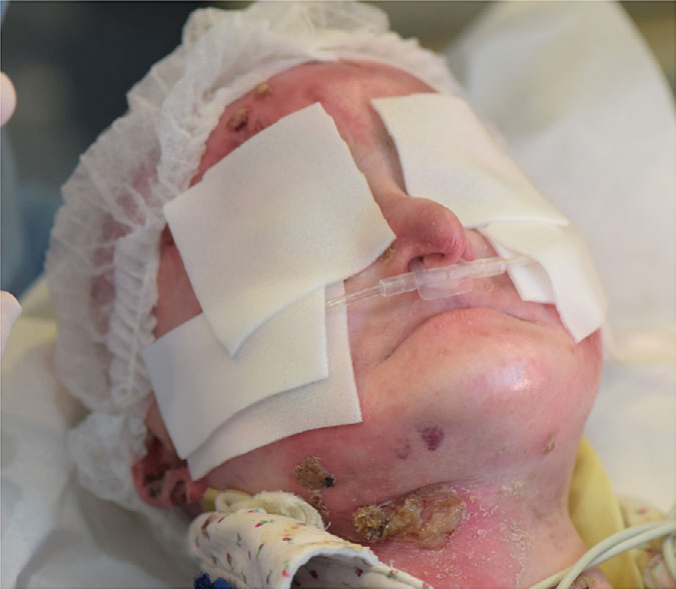
Nonadherent dressings protecting the skin and eyes. Lips are well lubricated

**IMAGE 4.6:**
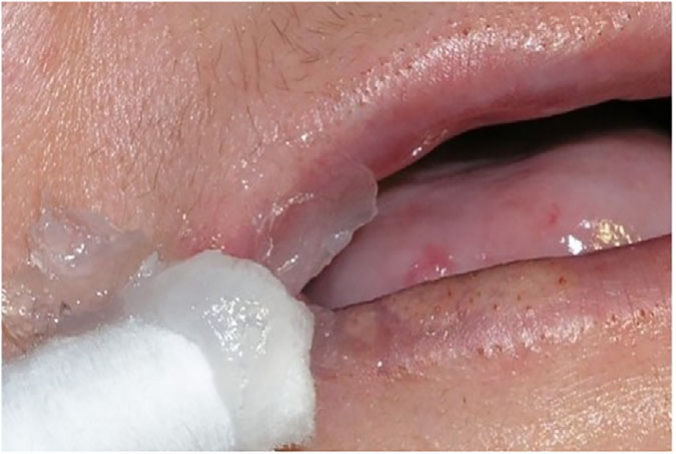
Lips lubricated with petrolatum

**IMAGE 4.7:**
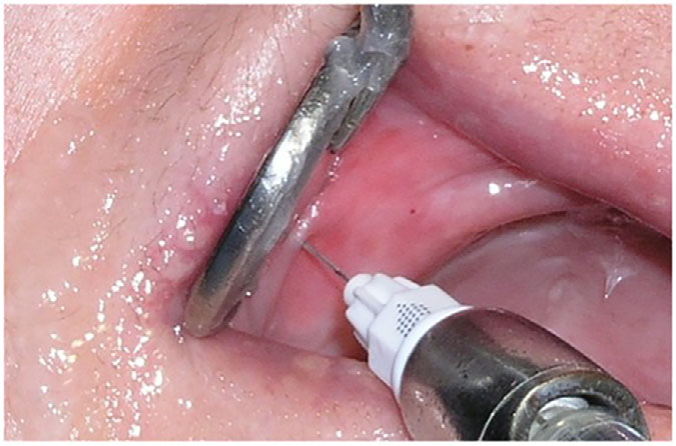
Local infiltration should not be superficial, as it could separate the mucosal layers

**IMAGE 4.8:**
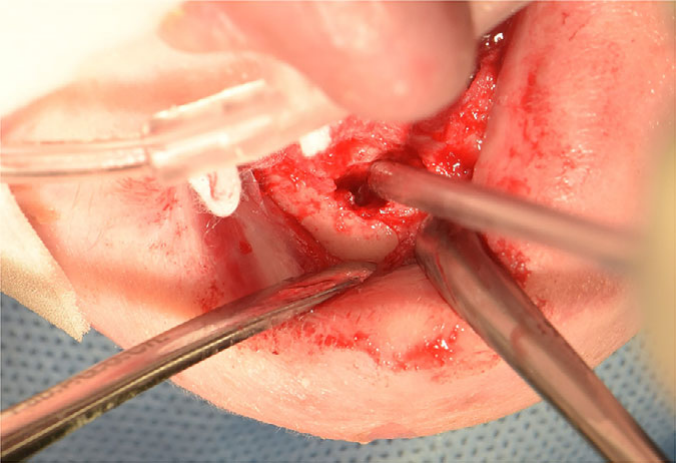
Aspirator or suction tip should be leaned on hard tissue (bone)

**IMAGE 4.9:**
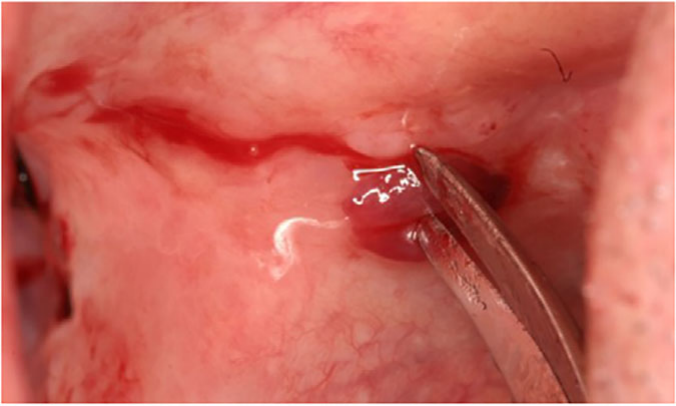
Blood‐filled bulla drained with a sterile scissor

- Patient's skin should be carefully cleaned with antiseptics prior to surgery and cover with lubricants or emollients to reduce the possibility of mechanical trauma to the skin. Special nonadherent dressings can be used (Image 4.5).^20^, ^21^, ^23^ More information can be found in Chapter 3: Oral health care and dental treatment for children and adults living with EB—CPGs.
- Extreme care when handling skin and mucosa will avoid or reduce the formation of new lesions. To handle tissues, a little pressure (compressive forces) can be applied, but no sliding movements (lateral traction or other shear forces) should be used, as these can cause tissue sloughing. Be gentle when stretching mucosa with intraoral mirror. Avoid friction between intraoral mirror or separators and mucosa.^5^, ^25^, ^29^, ^33^
- Lips should always be lubricated before any procedure is performed; to reduce adherence, shearing forces leading to tissues separation, and lesions formation (Image 4.6).^5^, ^8^, ^20^, ^25^, ^26^, ^27^, ^28^, ^29^, ^31^, ^33^ Examples of commercially available lubricants or emollients are petrolatum Vaseline^®^ (Unilever) and Linovera^®^ (B.Braun).
- When using local anesthesia, the liquid should be injected deeply and slowly into the tissues to prevent tissue distortion that may cause tissue separation and blistering (Image 4.7).^5^, ^21^, ^23^, ^26^, ^28^
- To reduce the need of aspiration, a minimal amount of irrigation with saline should be used. Avoid contact of aspirator with mucosa. The aspirator should be in contact with bone and not the soft tissues to avoid tissue sloughing (Image 4.8).^5^, ^8^, ^25^, ^26^, ^28^, ^33^
- Use small suction tips and flat tissue retractors.^23^
- Blood or fluid‐filled bullae that occur during treatment should be drained with a sterile needle or by a cut with scissors to avoid lesion expansion due to fluid pressure (Image 4.9).^29^

**9**. **Would implants reduce the functional space between upper and lower arch thus limiting the space to eat?**

*Summary of evidence*

The changes in functional space between upper and lower arch before and after implant rehabilitation, which is a concern for patients considering this treatment, have not been specifically quantified in the literature. However, data published on mouth opening and quality of life can be an aid for patients undergoing an evidence‐based decision process.

*Quantitative measurement and changes*:

The patient receiving full arch fixed prothesis by Molina in 2017 had an initial maximal mouth opening of 22 mm and her quality of life improved after rehabilitation.^30^ Lee, in 2007, highlighted the importance of a framework wax‐up to assess the functional space, and analyses that fixed complete dentures require less space than overdentures, therefore allowing more functional space.^22^

*Qualitative changes*:

Patients unable to bite before treatment reported to be able to chew better after the rehabilitation.^20^ The patients’ masticatory function was self‐reported as having improved considerably as a result of treatment.^8^, ^27^

The general satisfaction questionnaire after oral implant rehabilitation (considering general satisfaction, eating, speech, aesthetics, hygiene, comfort, and self‐esteem on a scale from 0 to 10, being 0 = “not at all satisfied” and 10 = “totally satisfied”) scored on average: 9.6,^21^ 9.0,^25^ 9.2,^26^ and 9.5.^28^ Specifically for the question on satisfaction with “Eating ability” after fixed rehabilitation in the same scale, 25% of the patients scored 9 and 75% of the patients scored 10.^31^

*Level of evidence*

- Nonanalytic studies (3)

*Recommendation*


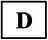
 In patients with partial edentulism, fixed implant‐supported oral rehabilitation improves the eating ability.


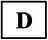
 In patients with total edentulism, a fixed full‐arch oral rehabilitation allows the mastication.

### 4.2 Prescriptions (questions 10 and 11)

   

**10**. **What antibiotic prescription should be considered when planning a dental implant surgery in patients with RDEB?**

*Summary of evidence*

Most authors prescribe amoxicillin 500 mg every 8 hours for 7 days.^5^, ^8^, ^20^, ^21^, ^23^, ^25^, ^26^, ^27^, ^29^, ^31^ One author also includes a preoperative dose^23^ and another author added clavulanic acid to the amoxicillin.^28^ There is no report of any infectious related complication. None of the patients had known antibiotic allergy. History of allergies always needs to be assessed on an individual basis and considered prior to prescription.

*Level of evidence*

- Nonanalytic studies (3)

*Recommendation*


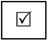
 Antibiotic treatment should follow standard implant surgery protocols.

**11**. **What analgesia, anxiety, and pain management strategies should be considered when planning a dental implant surgery in patients with RDEB? (Consider preoperative, perioperative, and postoperative measures.)**

*Summary of evidence*

Analgesia and pain management

Only one author used preoperative pain management, more precisely piroxicam 20 mg 1 hour before surgery.^23^ During surgery, most authors used articaine 4% as the local anesthesia of choice.^8^, ^25^, ^26^, ^27^, ^28^, ^29^ Other authors have used mepivacaine 2%.^23^

To manage postoperative pain, most authors prescribed ibuprofen 600 mg every 8 hours for 3 to 7 days,^8^, ^20^, ^21^, ^25^, ^26^, ^27^, ^28^, ^29^, ^31^ while others prescribed ibuprofen 400 mg every 8 hours for 3 days^5^ or piroxicam 20 mg once a day for 5 days.^23^

Anxiety management

While some authors use only behavioral management techniques,^23^, ^29^ most authors use sedation^8^, ^20^, ^21^, ^25^, ^26^, ^27^, ^28^, ^31^ and others general anaesthesia.^22^, ^24^

*Level of evidence*

- Nonanalytic studies (3)

*Recommendation*

For the pain management:

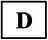

  Use of local anesthesia, injected deeply into the tissue at a sufficiently slow rate to prevent tissue distortion.
2. The use of NSAID postoperative, following standard protocols.

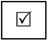
 For anxiety management during surgery, we suggest revising the section on behavioral support (Chapter 3) and Chapter 5 on sedation and anesthesia.

### 4.3 Implant surgery (questions 12 to 20)

   

**12**. **Which special considerations need to be taken during the incision when performing a dental implant surgery in patients with RDEB?**

*Summary of evidence*

**IMAGE 4.10:**
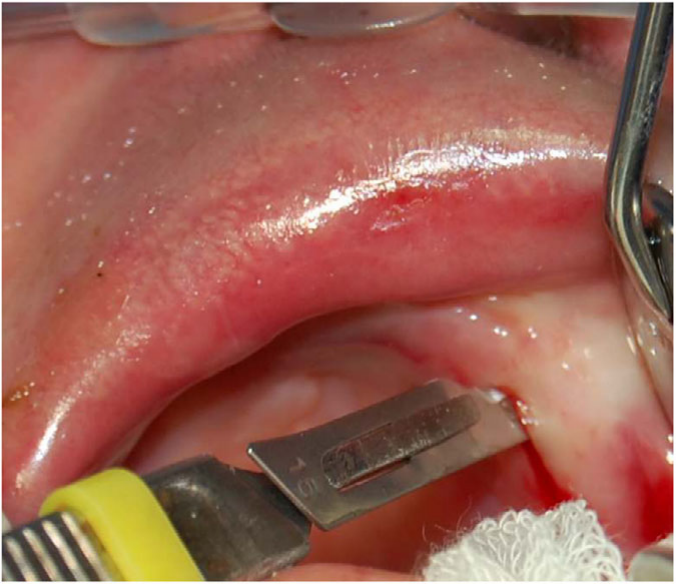
Supracrestal incision

**IMAGE 4.11:**
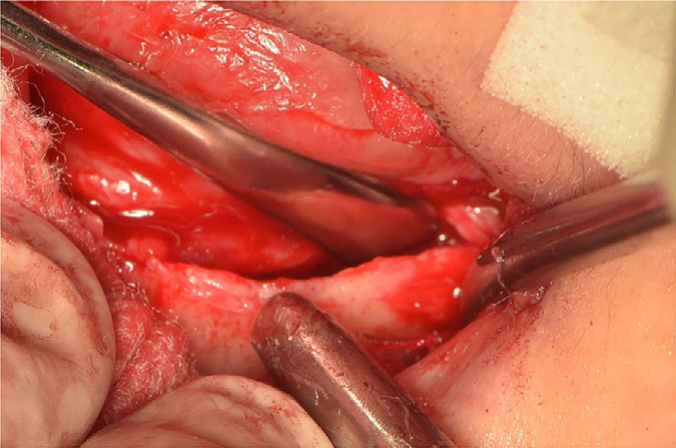
Mucoperiostal detachment

Most authors perform extensive supracrestal incisions with small lateral discharges to allow the separation of sufficiently large full‐thickness flaps with the aim of preventing tension to the flap during retraction, as the force applied by separators could cause mucosal damage (Image 4.10).^5^, ^8^, ^20^, ^27^, ^28^, ^33^ The tissues must be tractioned in a gently way.^8^, ^23^, ^33^ The detachment must be mucoperiosteal, complete, and careful (Image 4.11).^8^, ^27^, ^33^

Only one author has described a different technique. Molina, in 2017, performed a flapless surgical approach for the installation of mini‐implants.^30^

*Level of evidence*

- Nonanalytic studies (3)

*Recommendation*

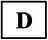
The supracrestal incision should be extensive and include release incisions to expose the bone.
- The detachment must be mucoperiosteal (full thickness), complete, and careful.
- The tissues must be tractioned in a gently way.

**13**. **Which types of implants have been used successfully in patients with RDEB?**

*Summary of evidence*

As observed on the table of evidence (Table 4.2), all the publications of implants in EB report the use of endosseous implants. Most authors have used tissue level implants, while Oliveira in 2010 chose bone‐level implants. Self‐tapping implants have been placed, either tapered or straight. Bioactive and modified surface implants have been used. One author used mini implants.

**TABLE 4.2:** Types of dental implants used in previous publications

**Reference**	**Type of Implant**	**Brand name**
Peñarrocha‐Diago, 2000^5^	Endosseous—Tissue level Titanium Plasma Sprayed (TPS)	ITI (Straumann)
Peñarrocha, Ramblas, 2007^20^		
Peñarrocha, 2007^21^		
Lee, 2007^22^	Endosseous—Tissue level titanium sandblasted/acid‐etched (SLA) surface	Straumann standard plus
Larrazabal, 2009^8^	Endosseous—Tissue level Titanium Avanblast Surface—Self‐tapping	Defcone TSA
Oliveira, 2010^23^	Endosseous—Bone level TPS + Hydroxyapatite (HA)	IMZ twin implants
Peñarrocha, 2011^25^	Endosseous—Tissue level Chemically modified / SLA surface	Phibo TSA Avanblast surface/ITI SLA surface
Peñarrocha, 2012^26^		
Agustín‐Panadero, 2015^27^	Endosseou—Tissue level chemically modified	Phibo TSA Avanblast surface
Guzman, 2016^28^	Endosseous—Tissue level—self‐tapping Titanium—TMX surface (physical surface features) HA MP1 covering (chemically modified surface)	Zimmer TSV
Agustín‐Panadero, 2017^29^	Endosseous—Tissue level Titanium grade II—chemically modified	Phibo TSA Avanblast surface
Molina, 2017^30^	Endosseous—Tissue level Self‐tapping‐ chemically modified—Mini‐implants	Not reported
Agustín‐Panadero, 2019^31^	Endosseous—Tissue level Titanium grade II—chemically modified	Phibo TSA Avanblast surface

*Level of evidence*

- Nonanalytic studies (3)

*Recommendation*

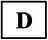
Endosseous self‐tapping implants have been used successfully in patients with RDEB.
- The shape can be either tapered or straight and the surface can be bioactive or modified, depending on the patient's bone quality.

**14**. **Which surgical technique should be used for placing dental implants in the upper maxilla of patients with RDEB?**

*Summary of evidence*

**IMAGE 4.12:**
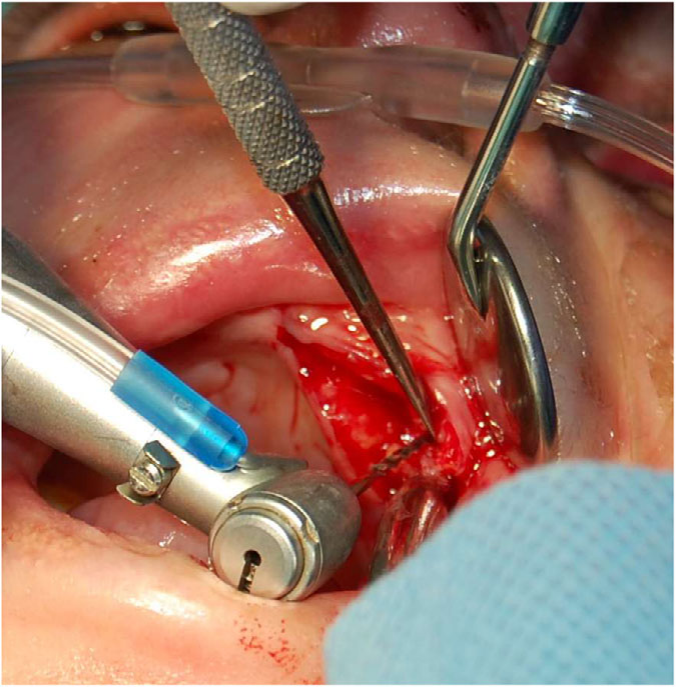
Low‐velocity drilling

It is widely described that patients with RDEB present marked alveolar atrophy. Therefore, most authors agree that conventional mechanized instruments could weaken the residual bony process, thus precluding primary retention of the implants. In 2000, Peñarrocha^5^, ^33^ described the successful use of the osteotome technique in the maxilla of patients with RDEB. Thereafter this technique has widely been used by most authors. Depending on the clinical features of the patients, drills are combined with osteotomes to preserve the bone and facilitate achievement of primary stability (Images 4.12 to 4.14).^20^, ^21^, ^25^, ^26^, ^28^, ^31^ Irrigation with saline is used as minimal as possible to avoid damage caused by the action of the suction tip.^24^, ^27^, ^29^, ^31^ The only author describing a different technique was Müller who published a clinical case using the underdrilling technique.^24^

**IMAGE 4.13:**
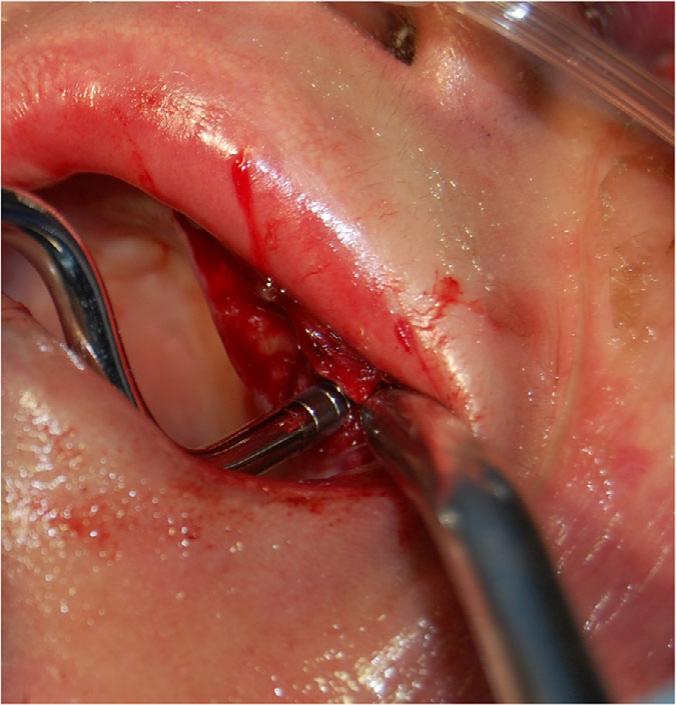
Expansion osteotomes

**IMAGE 4.14:**
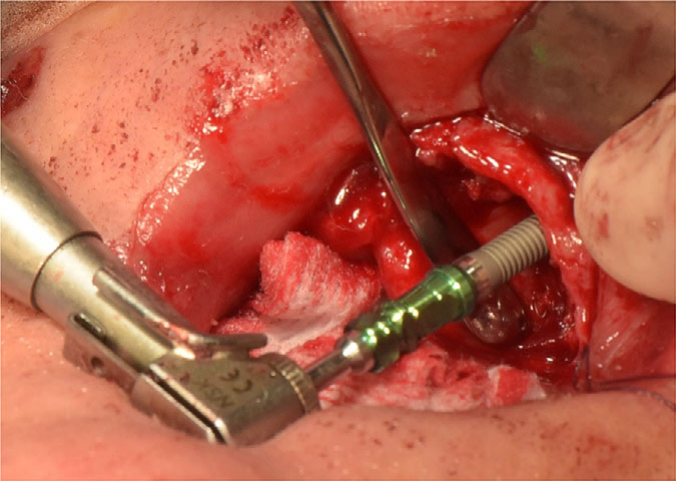
Implant insertion

*Level of evidence*

- Nonanalytic studies (3)

*Protocol recommended by the panel of experts for placing dental implants in the upper maxilla of patients with RDEB*

∘ For patients with severe atrophy of the maxillary bone, a combined drill and osteotome technique is recommended: ^5^, ^20^, ^21^, ^25^, ^26^, ^33^ 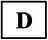

  ▪ An initial low‐velocity drill with minimal irrigation is used to create a small opening in the residual bone to allow for the insertion of osteotomes of small diameter.^28^
  ▪ Expansion osteotomes are used to facilitate obtaining primary stability while avoiding bone removal.
  ▪ The implants are inserted using a low‐velocity drill technique with no irrigation.^27^, ^29^

**15**. **Which surgical technique should be used for placing dental implants in the mandible of patients with RDEB?**

*Summary of evidence*

**IMAGE 4.15:**
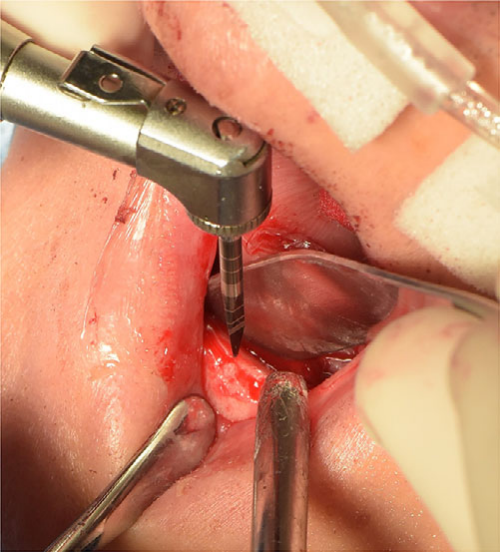
Rotatory technique with little saline irrigation. Suction tip leaned on the bone

In the mandible, the conventional rotatory technique is used to place dental implants in patients with RDEB (Image 4.15).^5^, ^8^, ^20^, ^21^, ^25^, ^26^, ^31^, ^33^ The amount of irrigation with saline varies between authors.

Specific precautions should be taken, such as:

∘ Securing a sufficiently large surgical field to allow working on the bone without inducing soft tissue tension.^20^, ^21^
∘ Minimum sterile saline solution should be used.^20^, ^31^ There is a report where abundant irrigation with saline was used, but the surgical management was complicated due to the formation of bleeding bullae after minor trauma.^8^ Therefore, the panel of clinical experts recommends the use of moderate amount of irrigation.
∘ Aspiration should be done with the suction tip in contact with the bone, not the soft tissues.^20^, ^21^, ^31^

Müller and coworkers used the same technique as for the maxilla: the underdrilling technique, with the aim of improving primary stability.^24^ This should be considered to enhance primary stability if the surgeon detects that the bone has low density while drilling.

Two authors have planned to use guided surgeries.^22^, ^23^ In the first report, the surgical guide was used successfully to place two single implants in the area of the lower central incisors.^23^ In the second report, however, the surgery was performed on a completely edentulous patient and the surgeons were unable to stabilize the mandibular surgical guides due to intraoral soft tissue scars, having to place the implants without a guide.^22^

It is considered a good practice to obtain a postoperative panoramic radiograph^25^, ^26^, ^31^

*Level of evidence*

- Nonanalytic studies (3)

*Protocol recommended by the panel of experts for placing dental implants in the mandible of patients with RDEB*

∘ Conventional rotatory technique with:
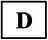

  ▪ Large surgical field to allow working on the bone without inducing blisters.
  ▪ Reduced amount of sterile saline solution.
  ▪ Suction tip in contact with the bone, not the soft tissues.
  ▪ Consider undersized drilling if atrophic bone hinders primary stability.

**16**. **Which type of bone grafting should be used during implant surgery in patients with RDEB?**

*Summary of evidence*

**IMAGE 4.16:**
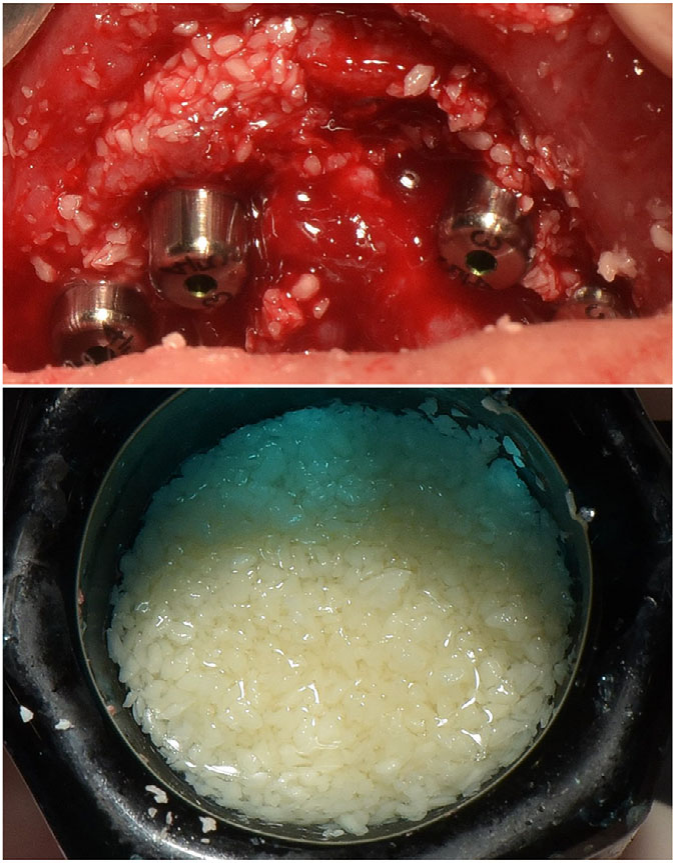
Tricalcium phosphate synthetic particulate

According to the available body of evidence, it is possible to use bone grafting to regenerate peri‐implant defects.^8^, ^25^, ^26^, ^29^ Autologous bone collected from the formation of the implant bed or from a donor site,^8^, ^25^, ^26^, ^31^ block graft from retromolar area fixed with osteosynthesis screws,^8^ tricalcium phosphate synthetic particulate bone (Image 4.16),^25^, ^26^, ^29^, ^31^ and collagen absorbable membranes^25^, ^26^, ^31^ have been used successfully. A nonabsorbable titanium‐reinforced membrane has also been reported in one case.^26^

*Level of evidence*

- Nonanalytic studies (3)

*Recommendation* 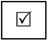

∘ Use the standard surgical bone graft protocols depending on each case.
∘ Absorbable membranes are preferable to avoid a second surgery.

**17**. **Which type of interim denture has been used successfully in patients with RDEB?**

*Summary of evidence*

Oliveira reported the use of an immediate prosthetic rehabilitation. This case reports two single tooth replacements using dental implants.^23^ Only 2 out of 13 authors describing full arch rehabilitation reported the use of an interim (temporary) removable prosthesis during the osseointegration period. The first was tissue‐supported and was lined with tissue conditioner every 2 weeks,^31^ the second was implant‐supported.^30^ Other authors considered interim dentures difficult to use.

*Level of evidence*

- Nonanalytic studies (3)

*Recommendation*

There is insufficient evidence to provide a recommendation on this question. Single‐tooth replacement might be able to use an interim restoration. For full arch rehabilitation, it will depend on the patient's mucosal tolerance to wear interim dentures.

**18**. **Should implants be left to heal submerged or exposed?**

*Summary of evidence*

**IMAGE 4.17:**
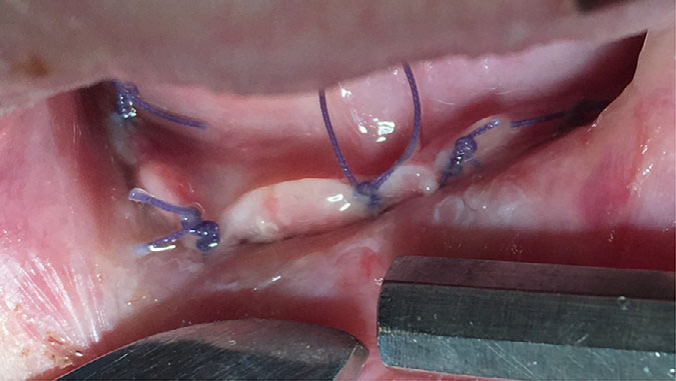
Implants left submerged after surgery. Resorbable sutures

Both techniques have been used in patients with RDEB.^28^ Implants in which bone regeneration was performed were left submerged (Image 4.17).^25^, ^31^ When implants were left submerged, second‐stage surgery was performed between 3 and 6 months later.^8^, ^27^, ^31^

**IMAGE 4.18:**
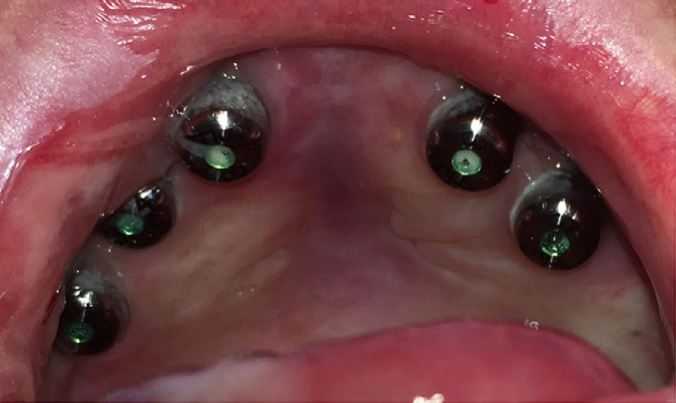
Exposed healing caps after surgery and prior to rehabilitation

**IMAGE 4.19:**
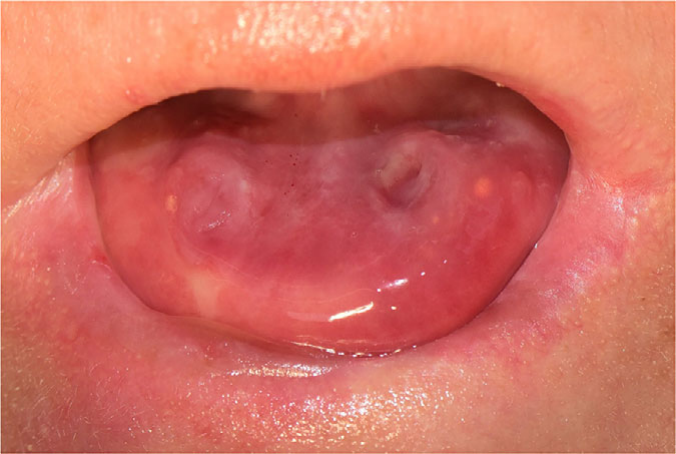
Implant caps indents on the tongue during osseointegration period

It has been observed that during the osseointegration period, the rounded healing caps can cause ulcers on the tongue (Image 4.18 and 4.19).^24^ This, however, does not contraindicate its use.

*Level of evidence*

- Nonanalytic studies (3)

*Recommendation*


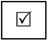
 There is no difference in decision process when compared to healthy patients. The first choice is to leave the implants exposed to heal, to avoid second surgery. Implants are left to heal submerged in patients with RDEB whenever primary stability is not achieved, when bone regeneration procedures are done simultaneously to implant placement, or if there are oral hygiene difficulties.

**19**. **Which type of suture can be safely used in patients with RDEB?**

*Summary of evidence*

Suturing techniques successfully used include horizontal mattress^8^ and sutures applied with no^5^ or little^33^ tension. The types of sutures successfully used include silk sutures (4‐0)^23^ and absorbable polyglactin sutures (3‐0) (Vicryl, Johnson & Johnson, New Brunswick, NJ, USA) (Image 4.17).^28^ In all the cases, the sutures were removed after 1 week.^23^, ^25^, ^26^, ^27^, ^29^

*Level of evidence*

- Nonanalytic studies (3)

*Recommendation* 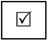

∘ Sutures have to be applied without tension.
∘ Avoid rigid sutures that will damage the soft tissues during the postoperative phase.

**20**. **What special postoperative instructions need to be considered after implant surgery in patients with RDEB?**

*Summary of evidence*

It is important to highlight that even though patients with RDEB have marked skin fragility; ulcers and tissue sloughing occurs during surgery; postoperative course is normal in most patients.^25^ The postoperative instructions given to the patients include the use of ultrasoft toothbrushes and to perform daily mouthwashes with an alcohol‐free chlorhexidine.^23^, ^30^ Patients were instructed to eat cold liquid food for 48 hours postsurgery.^23^ Mouth rinses aimed at tissue healing such as Gelclair^®^ (Helsinn Healthcare SA, Switzerland), Dentoxol^®^ (Ingalfarma, Chile), and K‐trix^®^ (Farpag, Colombia) can be considered during the postoperative course. A patient representative reported successful use of saltwater mouthwash as a low cost, easily accessible alternative. No studies have been identified on their impact for intraoral tissue healing after surgery in patients with EB.

**IMAGE 4.20:**
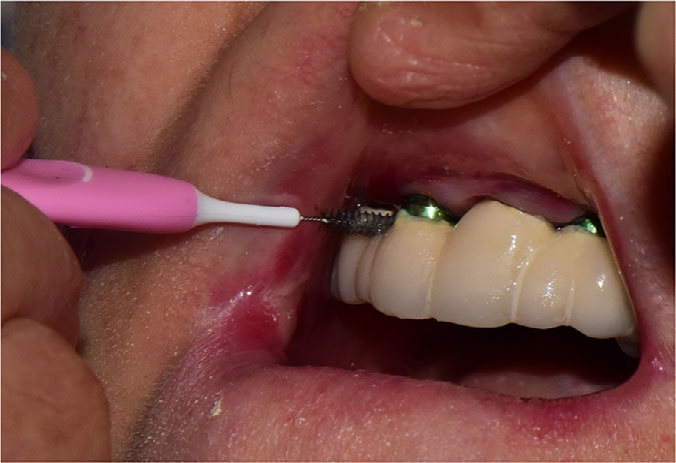
Oral hygiene cleaning the intaglio surface with interproximal brush

Long‐term instructions include brushing the prosthesis with a soft brush while avoiding the soft tissues. The hygiene of the intaglio surface of the prosthesis could be carefully addressed via a dental water flosser using a low‐pressure setting^29^ or an interproximal brush (Image 4.20).

*Level of evidence*

- Nonanalytic studies (3)

*Recommendation* 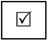

∘ Ultrasoft toothbrush to clean the wound and the healing abutments.
∘ Mouthwash: rinsing the mouth with an alcohol‐free chlorhexidine 0.12% mouthwash twice a day for 7 to 10 days, or a mouthwash aimed at tissue healing, can be considered.
∘ The patient was instructed to eat cold, liquidized food for 48 hours postsurgery.

### 4.4 Implant rehabilitation (questions 21 and 22)

   

**21**. **What is the best time for loading dental implants in patients with RDEB?**

*Summary of evidence*

Implants have been left to osseointegrate for 3 months in the mandible^5^, ^20^, ^21^, ^25^, ^26^, ^27^, ^30^ and 6 months in the maxilla.^5^, ^8^, ^20^, ^21^, ^22^, ^23^, ^25^, ^26^, ^27^

*Level of evidence*

- Nonanalytic studies (3)

*Recommendation* 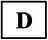

∘ Conventional or delayed loading protocols should be followed to minimize the risk of failure. Implants should be loaded after 3 months in mandible and after 6 months in maxilla.

**22**. **Which types of prostheses have been used successfully in patients with RDEB?**

*Summary of evidence*

Impression taking

The first step to design prostheses is to take an adequate impression. Due to the patient's limited mouth opening, this procedure can be challenging.^22^ Most clinicians do manage to take the impressions without the need of pharmacological patient management; however, a case of impressions and bite registration during general anesthesia has been reported.^24^

**IMAGE 4.21:**
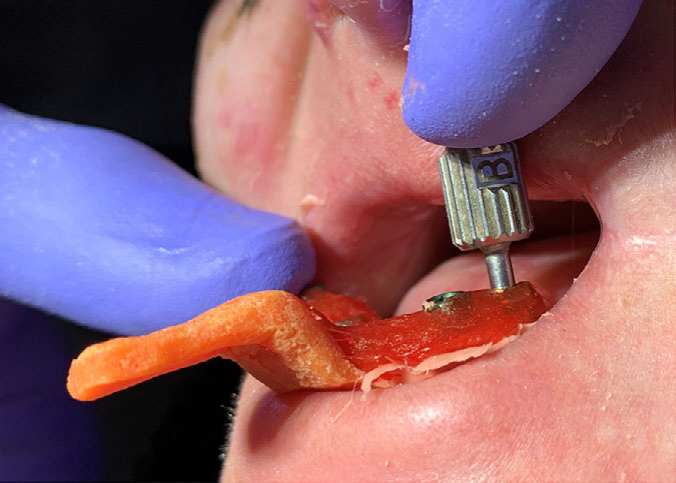
Customized acrylic tray used for an open tray technique. Copings were secured with acrylic resin. Patients’ limited mouth opening is a challenge for screw manipulation

As regard to the trays, only two authors have reported the use of conventional trays to take the impressions.^24^, ^27^ The other authors have designed small, customized, acrylic individual trays^28^, ^33^ or placed heavy silicone directly in the mouth.^29^ Both the closed^27^, ^28^ as well as the open tray techniques have been used. The copings have been secured to the implants and connected with dental floss and joined with acrylic resin (Image 4.21).^22^, ^23^ Another alternative is to connect the copings with plastic sprue bars and acrylic resin.^22^ For the maxillary impression, one author modified the surgical guide to use it as a custom tray.^53^ Medium or light‐bodied viscosity impression material is applied around the assembly to record the position of the implants.^22^, ^23^ To pour the impression, the analogs are connected to the impression copings and artificial stone together with gingival mask is used to fabricate the definitive cast.^23^, ^27^ Facebow records and a maxillofacial relation record can be obtained.^22^

Recently, Agustín‐Panadero and coworkers reported the use of digital intraoral scanners to register the implant position, avoiding the need of conventional impression technique.^31^

Oral rehabilitation

**IMAGE 4.22:**
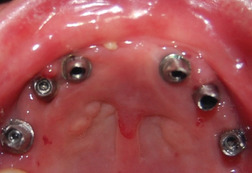
Implant abutments

The first reports of oral rehabilitation with dental implants were published by Peñarrocha in 2000, using overdentures.^5^ Thereafter the same group, as well as all the other authors, have chosen fixed (either cemented^27^, ^28^ or screwed^24^, ^29^) rehabilitation.^8^, ^23^, ^26^ Most rehabilitations consist of fixed short arch implant‐supported prosthesis for complete edentulous patients.^20^, ^22^, ^25^, ^26^, ^27^, ^28^, ^29^, ^30^, ^31^ The short arch (one premolar and one molar) is chosen because of the severity of microstomia.^20^, ^24^, ^25^, ^26^, ^27^, ^30^, ^33^ The materials used include metalceramic (Images 4.22 to 4.24),^23^, ^27^, ^28^ gold alloy framework with acrylic resin denture teeth,^24^ and metal‐resin (when financial limitations do not allow the use of metal‐ceramic).^22^ Some specific denture design modifications to overcome difficulties related to microstomia include: the superstructure had to be horizontally screwed onto a milled bar mesostructure, because the limited space precluded access with a screwdriver for direct vertical screw retention.^24^ Some authors have had screw‐access holes perforating the buccal surface of the denture and have closed them using composite resin.^22^, ^31^ This specific modification has been necessary either because the mandibular implants were placed labially^22^ or to overcome the difficulty of accessing the screws due to microstomia.^31^ As to the prostheses design, bibalanced occlusion is chosen.^33^ The design needs to allow space between the intaglio surface of the denture and the oral mucosa so that the prosthesis can be easily cleaned. Only the maxillary anterior area has slight contact on the mucosa to minimize possible phonetic problems.^22^

**IMAGE 4.23:**
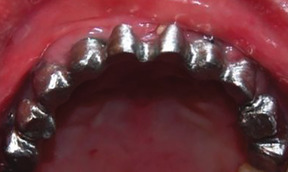
Metal framework

**IMAGE 4.24:**
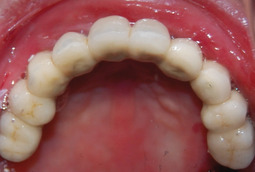
Fixed short arch metalceramic complete oral rehabilitation

Summary

Types of prostheses that have been reported as successful in patients with RDEB: (a) Fixed full‐short arch prostheses, (b) fixed overdenture, and (3) fixed partial denture.

*Level of evidence*

- Nonanalytic studies (3)

*Recommendation*

Specific patient's oral structures need to be considered to choose the type of prosthesis that fits their specific requirements. The first option should be:

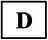
For edentulous patients: Fixed full‐short arch prostheses.
- For single implants: Fixed partial denture.

### 4.5 Follow‐up (questions 23 and 24)

   

**23**. **Which complications have been reported during or after implant supported rehabilitation in patients with RDEB?**

*Summary of evidence*

During implant surgery

**IMAGE 4.25:**
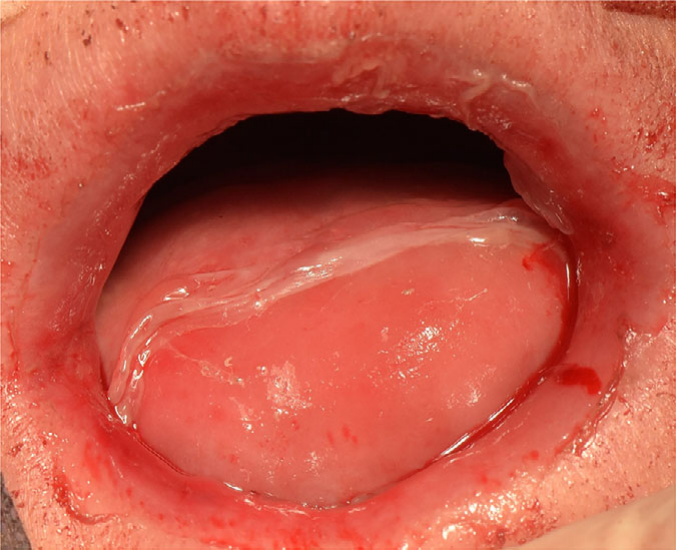
Tongue mucosa sloughing during implant surgery

In all cases, blister complications were recorded during the operation (Image 4.25). However, the postoperative course was normal.^8^, ^20^

Difficulties during impression taking

The patient's limited mouth opening challenges the impression procedure (Image 4.21).^22^

Some authors have not been able to perform a full digital protocol including an intraoral scan as part of the prosthodontic management because the scan sensor could not be fitted in the mouth of the patient and saliva could not be aspirated atraumatically.^29^

After implant supported rehabilitation

Most authors describe a normal postoperative course with no complications.^5^, ^23^, ^25^, ^26^ Isolated cases have reported poor oral hygiene^22^ and mucositis at the implant sites, but without additional changes in peri‐implant bone levels.^27^ During the osseointegration period, the rounded healing caps can cause ulcers on the tongue.^24^ A detailed description on hygiene and gingival scores follow‐up can be found in the study published by Agustín‐Panadero in 2019.^31^

Rehabilitation failures have also been described: The metal framework of a patients’ rehabilitation fractured at the solder joint. The fractured section was repaired successfully.^22^ In another patient, the lingual position of the mandibular implants resulted in prognathism, proving a mechanical disadvantage that led to fractures of the abutments. Encouraged by the clinical stability of the implants, more implants were placed in the mandible, improving the occlusal stability and allowing successful rehabilitation.^24^

*Level of evidence*

- Nonanalytic studies (3)

**24**. **What is the best frequency of maintenance recall appointments after implant supported rehabilitation in patients with RDEB?**

*Summary of evidence*

**IMAGE 4.26:**
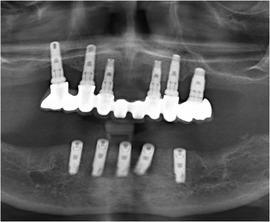
One‐year follow‐up panoramic radiograph

Most authors recommend monitoring the patient 1 and 3 months after implant placement and implant rehabilitation. Thereafter, the patient should be engaged in a monitoring schedule. The appointments should be every 2 or 3 months the first year,^20^, ^21^, ^25^, ^26^, ^27^, ^28^, ^29^, ^31^ and thereafter on a biannual schedule.^30^ Panoramic radiographs should be taken after 6 months, 12 months, and every 12 months thereafter (Image 4.26).^20^, ^21^, ^25^, ^26^, ^31^ Most authors do not remove the rehabilitation to perform the cleaning session. Only one author removed the abutments for hygiene purposes.^24^

*Level of evidence*

- Nonanalytic studies (3)

*Recommendation*

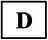
The patients should be clinically evaluated at 1 week, 1 month, and 3 months after the implant surgery.
- After the implant rehabilitation, the patient should be monitored after 1 week and 1 month.
- Dental hygiene sessions should be scheduled every 3 to 6 months thereafter.
- Radiographs should be obtained every 12 months.

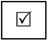
Prosthesis should only be removed if there is a problem, not for standard cleaning.

#### Images

We would like to acknowledge the support of patients, clinicians, and researches from different clinical centers globally for collaborating by providing images for the present guideline. Written informed consent has been obtained for all images where patients can be recognized. Images provided by Dr. Marcelo Guzman‐Letelier, Dr. Antonio Olivares, and Dr. Susanne Krämer.

REFERENCES1

Stavropoulos
F
, 
Abramowicz
S
. Management of the oral surgery patient diagnosed with epidermolysis bullosa: report of 3 cases and review of the literature. J Oral Maxillofac Surg. 2008;66(3):554‐559.1828039410.1016/j.joms.2007.06.6722

Wright
JT
. Oral manifestations of epidermolysis bullosa In: FineJ‐D, ed. Epidermolysis Bullosa Clinical, Epidemiologic, and Laboratory Advances and the Findings of the National Epidermolysis Bullosa Registry. Baltimore: The Johns Hopkins University Press; 1999:236‐256.3

De Benedittis
M
, 
Petruzzi
M
, 
Favia
G
, 
Serpico
R
. Oro‐dental manifestations in Hallopeau‐Siemens‐type recessive dystrophic epidermolysis bullosa. Clin Exp Dermatol. 2004;29(2):128‐132.1498726510.1111/j.1365-2230.2004.01485.x4

Silva
LCP
, 
Cruz
RA
, 
Abou‐Id
LR
, 
Brini
LNB
, 
Moreira
LS
. Clinical evaluation of patients with epidermolysis bullosa: review of the literature and case reports. Spec Care Dentist. 2004;24(1):22‐27.1515705610.1111/j.1754-4505.2004.tb01675.x5

Peñarrocha‐Diago
M
, 
Serrano
C
, 
Sanchis
JM
, 
Silvestre
FJ
, 
Bagán
JV
. Placement of endosseous implants in patients with oral epidermolysis bullosa. Oral Surg Oral Med Oral Pathol Oral Radiol Endodontol. 2000;90(5):587‐590.10.1067/moe.2000.110438110773816

Harel‐Raviv
M
, 
Bernier
S
, 
Raviv
E
, 
Gornitsky
M
. Oral epidermolysis bullosa in adults. Spec Care Dentist. 1995;15(4):144‐148.900291710.1111/j.1754-4505.1995.tb00502.x7

Wright
JT
, 
Fine
JD
, 
Johnson
LB
. Oral soft tissues in hereditary epidermolysis bullosa. Oral Surg Oral Med Oral Pathol. 1991;71(4):440‐446.205232910.1016/0030-4220(91)90426-d8

Larrazabal‐Morón
C
, 
Boronat‐López
A
, 
Peñarrocha‐Diago
M
, 
Peñarrocha‐Diago
M
. Oral rehabilitation with bone graft and simultaneous dental implants in a patient with epidermolysis bullosa: a clinical case report. J Oral Maxillofac Surg. 2009;67(7):1499‐1502.1953142410.1016/j.joms.2009.03.0349

Lindemeyer
R
, 
Wadenya
R
, 
Maxwell
L
. Dental and anaesthetic management of children with dystrophic epidermolysis bullosa. Int J Paediatr Dent. 2009;19(2):127‐134.1925039510.1111/j.1365-263X.2008.00940.x10

Serrano‐Martínez
MC
, 
Bagán J
V
, 
Silvestre
FJ
, 
Viguer
MT
. Oral lesions in recessive dystrophic epidermolysis bullosa. Oral Dis. 2003;9(5):264‐268.1462889410.1034/j.1601-0825.2003.03971.x11

Krämer
SM
, 
Serrano
MC
, 
Zillmann
G
, et al. Oral health care for patients with epidermolysis bullosa ‐ best clinical practice guidelines. Int J Paediatr Dent. 2012;22(Suppl. 1):1‐35.2293790810.1111/j.1365-263X.2012.01247.x12

Kummer
TR
, 
Nagano
HCM
, 
Tavares
SS
, 
Santos
BZD
, 
Miranda
C
. Oral manifestations and challenges in dental treatment of epidermolysis bullosa dystrophica. J Dent Child (Chic). 2013;80(2):97‐100.2401129913

Puliyel
D
, 
Chiu
C
, 
Habibian
M
. Restorative and periodontal challenges in adults with dystrophic epidermolysis bullosa. J Calif Dent Assoc. 2014;42(5):313‐318.2508734914

Feijoo
JF
, 
Bugallo
J
, 
Limeres
J
, et al. Inherited epidermolysis bullosa: an update and suggested dental care considerations. J Am Dent Assoc. 2011;142(9):1017‐1025.2188106710.14219/jada.archive.2011.032115

Reichart
PA
, 
Schmidt‐Westhausen
AM
, 
Khongkhunthian
P
, 
Strietzel
FP
. Dental implants in patients with oral mucosal diseases ‐ a systematic review. J Oral Rehabil. 2016;43(5):388‐399.2668587110.1111/joor.1237316

Diz
P
, 
Scully
C
, 
Sanz
M
. Dental implants in the medically compromised patient. J Dent. 2013;41(3):195‐206.2331371510.1016/j.jdent.2012.12.00817

Candel‐Marti
M‐E
, 
Ata‐Ali
J
, 
Peñarrocha‐Oltra
D
, 
Peñarrocha‐Diago
M
, 
Bagán
J‐V
. Dental implants in patients with oral mucosal alterations: An update. Med Oral Patol Oral Cir Bucal. 2011;16(6):e787‐e793.2119686110.4317/medoral.1705618

Chrcanovic
BR
, 
Gomez
RS
. Dental implants in patients with epidermolysis bullosa: a systematic review. Oral Maxillofac Surg. 2019;23(4):389‐394.3165957110.1007/s10006-019-00802-0PMC684164519
Scottish Intercollegiate Guidelines Network.
, 
Harbour
RT
, 
Forsyth
L
. SIGN 50: A Guideline Developer's Handbook. Ediburgh, Scotland: Scottish Intercollegiate Guidelines Network; 2008:102 p.20

Peñarrocha
M
, 
Rambla
J
, 
Balaguer
J
, et al. Complete fixed prostheses over implants in patients with oral epidermolysis bullosa. J Oral Maxillofac Surg. 2007;65(7):103‐106.10.1016/j.joms.2007.03.0201758635421

Peñarrocha
M
, 
Larrazábal
C
, 
Balaguer
J
, et al. Restoration with implants in patients with recessive dystrophic epidermolysis bullosa and patient satisfaction with the implant‐supported superstructure. Int J Oral Maxillofac Implants. 2007;22(4):651‐655.1792952822

Lee
H
, 
Al Mardini
M
, 
Ercoli
C
, 
Smith
MN
. Oral rehabilitation of a completely edentulous epidermolysis bullosa patient with an implant‐supported prosthesis: a clinical report. J Prosthet Dent. 2007;97(2):65‐69.1734137210.1016/j.prosdent.2006.12.01023

Oliveira
MA
, 
Ortega
KL
, 
Martins
FM
, 
Maluf
PSZ
, 
Magalhes
MG
. Recessive dystrophic epidermolysis bullosa”oral rehabilitation using stereolithography and immediate endosseous implants. Spec Care Dent. 2010;30(1):23‐26.10.1111/j.1754-4505.2009.00117.x2005107124

Müller
F
, 
Bergendal
B
, 
Wahlmann
U
, 
Wagner
W
. Implant‐supported fixed dental prostheses in an edentulous patient with dystrophic epidermolysis bullosa. Int J Prosthodont. 2010;23(1):42‐48.2023489125

Peñarrocha‐Oltra
D
, 
Peñarrocha‐Diago
M
, 
Balaguer‐Martínez
J
, 
Ata‐Ali
J
, 
Peñarrocha‐Diago
M
. Full‐arch fixed prosthesis supported by four implants in patients with recessive dystrophic epidermolysis bullosa. Oral Surg Oral Med Oral Pathol Oral Radiol Endod. 2011;112(2):e4‐10.10.1016/j.tripleo.2011.03.0222168477626

Peñarrocha‐Oltra
D
, 
Aloy‐Prósper
A
, 
Ata‐Ali
J
, 
Peñarrocha‐Diago
M
, 
Peñarrocha‐Diago
M
. Implants placed simultaneously with particulated bone graft in patients diagnosed with recessive dystrophic epidermolysis bullosa. J Oral Maxillofac Surg. 2012;70(1):e51‐e57.2218266110.1016/j.joms.2011.08.03427

Agustín‐Panadero
R
, 
Gomar‐Vercher
S
, 
Peñarrocha‐Oltra
D
, 
Guzmán‐Letelier
M
, 
Peñarrocha‐Diago
M
. Fixed full‐arch implant‐supported prostheses in a patient with epidermolysis bullosa: a clinical case history report. Int J Prosthodont. 2015;28(1):33‐36.2558817010.11607/ijp.409228

Guzmán
M
, 
Jara
CC
, 
Peñarrocha‐Oltra
S
, 
Gomar‐Vercher
S
, 
Peñarrocha‐Diago
M
. Fixed implant‐supported full‐arch prosthesis in epidermolysis bullosa with severe symptoms. J Oral Implantol. 2016;42(6):498‐505.2766910610.1563/aaid-joi-D-14-0010429

Agustín‐Panadero
R
, 
Serra‐Pastor
B
, 
Peñarrocha‐Oltra
D
, 
Peñarrocha‐Diago
M
. Maxillary implant prosthodontic treatment using digital laboratory protocol for a patient with epidermolysis bullosa: a case history report. Int J Prosthodont. 2017;30(4):390‐393.2869721210.11607/ijp.506530

Molina
G
, 
Torassa
DLR
. Implant‐supported dental prostheses in a patient with recessive dystrophic epidermolysis bullosa. Four‐year follow up. Methodo. 2017;2(4):129‐33.31

Agustín‐Panadero
R
, 
Serra‐Pastor
B
, 
Peñarrocha‐Oltra
D
, 
Ferreiroa
A
, 
Peñarrocha‐Diago
M
. Digital scanning for implant‐supported fixed complete arch dental prostheses for patients with epidermolysis bullosa: a case series evaluation. J Prosthet Dent. 2019;122(4):364‐370.3107988510.1016/j.prosdent.2018.11.01932

Hubbard
LD
, 
Mayre‐Chilton
K
. Retrospective longitudinal study of osteoporosis in adults with recessive dystrophic epidermolysis bullosa. Clin Case Reports. 2019;7(1):58‐63.10.1002/ccr3.1898PMC63330673065600933

Peñarrocha
M
, 
Serrano
MC
, 
Sanchis
JM
, et al. Periodoncia. Monográfico de Osteointegración Octubre‐Diciembre
2000;10(4). Artículo Original.

## CHAPTER 5: Sedation and anesthesia for adults and children with epidermolysis bullosa undergoing dental treatment—Clinical practice guidelines

Susanne Krämer | Anne W Lucky | Jemima E Mellerio | Roger Cornwall | Francisca Gamboa | Ignacio Araya | Carolina Arriagada | Rubén Soto | Victoria Clark

### Introduction

Children and adults living with Epidermolysis Bullosa (EB) present fragile skin and mucosa, requiring a special approach from the health care team. When planning a dental procedure under sedation or general anesthesia, the entire clinical team must be aware of the appropriate precautions that individuals with EB may require to receive optimal care. The first clinical practice guideline (CPG) on oral health care for patients with EB was published in 2012.^1^ As new evidence has been published since it has become necessary to update the guideline ensuring that all new data are incorporated, as well as including more experts from different centers around the world to discuss the different treatment options and to work toward establishing the best CPGs. The present guideline has been entrusted by DEBRA International, the worldwide network working on behalf of those living with EB. It follows a standard methodology based on a systematic review of the currently available scientific evidence and a panel discussion.

### Aim

To provide the users with information on the current best practice for managing local anesthesia, principles of sedation and general anesthesia for children and adults with EB undergoing dental treatment.

### Health question covered in the guideline

Do patients with EB undergoing dental treatment under sedation or general anesthesia need specific precautions or treatment modifications compared to healthy individuals to avoid trauma to the skin and mucosa?

### Users

Specialists in Pediatric Dentistry, Special Care Dentistry, Oral and Maxillofacial Surgery, Endodontics, Periodontics, Rehabilitation and General Dental Practitioners, Nurses, Anesthetists, Pediatricians, Dermatologists, and other health care professionals managing patients with EB who need dental treatment under sedation or general anesthesia.

### Target group

These guidelines can be applied to all patients diagnosed with EB regardless of the type of EB. They are therefore applicable to all four major types of EB: EB simplex, junctional EB, dystrophic EB, and Kindler EB.

### Methodology

#### Systematic literature searching

##### Literature sources

A systematic literature review was performed to identify all the literature on sedation and anesthesia for patients with EB undergoing dental treatment. The literature search ranged from 2010 to March 2018. Consulted sources included the electronic databases MEDLINE (2010 to March 2018), EMBASE (2010 to March 2018), CINAHL (2010 to March 2018), The Cochrane Library (2010), DARE (2010), and the Cochrane controlled trials register (CENTRAL) (2010). In addition, hand searching journals, reviewing conference proceedings, and other guidelines sources such as DEBRA International Guideline depository were carried out. The reference lists of all papers for relevant citations were reviewed. When all the relevant studies were identified, they were sent to the experts to review for completeness.

#### Selection criteria of the articles

Selection criteria of the articles: primary or secondary articles in which the main topic is dental care and anesthesia (diagnosis, and⁄or treatment and⁄or prognosis) in patients with EB, published between 2010 and 2018 in any language.

#### Search strategy

To identify studies for this review, detailed search strategies were based on the search strategy developed for MEDLINE; but revised appropriately for each database. The search strategy used a combination of controlled vocabulary and free text terms based on:

#1 "Epidermolysis Bullosa"[Mesh],
#2 ((Epidermolysis[tiab] OR Acantholysis[tiab])) AND Bullosa[tiab]
#3 "Dentistry"[Mesh]
#4 "Oral Health"[Mesh]
#5 "Mouth Diseases"[Mesh]
#6 "Dentistry"[tiab]
#7: #1 OR #2;
#8: #3 OR #4 OR #5 OR #6;
#9: #7 AND #8;
#10: #9 AND ("2010/11/01"[PDat] : "2018/03/01"[PDat]).

#### Methods used for formulating the recommendations

**FIGURE 5.1:**
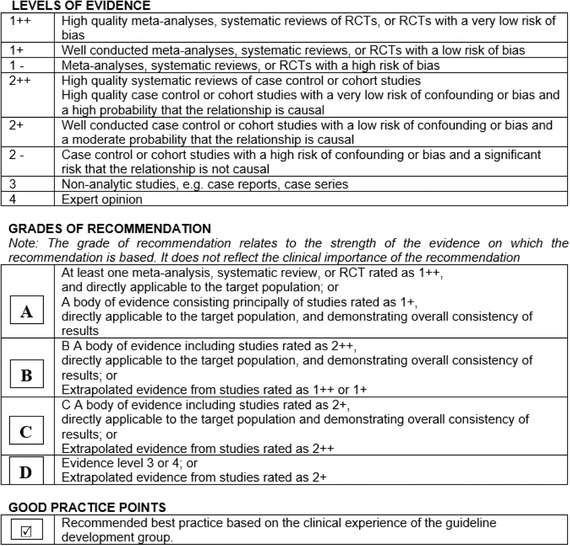
Method used for formulating the recommendations as described on the 50 Guideline Developer's Handbook, NHS Scottish Intercollegiate Guidelines Network SIGN. Revised Edition January 2008^2^

To formulate the recommendations of the selected studies, the SIGN system was used as described on the 50 Guideline Developer's Handbook, NHS Scottish Intercollegiate Guidelines Network SIGN. Revised Edition January 2008^2^ (Figure 5.1).

### Guideline development process

The information from the selected studies was gathered in a draft document by the clinical leads and methodologists. The draft document was analyzed and discussed at a consensus meeting held in Santiago, Chile, on October 23rd, 2018, by the clinical experts guided by the methodologists as described in Tables 2 and 4 (see page 3 and 4).

The consensus report was reviewed by nine external specialists, two members of the multidisciplinary EB team, and three patient groups as described in Tables 3 and 5 on page 3 and 5. Patients and representatives from the DEBRA association groups reviewed the document to ensure that the degree to which the evidence addresses patients' concerns is reflected in the guideline.

The final version was piloted in three centers from three different countries for 3 months (September 2019 to December 2019) as described in Table 6 (page 4).

**TABLE 2.1:** Main oral features of the major EB types

	EB Simplex	Junctional EB	Dominant Dystrophic EB	Recessive Dystrophic EB	Kindler EB
Perioral tissue	Can present ulcers and erosions on the face.	Perioral and perinasal granulation tissue (mainly in children with severe forms).	Can present some ulcers and erosions on the face.	Most patients present ulcers and erosions at different healing stages on their face.	Erosions, crusts, and cheilitis can be observed.
Microstomia (mouth opening)	NDE	Has been reported in 50% to 67% of the patients.	NDE	Progressive microstomia develops. Severe in 80% of the patients.	Can be present. Not all patients will develop this condition.
Oral ulcers	Localized EBS: 7% to 35% of the patients have positive history. Intermediate EBS: 24% to 43% of the patients have positive history. Severe EBS: 59% to 80% of the patients have positive history. Severity tends to lessen with age.	Few lesions on examination, but high history of major intraoral bullae or granulation tissue: 83% to 91%. Slow healing process, intraoral scarring is uncommon.	Present in 20% to 90% of the patients.	Present in 97% of the patients, can affect all intraoral surfaces.	Varies among patients from no ulcers to painful ulcers throughout the oral mucosa.
Denuded tongue	Not reported	Not reported	Not reported	Tongue papillae are absent. Absence of palatal rugae is also reported.	Not reported
Ankyloglossia	No significant scarring observed.	No significant scarring observed.	No significant scarring observed.	Is common, may affect all patients.	No significant scarring observed.
Vestibule obliteration	No significant scarring observed.	No significant scarring observed.	Reduction of keratinized gingiva has been reported.	Severe obliteration of the vestibule is common, may affect all patients.	Partial vestibule obliteration has been described in several patients.
Oral squamous cell cancer (OSCC)	Only one case reported, 41 year old.	Not reported	Not reported	OSCC has been described on tongue, lip, and hard palate. Age range 25 to 54.	OSCC has been reported on hard palate, buccal mucosa, upper and lower lip. Age range 34 to 55 years.
Periodontal disease	NDE	Gingival hyperplasia observed in 50% of the patients.	NDE	Extensive plaque deposits and gingivitis are often observed.	High prevalence, early onset, and fast progression of periodontal disease.
Caries	NDE	NDE	NDE	Significant higher caries scores.	NDE
Generalized Enamel Hypoplasia	Not reported	Generalized Enamel Hypoplasia in all patients. Type and severity vary from pitted to generalized thinning and furrowing of the enamel.	Not reported	Not reported	Not reported
Failure of eruption	Not reported	Can be present.	Not reported	Not reported	Not reported
Occlusal abnormalities	NDE	NDE	NDE	Smaller jaws have been observed on cephalometric studies. Severe crowding is often reported.	NDE

Abbreviation: NDE, No difference expected compared to unaffected population.

### Guideline implementation and monitoring

#### Implementation barriers

According to the context of implementation of this guideline, some barriers to be considered are:

▪ Access to training in EB‐specific issues.
▪ Insufficient availability of health services in some parts of the world.

#### Guideline monitoring and/or auditing criteria

The implementation of these recommendations could be monitored and evaluated through audits and completing of the “CPG Evaluation Form: Pre implementation” available on DEBRA International web page (https://surveyhero.com/c/aabc0100). The panel recommends clinical sites to conduct prepractice audit, implement the CPG, and reaudit to test improvements. Audit tools can be used from SIGN35. DEBRA International would value your feedback on the site findings to continue to improve CPG quality.

#### Further areas of research

- Continuous follow‐up of the recommendations stated in this guideline.

### Guideline updating procedure

The guideline will be updated every 5 years after its second version. If new relevant evidence is detected before the update, the information will be published on the web site http://www.debra-international.org/.

The team in charge of this update will be formed by Prof. Susanne Krämer and Prof. James Lucas in 2025.

### RESULTS

#### Anesthetic management of children and adults with epidermolysis bullosa undergoing dental treatment

Children and adults living with EB can receive dental treatment using local anesthesia, sedation, or general anesthesia. The decision of which anesthetic modality to be used is decided after a detailed risk assessment is undertaken as well as a detailed discussion with the patient and/or family members for children. The discussion usually includes risks, benefits, advantages, and disadvantages of each technique, as well as the alternatives based on what is available in the local specialized services. There are many factors that can influence the final decision such as the patient's age,^3^ extent of dental treatment required,^36^, ^37^ and the patient's acute stress reaction.^3^ It is important to highlight that sedation should only be performed if all the expertise and resources for emergency access of critical airway are available.

For patients with mild forms of EB; simple, atraumatic dental procedures can be performed safely under local anesthesia as the technique of choice. General anesthesia is therefore reserved for extensive, more complex procedures or for patients with severe forms of EB. The support of an experienced medical team is crucial in this setting.

#### 5.1 Local anesthesia

Provided a risk assessment is undertaken and microstomia can be managed, all dental treatment techniques can be provided successfully under local anesthesia, including multiple extractions, implants, RCT, and restorations.^38^, ^39^, ^40^

The benefits of local anesthesia when compared to general anesthesia are avoidance of potential damage to the airway and the provision of prolonged postoperative pain relief. Additional benefits have also been reported^8^ including lower cost of care and absence of hospitalization, thus reducing the risks of general anesthesia and hospital acquired infections. Less mucosal damage is usually reported when procedures are performed under local anesthesia when compared to procedures performed under general anesthesia.

**IMAGE 5.1:**
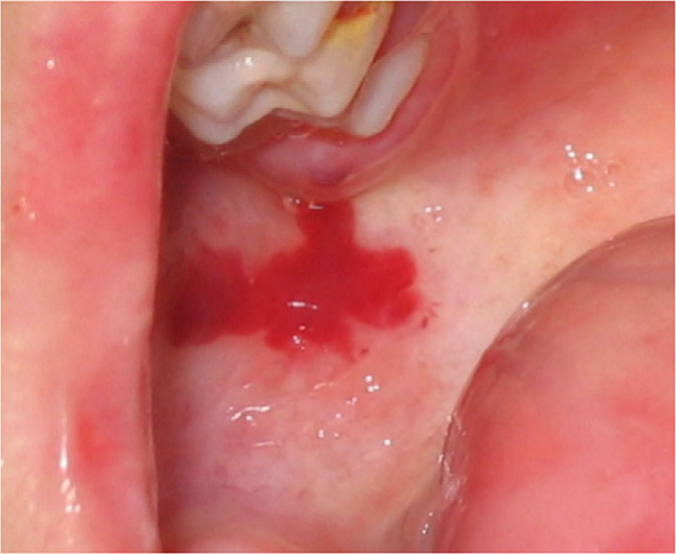
Blood filled bulla at an injection site


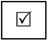
 Topical anesthesia in gel form can be used normally.


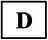
 To avoid bullae formation, the anesthetic solution must be injected deeply into the tissues and at a slow rate in order to avoid having the liquid cause mechanical separation of the tissue^40^, ^42^, ^43^ (Image 5.1).


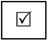
 Computer‐controlled local anesthetic delivery systems, such as The Wand STA^®^ (Milestone Scientific Inc., Livingston, NJ, USA) and Dentapen^®^ (Juvaplus SA, Geneva, Switzerland), can be beneficial in providing anesthesia.


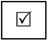
 Iatrogenic bullae may develop following local anesthetic injection.^38^, ^40^, ^44^, ^45^, ^46^, ^47^, ^48^ These have to be drained immediately with a sterile needle or by a cut with scissors to avoid lesion expansion due to fluid pressure. The overlying mucous membrane (roof of the blister) should not be removed as it acts like a natural dressing and aids healing, reducing pain, and minimizing the risk of exogenous infection (Image 5.2).


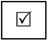
 Postoperative instruction must highlight that the patients should not bite, rub, or traumatize their lips while under the effect of local anesthesia.

**IMAGE 5.2:**
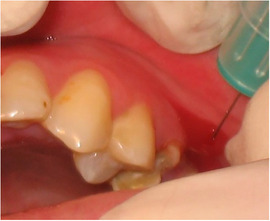
Drainage of a blood‐filled bulla with a sterile needle

#### 5.2 Sedation

A wide spectrum of sedation techniques is available for use in dentistry. Regulations and guidelines might vary among countries; and need to be followed according to local authorities. Within the scientific literature, only few authors describe the provision of dental care using sedation in patients with EB. Examples of these are dental extractions following intramuscular sedation in children with dystrophic EB^16^ and adults with intermediate recessive dystrophic EB (RDEB).^17^

Nitrous oxide inhalation sedation is an example of a technique widely used in dentistry. In EB, it has been rated as successful for pain control during dressing changes for children at home.^18^ However, reports of its use for the dental care of children with EB are lacking. If used, the skin protection precautions described in this article should be considered.

The recommendation based on the clinical experience of the guideline development group is:


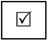
 Moderate sedation should only be performed if all the expertise and resources for emergency access of a critical airway are available.

#### 5.3 General anesthesia

**IMAGE 5.3:**
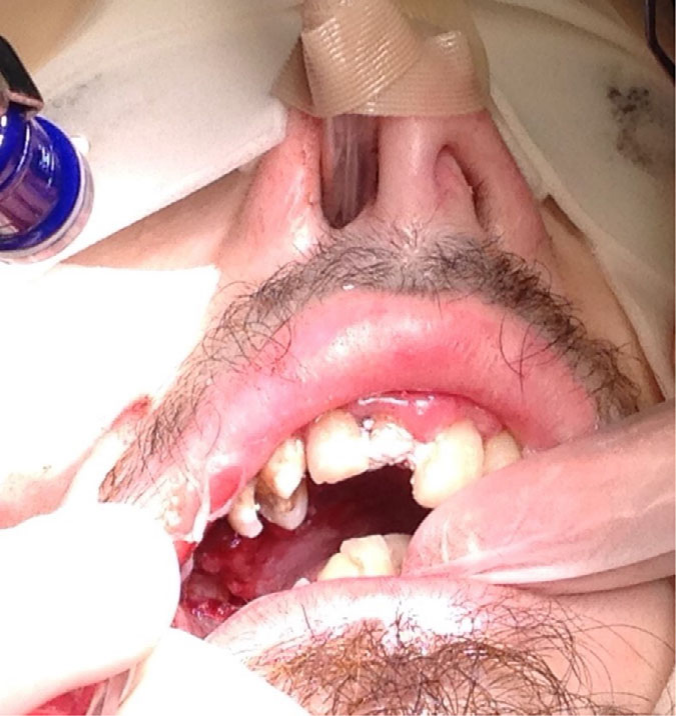
Limited oral access due to microstomia during a general anesthesia session using nasal intubation


Treatment under general anesthesia aids the provision of extensive dental treatment and multiple extractions when microstomia, vestibular obliteration, severity of soft tissue fragility, and patients’ ability to cope with the procedure are challenging.^42^, ^52^ Nevertheless, the provision of dental treatment under general anesthesia can remain challenging as severe tissue fragility can lead to intraoperative generalized mucosal sloughing secondary to minor trauma or tissue manipulation, even if all precautions are taken.^52^, ^53^, ^54^ Microstomia will not improve under general anesthesia and the surgeon will still be faced with limited oral access even if nasal intubation, which is the intubation of choice, is used. Access to the working area will remain a challenge (Image 5.3).^37^, ^55^ Some authors recommend combined procedures using the same general anesthesia jointly with other surgical specialities,^23^ eg, esophageal dilatation^20^ and bandage changes after hand surgery.^4^ The risk of transient bacteremia due to dental treatment should be discussed among surgical teams to decide whether it is possible to combine specific procedures.

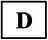
 When planning a procedure under general anesthesia, the patient's physician should be consulted.^1^


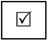
 The availability of an anesthetic team with experience in EB is preferred. If this is not available, the use of local anesthesia or referral to a specialist center could be considered as alternatives.

#### 5.4 Special considerations when planning and performing dental treatment under general anesthesia

Patients with EB, especially RDEB, often require multiple surgical procedures within the oral cavity, gastrointestinal tract, and on the hands. Among the main challenges for the anesthetist are microstomia, ankyloglossia,^24^ perioral scarring, limited head and neck mobility secondary to scars and contractures, laryngeal stenosis, and esophageal strictures. All of these challenges increase the risk of regurgitation and aspiration during anesthesia. In addition, as an intraoperative complication, there is the risk of acute airway obstruction due to oropharyngeal bulla development while manipulating the airway.^4^ The best practice is to work as part of a multidisciplinary group with dermatologists, surgeons, and nurses caring for patients with EB under general anaesthesia.^15^

##### Skin protection

**IMAGE 5.4:**
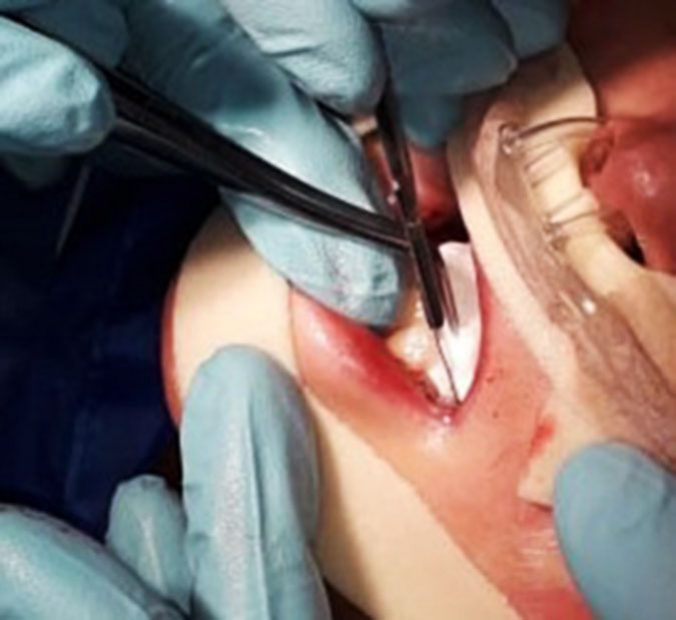
Image taken during a sedation session: Skin areas touched by the surgeons and in contact with nasal cannula are well protected with soft silicone foam dressings. Lips are well lubricated with an emollient

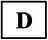
Adhesives should be avoided if possible.^36^, ^37^, ^48^, ^58^, ^59^, ^60^ If silicone‐based adhesives are not available, minimally adherent products and adhesive remover could be tried. Table 5.1 presents a variety of dressings available for patients with EB.

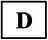
Preoperative preparation should include wound care.^28^

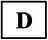
Skin areas that will be touched by the oral surgeon or anesthetist must be covered with nonadherent dressings^48^, ^62^, ^63^, ^64^ as skin sloughing easily occurs during patient handling (Image 5.4).^3^ Consideration should include any patient handling including chin lifts during intubation.

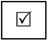
Some surgeons apply ointments or emollients on their surgical gloves to avoid blistering the skin, while other surgeons choose to apply these lubricants on the skin and specific instruments only. Examples of ointments, lubricants, and emollients are described in Chapter 3.

**TABLE 5.1:** Protection dressings for the management of patients with epidermolysis bullosa

Contact layers	Mepitel^®^ ^A^ Mepitac^®^ ^A^ Mepilex^®^ Transfer ^A^	Silflex^®^ ^B^ Adaptic Touch™ ^C^ Siltape^®^ ^B^
Bielastic silicone dressing	Spycra^®^ Protect ^D^	
Bordered foam dressings	Mepilex^®^ Border ^A^ Mepilex^®^ Border Lite^A^ Biatain^®^ Silicone Lite ^E^ Biatain^®^ Border Lite ^E^	Allevyn Gentle Border ^F^ Allevyn Lite ^F^ KerraFoam™ ^G^ UrgoTul Absorb Border ^H^
Soft silicone mesh	Mepitel^®^ ^A^ Mepitel^®^ One ^A^	Adaptic Touch™ ^C^ Cuticell^®^ Contact ^I^
Lipidocolloid dressings	Urgotul^®^ ^H^	Restore^®^ ^J^
Soft silicone foams	Mepilex^®^ ^A^ Mepilex^®^ Lite ^A^	Mepilex^®^ Transfer ^A^
Polymeric membrane	PolyMem^®^ ^K^	
Soft silicone fixation tape	Siltape^®^ ^B^	Mepitac^®^ ^A^
Fixation bandages	CoFlex^®^ Haft ^L^ Soft‐One^®^ ^M^	Acti‐Wrap ^N^
Modified absorbent pads	Telfa™ ^O^ Restore^®^ ^J^	Mesorb^®^ ^A^
Adhesive removers	Adapt™ Medical Adhesive Remover ^J^ Appeel^®^ ^P^	Niltac Adhesive Remover™ ^Q^ Brava^®^ Adhesive Remover ^E^

These examples of wound dressing brand names are only an aid to become familiar with the many available products as published by Pope and collaborators in the 2012 “A consensus approach to wound care in epidermolysis bullosa”^32^ and Denyer and collaborators in 2017 “International Consensus Best Practice Guidelines for Skin and Wound Care in Epidermolysis Bullosa”.^33^ Updated information should be sought at DEBRA International Guidelines section.

^A^ Mölnlycke Health Care AB, Gothenburg, Sweden.

^B^ Advancis Medical, Nottingham, United Kindgom

^C^ Systagenix Wound Management Ltd, Gatwick, UK

^D^ Reskin Medical NV, Tessenderlo, Belgium

^E^ Coloplast A/S, Humlebaek, Denmark

^F^ Smith & Nephew PLC, London, UK

^G^ Crawford Healthcare Ltd, Knutsford Cheshire, UK

^H^ Laboratories URGO, Dijon, France

^I^ BSN Medical GmbH, Hamburg, Germany

^J^ Hollister Incorporated, Libertyville, IL, USA

^K^ Ferris Mfg Corp, Fort Worth, TX, USA

^L^ Milliken, Andover, MA, USA

^M^ Snøgg, Vennesla, Norway

^N^ Activa Healthcare Ltd, Burton upon Trent, UK

^O^ Covidien, Dublin, Ireland

^P^ CliniMed Limited, High Wycombe, UK

^Q^ ConvaTec Inc, Flintshire, UK

##### Patient positioning and moving

**IMAGE 5.5:**
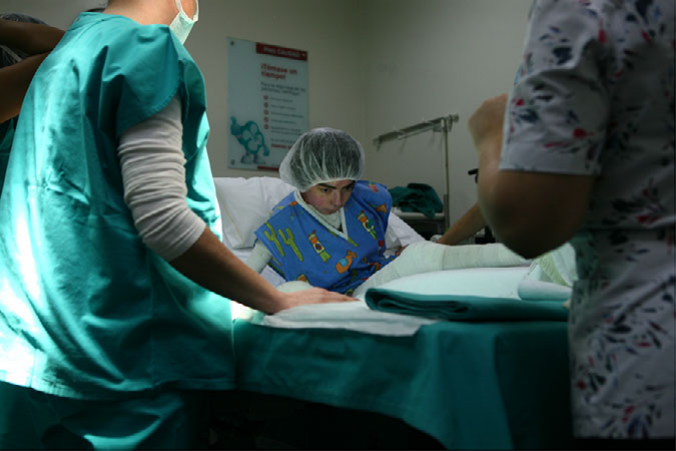
Patient transferring to operating table

**IMAGE 5.6:**
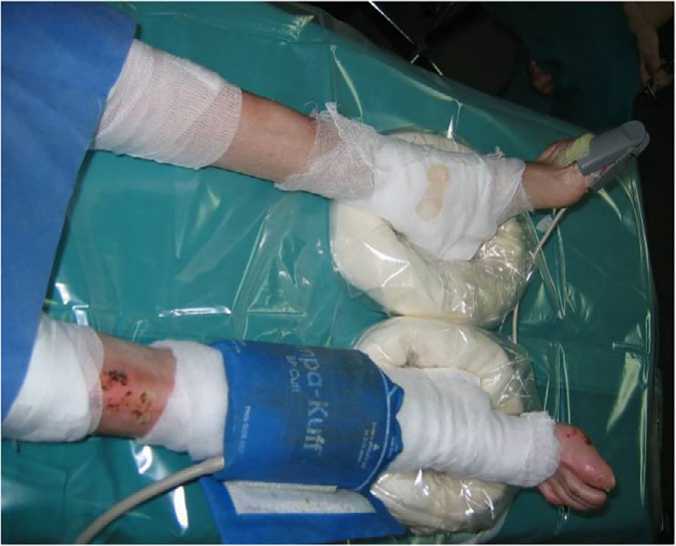
Pressure point padding during general anesthesia

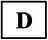
Stretchers and tables should be padded (eg, with a wipeable foam, a wool blanket, seat cushion, or mattress topper).^48^, ^53^, ^55^, ^58^

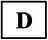
Patients should be allowed to place themselves on the operating table (if possible) prior to anaesthesia^55^, ^63^ to avoid blister formation while transferring (Image 5.5).

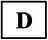
Pressure points should be protected with nonadherent or petrolatum‐coated bandages,^22^ padding,^28^ or soft gauze^30^ (Image 5.6).

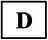
Transfer and position changing should be done by moving the blanket, not by sliding the patient, as handling the patient could cause damage.^48^, ^64^ Slide sheets can be used as an aid.

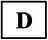
Patients should not be lifted under the arms, but be lifted from under the buttocks and the back of the neck.^34^

**IMAGE 5.7:**
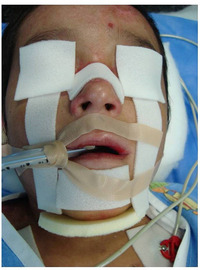
Eye protection with nonadherent dressing. All the areas to be touched by the surgeon are protected with nonadherent dressing

**IMAGE 5.8:**
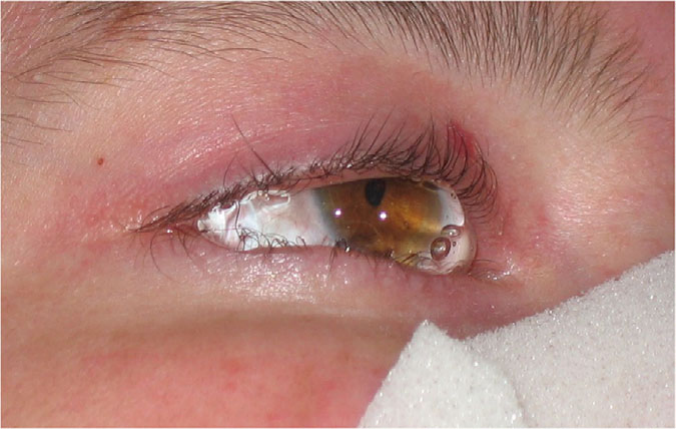
Eye protection in a patient with incomplete eyelid closure: ophthalmic ointment is applied and covered with nonadherent pads. Procedure is repeated every hour or as needed to maintain eye moisture

**IMAGE 5.9:**
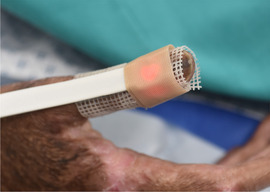
Finger pulse oximeter probe secured with a nonadhesive tape

**IMAGE 5.10:**
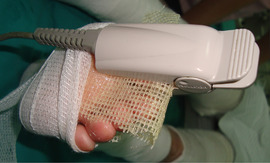
Pulse oximeter on a toe covered with Mepitel^®^ (Mölnlycke, Gothenburg, Sweden) to protect skin

**IMAGE 5.11:**
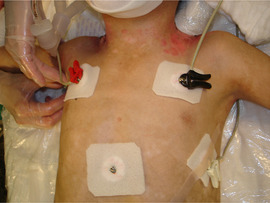
ECG leads secured with nonadhesive dressings

**IMAGE 5.12:**
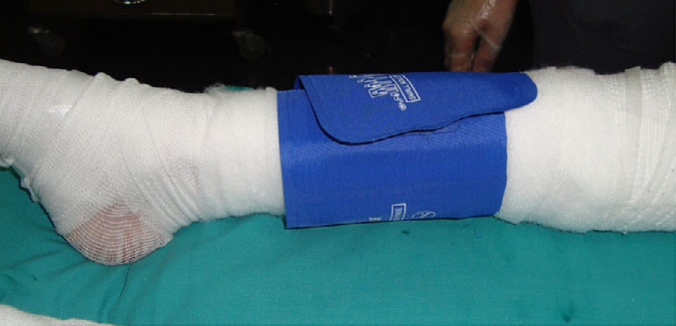
Noninvasive blood pressure cuff applied on a protected leg

##### Eye protection

A high incidence of perioperative corneal damage has been observed; therefore, eye ointment and dressings should always be used for protection.^24^ Once the patient is supine under general anesthesia and before placing the drapes, examine if there is proper eyelid closure. Lagophthalmos (incomplete eyelid closure) is very common in patients with EB due to eyelid skin scaring and may be worsened by ectropion. Choice of eye lubrication will depend on the patient's eyelid occlusion and availability of specific materials. If the eyelids can be closed normally, the eyes should be lubricated with an ophthalmic gel such as sodium hyaluronate 2 mg/mL and draped with a nonadherent dressing under the head drapes (Image 5.7). If silicone‐based bandages are not available, a soft moisture gauze can be used.^36^, ^37^, ^55^, ^63^ If there is lagophthalmos, the eyes need to be thoroughly lubricated. This can be done by applying a petroleum‐based ophthalmic ointment and maintaining the eyes covered with nonadherent pads between applications that should be done every hour (Image 5.8). Upon recovery, patients should be informed about transient blurred vision due to the lubricant.^33^ If a patient uses therapeutic bandage contact lenses, these should not be removed. The eyes should be lubricated with any preservative‐free eye lubricant such as sodium hyaluronate 1 mg/m: every 20 to 30 minutes and use the head dressing as described above.^35^ Contact lenses for refractive purpose should be removed before the procedure.

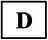
Eyes need to be well lubricated throughout the procedure as described above.^36^, ^37^, ^55^, ^57^, ^63^, ^64^

*Monitoring*

***Pulse Oximeter***:

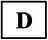
If using a standard disposable finger/toe probe, any adhesive part should be removed (cutoff) and a specific nonadhesive tape^36^, ^64^ or lubricated gauze^48^, ^53^ used to secure the pulse oximeter sensor (Image 5.9). Clip‐type pulse oximetry can be placed on the ear lobe.^55^, ^69^ If a clip‐type pulse oximeter is used on a digit, the digit can be wrapped with a contact layer (listed in Table 5.1), petrolatum dressings, or commercial plastic food wrap to avoid skin damage (Image 5.10).^33^

***Electrocardiogram (ECG)***

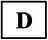
To secure the ECG leads, the adhesive parts should be removed and the leads fixed using a nonadhesive tape^36^, ^59^ allowing only the lubricated central portion to contact the patient's skin (Image 5.11).^36^, ^48^, ^53^, ^59^, ^62^

***Noninvasive blood pressure***:

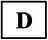
The cuff must be applied over an extremity that is well wrapped with nonadherent material,^31^ bandages, or cotton^37^, ^48^, ^53^, ^55^, ^63^ (Image 5.12).

***Capnography***:

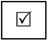
If capnography needs securing/fixation, nonadhesive tape should be used (Table 5.1).

***Tourniquet***

- Avoid use of an elastic tourniquet or glove to minimize skin trauma.^33^

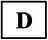
A tourniquet should be placed over a gauze wrapped around the extremity or by minimal manual pressure with lubricated hands, avoiding shearing forces.^48^, ^63^, ^66^
- Avoid excessive rubbing during skin preparation using a “dabbing” motion for topical antimicrobials.^33^

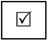
The tourniquet should be released slowly and carefully to avoid skin sloughing off.

**IMAGE 5.13:**
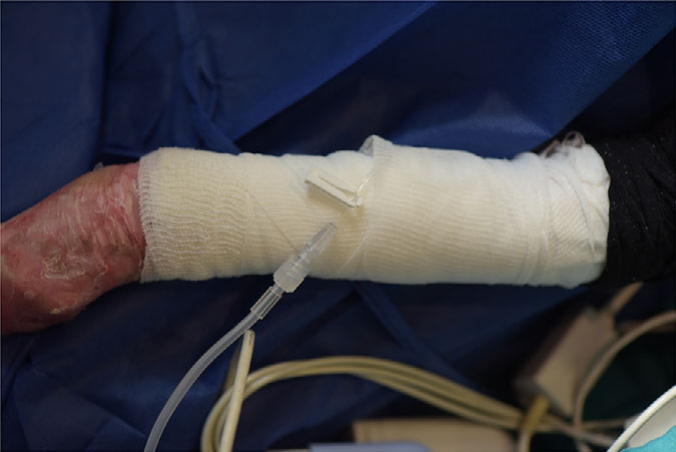
Intravenous catheter secured with gauze

***Intravenous line securing***

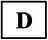
Intravenous catheters can be secured with nonadherent tape and then wrapped with gauze^36^, ^64^ (Image 5.13). A foam dressing or petroleum‐coated gauze can be placed between the skin and intravenous hub to avoid skin damage: it should be securely wrapped with self‐adherent or nonadhesive elastic bandages around the extremity, eg, Coban^®^ (3M, St. Paul, MN, USA) or Coflex^®^ (Andover Healthcare, Salisbury, MA, USA).^37^, ^48^, ^53^, ^58^, ^63^ The wrist can be secured using a foam‐padded wrist support board.^20^

**IMAGE 5.14:**
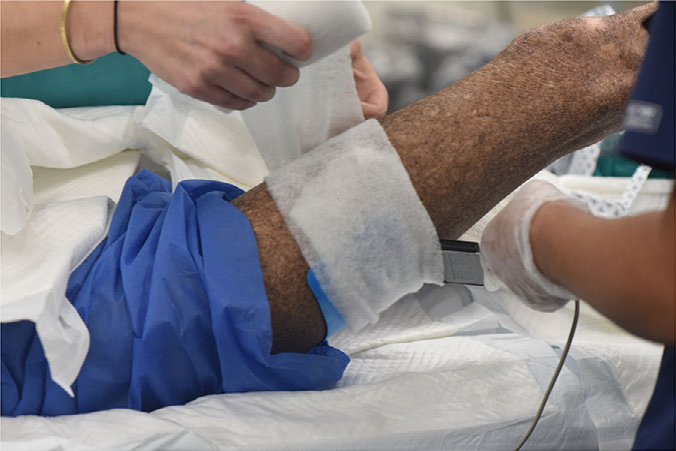
Fixing the electrosurgery pad with nonadhesive technique

**IMAGE 5.15:**
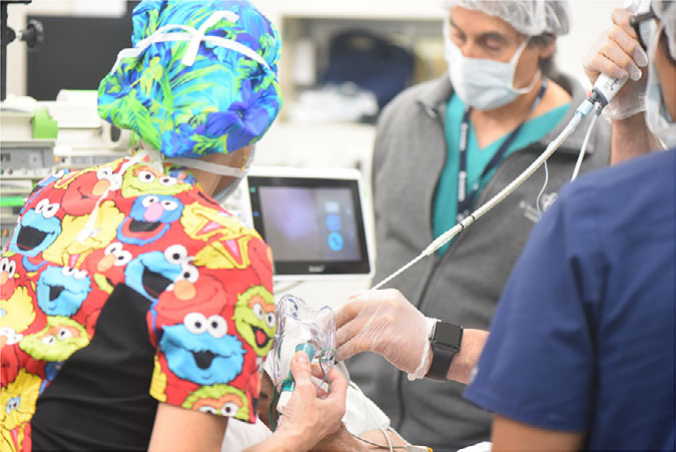
Fiber optic bronchoscopy in a patient with challenging intubation due to severe microstomia

***Instrument preparation***

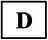
Facemasks, endotracheal tubes, and nasal cannulas need to be well lubricated to reduce friction. This can be done with petrolatum,^36^, ^41^, ^59^, ^60^ warm saline solution,^55^, ^69^ or other appropriate water‐soluble emollients.

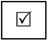
If electrosurgery is planned, the adhesive border of the electrosurgery grounding pad (inactive dispersive electrode) needs to be removed. The pad can be fixed using a nonadhesive tape allowing only the central portion to contact the patient's skin (Image 5.14).

***Airway management***

For safely maintaining an airway, further bullae and erosions must be avoided.^30^ Nasal as well as oral intubation are reported in the literature.^36^, ^55^, ^64^ A nasal intubation would be the first choice as it provides a more spacious surgical field for dental treatment when compared to an oral tube, and can also be secured more easily without tape than the oral tube.^22^

Both video‐assisted laryngoscopy^22^ and fiberoptic bronchoscopy^36^, ^48^, ^60^, ^61^, ^64^ have been successfully used to aid intubation. Cases of intubation during spontaneous ventilation with a fiberoptic bronchoscope have also been reported.^4^ Minimal chin lift and head tilt should be exerted and gentle manipulation of the head with a hand below the occiput and the jaw must be considered.^36^ Slow and gentle manipulation reduces tissue damage (Image 5.15).

- Nasal intubation is the first choice.^22^
- Specific recommendations to aid intubation include: a smaller sized laryngoscope^26^ and a small size cuffed endotracheal tube.^31^

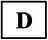
Nonadhesive tape should be used to secure the endotracheal tube (Table 5.1, Image 5.3).^22^
- A throat pack (oropharyngeal pack) must be placed for any dental procedure.^36^, ^59^, ^64^, ^69^ The throat pack should be lubricated with water soluble lubricants, as, for example, Surgilube^®^ (Fougera, Melville, NY, USA) to reduce the risk of it adhering to the mucosa. If lubricants are not available, the throat pack could be soaked with water to reduce the risk of adherence.

**IMAGE 5.16:**
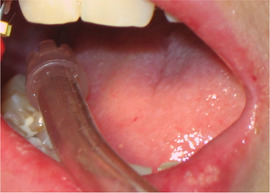
Suction tip leaned on tooth surface to prevent mucosal sloughing

**IMAGE 5.17:**
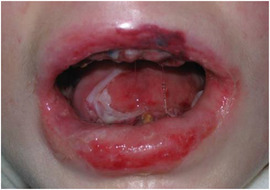
Mucosal sloughing after extensive dental surgery

***Surgical site***

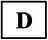
The surgical site should not be scrubbed. Disinfection solutions should be poured, gently dabbed, or sprayed on the skin.^37^, ^48^

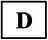
All perioral tissues and commissures should be well lubricated.^36^, ^64^ Lubrication can be done with petrolatum or any other ointment or emollient (Images 5.4, 3.21 [in Chapter 3], and 4.6 [in Chapter 4]).

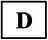
Suction: Bullae formation or epithelial sloughing can occur upon contact with the suction tip.^20^ If possible, the suction tip should be leaned on hard tissue, ie, on tooth or bone surface (Image 5.16).^24^ Vacuum suction can cause extensive mucosal sloughing, its use needs to be very gentle (Image 5.17).

***Patient discharge***

The time of discharge after dental surgery varies. While some reports in the literature noted discharge on the same day as the surgery,^36^ others waited 24 hours postoperatively and some even 3 days postprocedure.^55^, ^59^, ^61^ The time to discharge will depend on the extent of the surgery and the potential benefits of keeping the patient in hospital will need to be weighed against the risks of hospital‐acquired infections.

***Complications***

In order to have a well‐informed risk/benefit discussion with the patient and their family, it is important to know the complications reported in the literature on general anesthesia in patients with EB. In a review of 121 surgical procedures, no death or other major perioperative anesthetic complication occurred.^15^ Another series of 344 surgical procedures under general anesthesia at a reference center for EB reported the following complications related to anesthesia: postoperative nausea/vomiting: 8 (2.3%), new bullae: 7 (2.0%), regurgitation: 2 (0.6%), and corneal ulcers: 1 (0.3%).^30^ Other studies describe the development of new blisters as the most common postoperative complication.^36^, ^48^ Significant injury after poor soft tissue handling can occur when inexperienced members of the team are not aware of the risks of handling patients with EB, eg, inadvertent taping of the eyelids.^30^ Maxillary alveolar process fracture secondary to laryngoscopy was reported in a patient with severe generalized RDEB with poor bone health, severe microstomia, and prominent upper incisors.^37^

As to the intraoral mucosa, generalized sloughing secondary to minor trauma or tissue manipulation can occur even if all precautions are taken. Patients with severe fragility will still develop intraoperative mucosal sloughing secondary to retraction and minor trauma of the procedure itself^52^, ^53^, ^54^ (Image 5.17). During the postoperative healing period, patients might experience their lips sticking together if both lips have substantial damage. Therefore, patients should be advised to continuously lubricate their lips and corners of the mouth with lubricants, petrolatum or other emollients, for example, Vaseline^®^ (Unilever, USA), Linovera^®^ (B.Braun), or other emollients available locally. Performing mouth opening, lip, and tongue movement exercises is also important to maintain oral functions.

#####    

###### Images

We would like to acknowledge the support of patients, clinicians, and researchers from different clinical centers globally for collaborating by providing images for the present guideline. Written informed consent has been obtained for all images where patients can be recognized.

REFERENCES34

Krämer
SM
, 
Serrano
MC
, 
Zillmann
G
, et al. Oral health care for patients with epidermolysis bullosa ‐ best clinical practice guidelines. Int J Paediatr Dent. 2012;22(SUPPL. 1):1‐35.2293790810.1111/j.1365-263X.2012.01247.x35
Scottish Intercollegiate Guidelines Network.
, 
Harbour
RT
, 
Forsyth
L
. SIGN 50: A Guideline Developer's Handbook. Ediburgh, Scotland: Scottish Intercollegiate Guidelines Network; 2008:102 p.36

Rekka
P
, 
Swathi
S
, 
Prabhu
Vr
, 
Ramesh
S
. Dental and anesthetic management of a child with epidermolysis bullosa. J Indian Soc Pedod Prev Dent. 2011;29(2):155.2191195610.4103/0970-4388.8469037

Blázquez Gómez
E
, 
Garcés Aletá
A
, 
Monclus Diaz
E
, et al. Manejo anestésico en paciente pediátrico con vía aérea difícil afectado de epidermólisis ampollosa distrófica. Rev Esp Anestesiol Reanim. 2015;62(5):280‐284.2549714810.1016/j.redar.2014.08.00338

Finke
C
, 
Haas
N
, 
Czarnetzki
BM
. [Value of dental treatment in interdisciplinary management of a child with epidermolysis bullosa dystrophica hereditaria (Hallopeau‐Siemens)]. Hautarzt. 1996;47(4):307‐310.865531810.1007/s00105005042139

Silva
LCP
, 
Cruz
RA
, 
Abou‐Id
LR
, 
Brini
LNB
, 
Moreira
LS
. Clinical evaluation of patients with epidermolysis bullosa: review of the literature and case reports. Spec Care Dentist. 2004;24(1):22‐27.1515705610.1111/j.1754-4505.2004.tb01675.x40

Peñarrocha‐Diago
M
, 
Serrano
C
, 
Sanchis
JM
, 
Silvestre
FJ
, 
Bagán
JV
. Placement of endosseous implants in patients with oral epidermolysis bullosa. Oral Surg Oral Med Oral Pathol Oral Radiol Endodontol. 2000;90(5):587‐590.10.1067/moe.2000.1104381107738141

Torres
CP
, 
Gomes‐Silva
JM
, 
Mellara
TS
, 
Carvalho
LP
, 
Borsatto
MC
. Dental care management in a child with recessive dystrophic epidermolysis bullosa. Braz Dent J. 2011;22(6):511‐516.2218964810.1590/s0103-6440201100060001242

Wright
JT
. Oral manifestations of epidermolysis bullosa In: FineJ‐D, ed. Epidermolysis Bullosa Clinical, Epidemiologic, and Laboratory Advances and the Findings of the National Epidermolysis Bullosa Registry. Baltimore: The Johns Hopkins University Press; 1999:236‐256.43

Peñarrocha
M
, 
Rambla
J
, 
Balaguer
J
, et al. Complete fixed prostheses over implants in patients with oral epidermolysis bullosa. J Oral Maxillofac Surg. 2007;65(7):103‐106.10.1016/j.joms.2007.03.0201758635444

Olsen
CB
, 
Bourke
LF
. Recessive dystrophic epidermolysis bullosa. Two case reports with 20‐year follow‐up. Aust Dent J. 1997;42(1):1‐7.907863810.1111/j.1834-7819.1997.tb00087.x45

Siqueira
MA
, 
de Souza Silva
J
, 
Silva
FWGde P
, et al. Dental treatment in a patient with epidermolysis bullosa. Spec Care Dent. 2008;28(3):92‐95.10.1111/j.1754-4505.2008.00012.x1848965546

Oliveira
TM
, 
Sakai
VT
, 
Candido
LA
, 
Silva
SMB
, 
Machado
MAAM
. Clinical management for epidermolysis bullosa dystrophica. J Appl Oral Sci. 2008;16(1):81‐85.1908929510.1590/S1678-77572008000100016PMC432728647

Serrano Martínez
C
, 
Silvestre Donat
FJ
, 
Bagán Sebastián
JV
, 
Peñarrocha Diago
M
, 
Alió Sanz
JJ
. Epidermólisis ampollosa hereditaria a propósito del manejo odontológico de tres casos clínicos. Med Oral. 2001;6(1):48‐56.1148813148

Lin
Y‐C
, 
Golianu
B
. Anesthesia and pain management for pediatric patients with dystrophic epidermolysis bullosa. J Clin Anesth. 2006;18(4):268‐271.1679742810.1016/j.jclinane.2005.11.00449

Korolenkova M
V
. [Dental treatment in children with dystrophic form of epidermolysis bullosa]. Stomatologiia (Mosk). 2015;94(2):34‐36.2614547510.17116/stomat201594234-3650

Puliyel
D
, 
Chiu
C
, 
Habibian
M
. Restorative and periodontal challenges in adults with dystrophic epidermolysis bullosa. J Calif Dent Assoc. 2014;42(5):313‐318.2508734951

Ingelmo
P
, 
Wei
A
, 
Rivera
G
. Nitrous oxide for procedural analgesia at home in a child with epidermolysis bullosa. Pediatr Anesth. 2017;27(7):776‐778.10.1111/pan.131502849752052

Azrak
B
, 
Kaevel
K
, 
Hofmann
L
, 
Gleissner
C
, 
Willershausen
B
. Dystrophic epidermolysis bullosa: oral findings and problems. Spec Care Dentist. 2006;26(3):111‐115.1677418810.1111/j.1754-4505.2006.tb01433.x53

Stavropoulos
F
, 
Abramowicz
S
. Management of the oral surgery patient diagnosed with Epidermolysis Bullosa: report of 3 cases and review of the literature. J Oral Maxillofac Surg. 2008;66(3):554‐559.1828039410.1016/j.joms.2007.06.67254

Kaslick
RS
, 
Brustein
HC
. Epidermolysis bullosa. Review of the literature and report of a case. Oral Surg Oral Med Oral Pathol. 1961;14:1315‐1330.1445415910.1016/0030-4220(61)90263-855

Al‐Abadi
A
, 
Al‐Azri
SA
, 
Bakathir
A
, 
Al‐Riyami
Y
. Dental and anaesthetic challenges in a patient with dystrophic epidermolysis bullosa. Sultan Qaboos Univ Med J. 2016;16(4):e495‐e499.2800389910.18295/squmj.2016.16.04.016PMC513546456

Lanschuetzer
CM
, 
Nischler
E
, 
Diem
A
, et al. Therapeutical Approaches In: FineJ‐D, HintnerH, eds. Life with Epidermolysis Bullosa (EB). Vienna: Springer Vienna; 2009:209‐308. 10.1007/978-3-211-79271-1_3.57

Krämer
SM
. Oral care and dental management for patients with epidermolysis bullosa. Dermatol Clin. 2010;28(2):303‐309.2044749510.1016/j.det.2010.02.02158

Album
MM
, 
Gaisin
A
, 
Lee
KWT
, et al. Epidermolysis bullosa dystrophica polydysplastica: a case of anesthetic management in oral surgery. Oral Surg Oral Med Oral Pathol. 1977;43(6):859‐872.26667910.1016/0030-4220(77)90078-059

Mello
BZF
, 
Neto
NL
, 
Kobayashi
TY
, et al. General anesthesia for dental care management of a patient with epidermolysis bullosa: 24‐month follow‐up. Spec Care Dent. 2016;36(4):237‐240.10.1111/scd.121702693663260

Berens
RJ
, 
Scott
JP
. Use of modified Statlock^TM^ device for securing an endotracheal tube in facial burn‐like conditions. A A Case Rep. 2015;5(5):69‐71.2632303210.1213/XAA.000000000000017361

Chuang
LC
, 
Hsu
CL
, 
Lin
SY
. A fixed denture for a child with epidermolysis bullosa simplex. Eur J Paediatr Dent. 2015;16(4):315‐318.2663725762

Lindemeyer
R
, 
Wadenya
R
, 
Maxwell
L
. Dental and anaesthetic management of children with dystrophic epidermolysis bullosa. Int J Paediatr Dent. 2009;19(2):127‐134.1925039510.1111/j.1365-263X.2008.00940.x63

Ames
WA
, 
Mayou
BJ
, 
Williams
KN
, 
Williams
K
. Anaesthetic management of epidermolysis bullosa. Br J Anaesth. 1999;82(5):746‐751.1053655410.1093/bja/82.5.74664

Yoon
RK
, 
Ohkawa
S
. Management of a pediatric patient with epidermolysis bullosa receiving comprehensive dental treatment under general anesthesia. Pediatr Dent. 2012;34(3):251‐253.2279516165

Pope
E
, 
Lara‐Corrales
I
, 
Mellerio
J
, et al. A consensus approach to wound care in epidermolysis bullosa. J Am Acad Dermatol. 2012;67(5):904‐917.2238703510.1016/j.jaad.2012.01.016PMC365540366

Denyer
J
, 
Pillay
E
, 
Clapham
J
. Best Practice Guidelines Skin and wound care in Epidermolysis Bullosa [Internet]. London; 2017
www.woundsinternational.com
67

Gonzalez
ME
. Evaluation and treatment of the newborn with epidermolysis bullosa. Semin Perinatol. 2013;37(1):32‐39.2341976110.1053/j.semperi.2012.11.00468

Rashad
R
, 
Weed
MC
, 
Quinn
N
, 
Chen
VM
. Extended wear bandage contact lenses decrease pain and preserve vision in patients with Epidermolysis Bullosa: case series and review of literature. Ocul Immunol Inflamm. 2020;28(3):379‐383.3098612910.1080/09273948.2019.158747269

Esfahanizade
K
, 
Mahdavi
AR
, 
Ansari
G
, 
Fallahinejad Ghajari
M
, 
Esfahanizadeh
A
. Epidermolysis bullosa, dental and anesthetic management: a case report. J Dent (Shiraz, Iran). 2014;15(3):147‐152.PMC41498982519166570

George
M
, 
Martinez
AE
, 
Mellerio
JE
, 
Nandi
R
. Maxillary alveolar process fracture complicating intubation in a patient with epidermolysis bullosa. Pediatr Anesth. 2009;19(7):706‐707.10.1111/j.1460-9592.2009.02995.x19638122
